# Correction: Genetic differentiation of *Oncomelania hupensis robertsoni* in hilly regions of China: Using the complete mitochondrial genome

**DOI:** 10.1371/journal.pntd.0014719

**Published:** 2026-09-11

**Authors:** Jing Song, Hongqiong Wang, Shizhu Li, Peijun Qian, Wenya Wang, Meifen Shen, Zongya Zhang, Jihua Zhou, Chunying Li, Zaogai Yang, Yuwan Hao, Chunhong Du, Yi Dong

The affiliation for the 11^th^ author is incorrect. Yuwan Hao is not affiliated with #5 but with #4: National Key Laboratory of Intelligent Tracking and Forecasting for Infectious Diseases, National Institute of Parasitic Diseases at Chinese Center for Disease Control and Prevention, Chinese Center for Tropical Diseases Research, NHC Key Laboratory of Parasite and Vector Biology, WHO Collaborating Center for Tropical Diseases, National Center for International Research on Tropical Diseases, Shanghai, People’s Republic of China.
