# Supplementary material for: Genetic differentiation of Oncomelania hupensis robertsoni in hilly regions of China: Using the complete mitochondrial genome
Source: PLoS Negl Trop Dis. 2024 Nov 26;18(11):e0012094. doi: 10.1371/journal.pntd.0012094 (PMC11630586; doi:10.1371/journal.pntd.0012094)
Supplement: S1 File — Fig A. Habitat environment of O. hupensis in sampling site of NJ1. Fig B. Habitat environment of O. hupensis in sampling site of NJ2. Fig C. Habitat environment of O. hupensis in sampling site of MD1. Fig D. Habitat environment of O. hupensis in sampling site of XY2. Fig E. Habitat environment of O. hupensis in sampling site of EY1. Fig F. Habitat environment of O. hupensis in sampling site of EY2. Fig G. Habitat environment of O. hupensis in sampling site of DL2. Fig H. Habitat environment of O. hupensis in sampling site of DL3. Fig I. Habitat environment of O. hupensis in sampling site of WS1. Fig J. Habitat environment of O. hupensis in sampling site of HQ1. Fig K. Habitat environment of O. hupensis in sampling site of YS3. Fig L. Habitat environment of O. hupensis in sampling site of GC1. Fig M. Habitat environment of O. hupensis in sampling site of GC2. Fig N. Habitat environment of O. hupensis in sampling site of CX2. (DOCX) [file pntd.0012094.s001.docx]

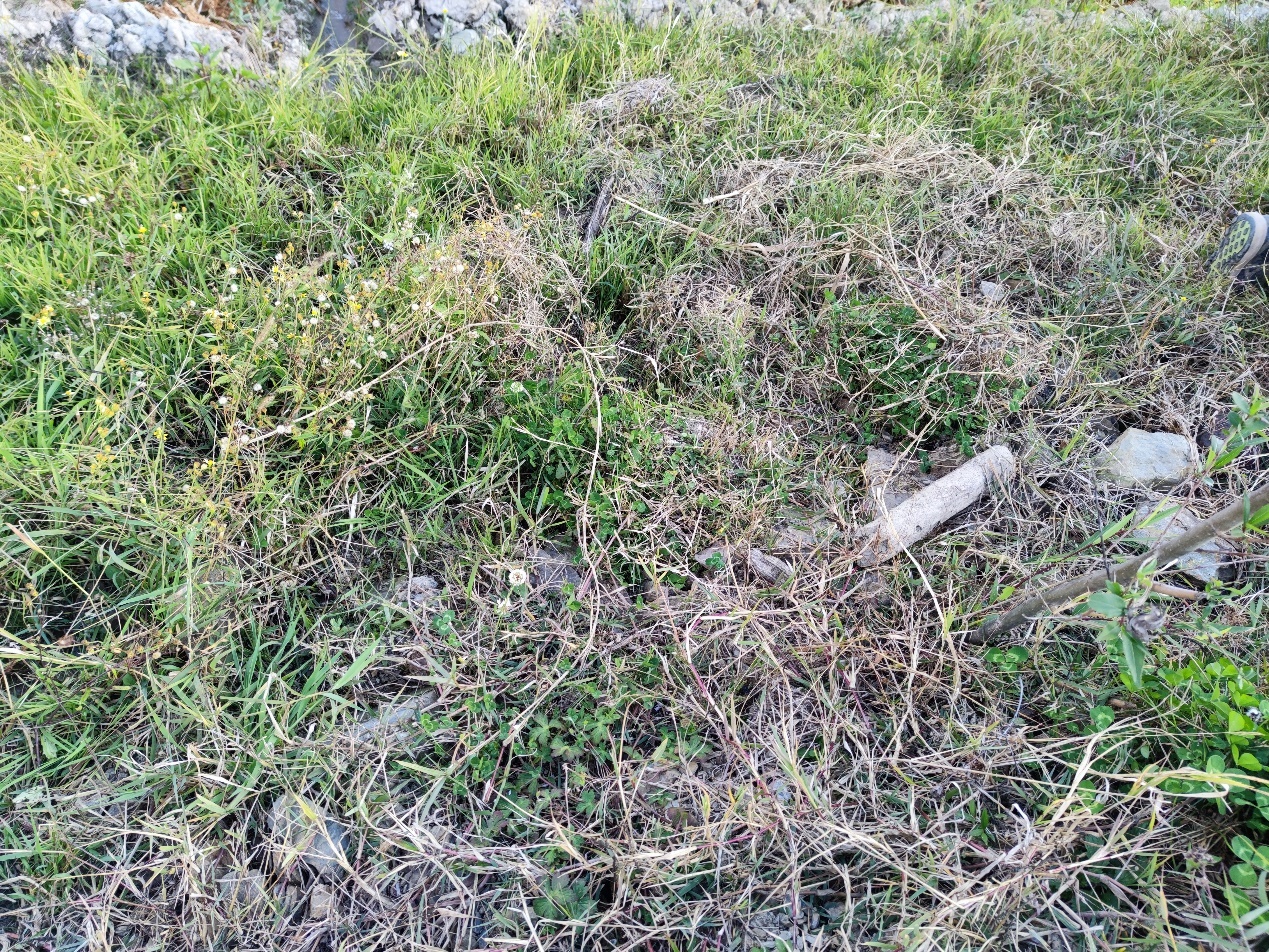


Fig A. Habitat environment of *O. hupensis* in sampling site of NJ1


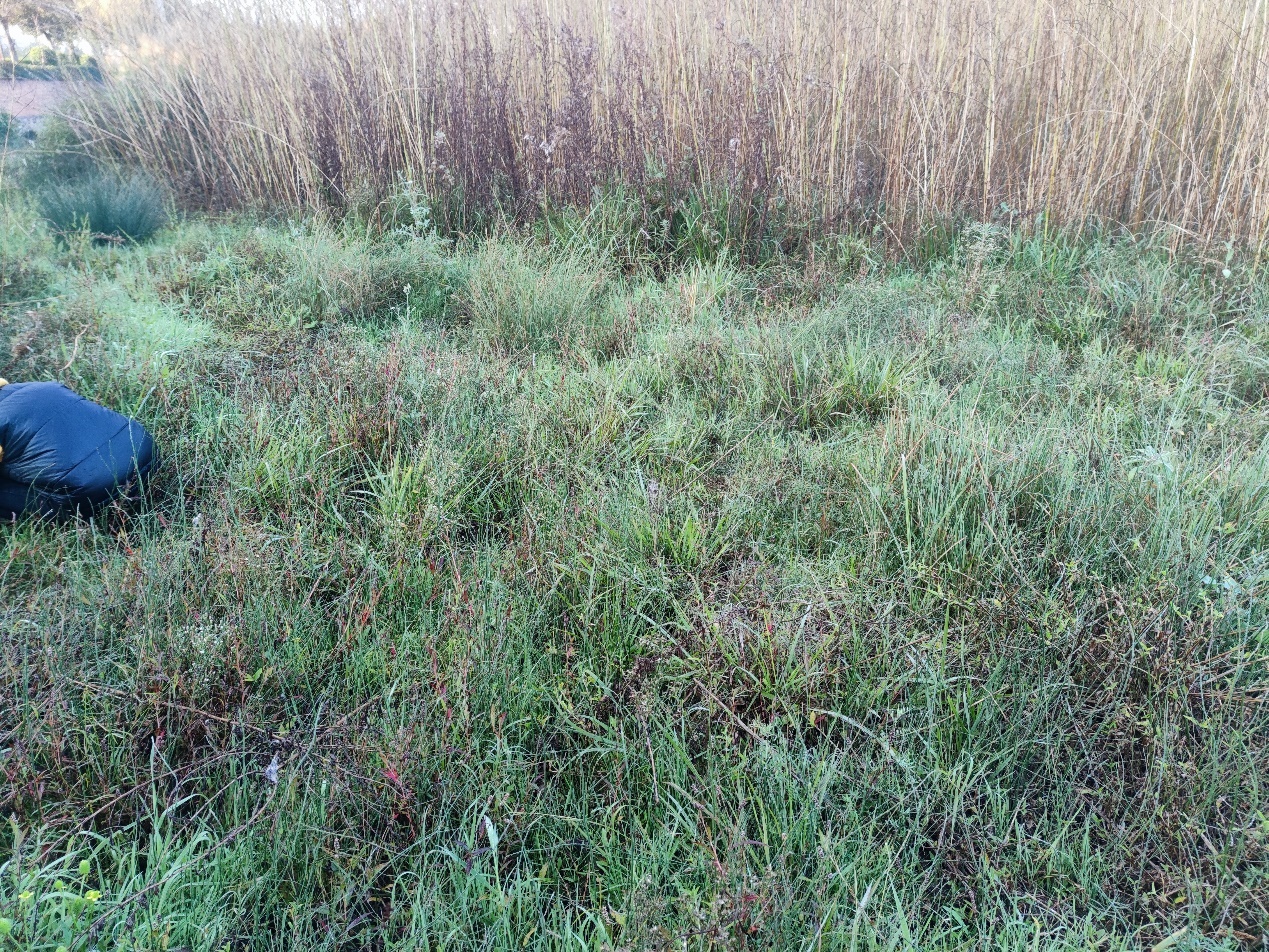


Fig B. Habitat environment of *O. hupensis* in sampling site of NJ2


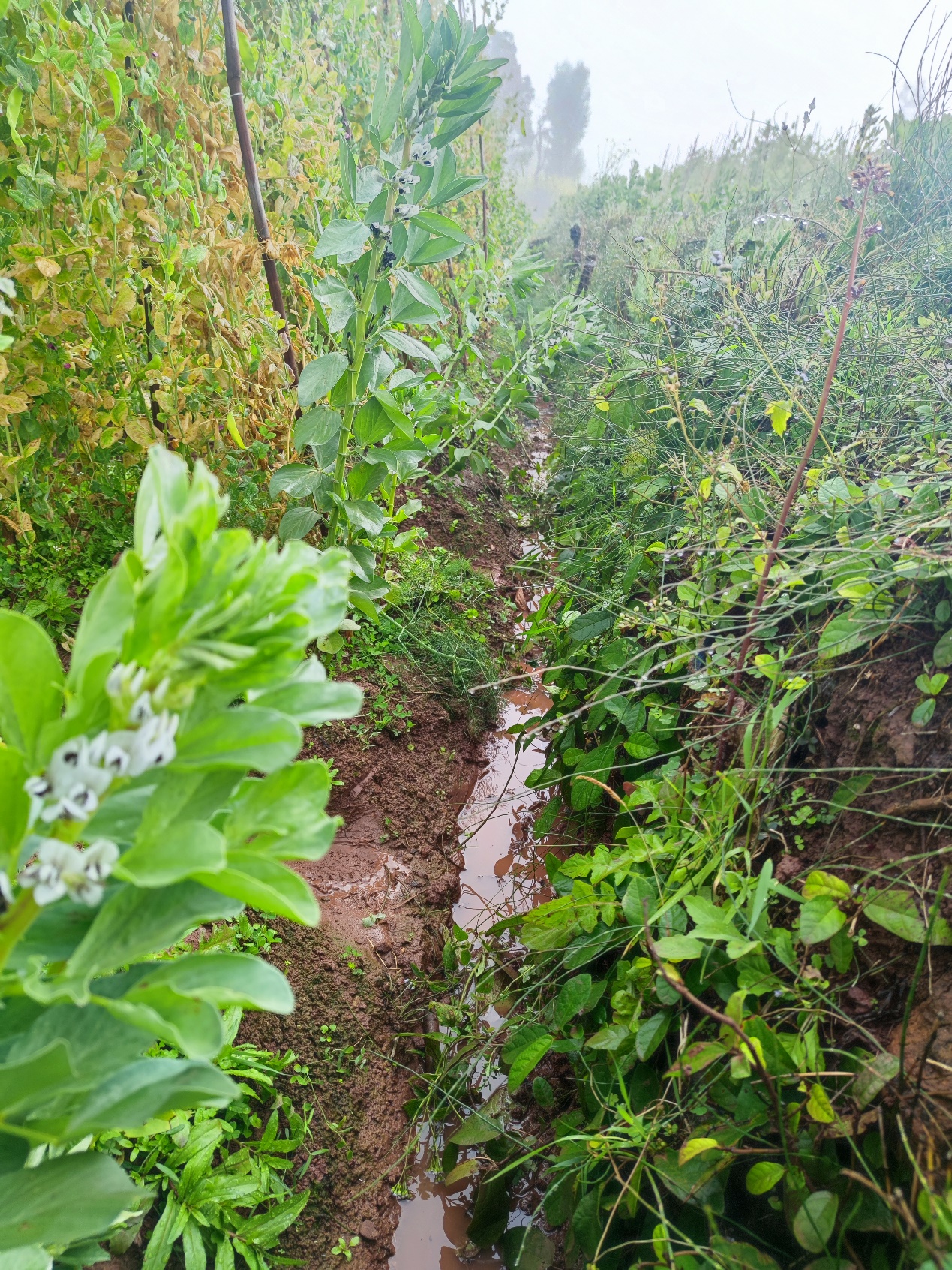

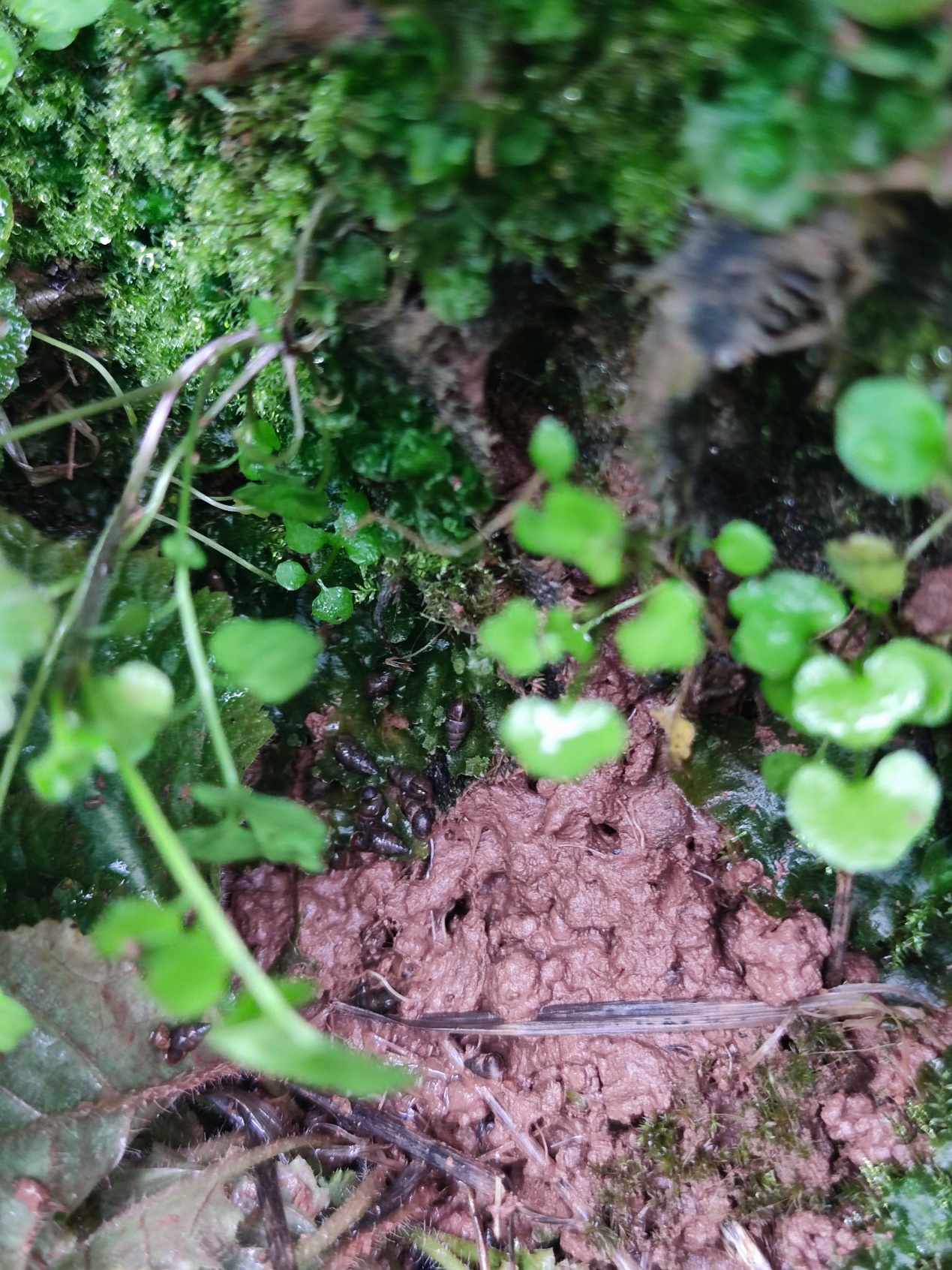


Fig C. Habitat environment of *O. hupensis* in sampling site of MD1


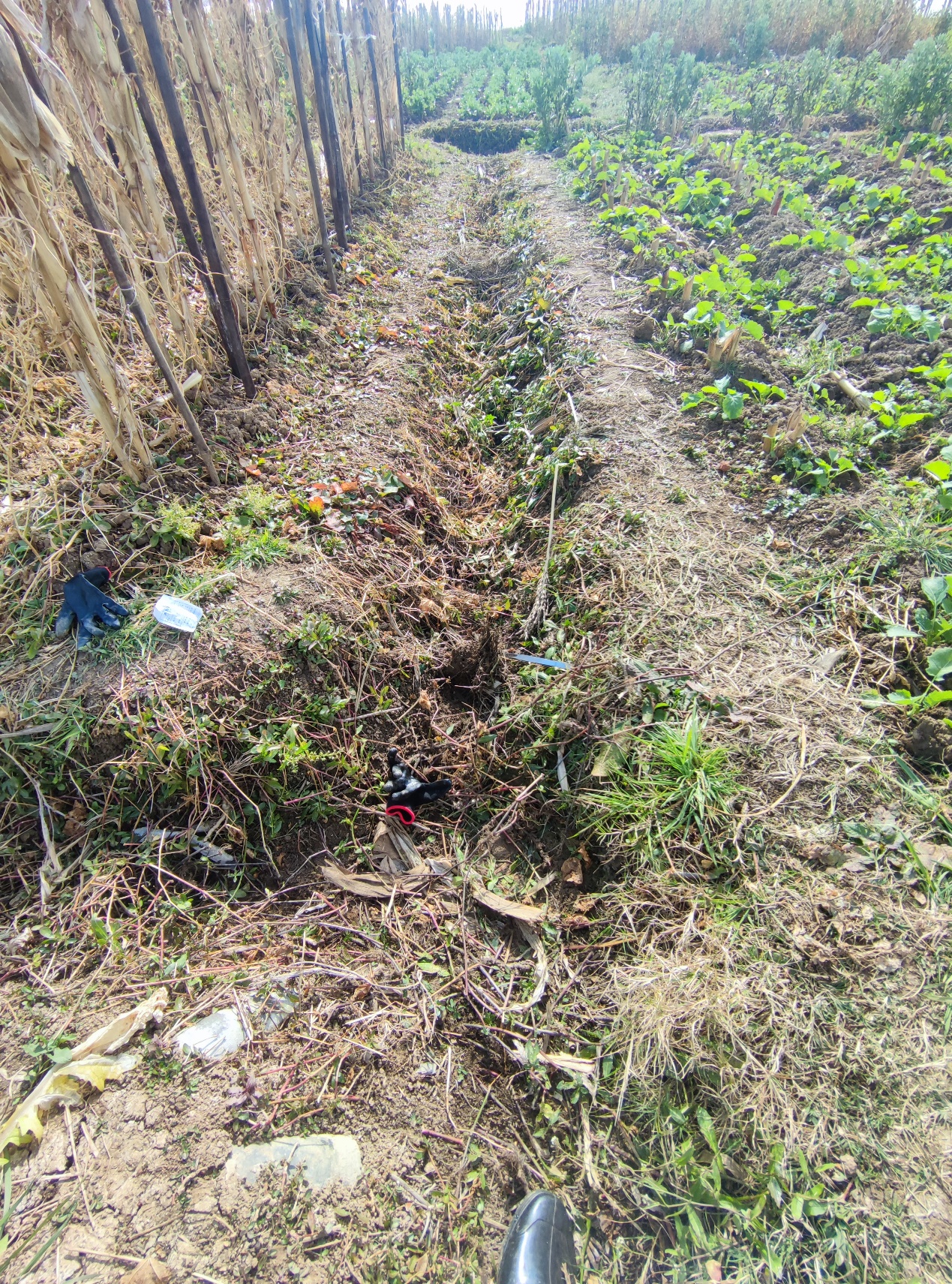

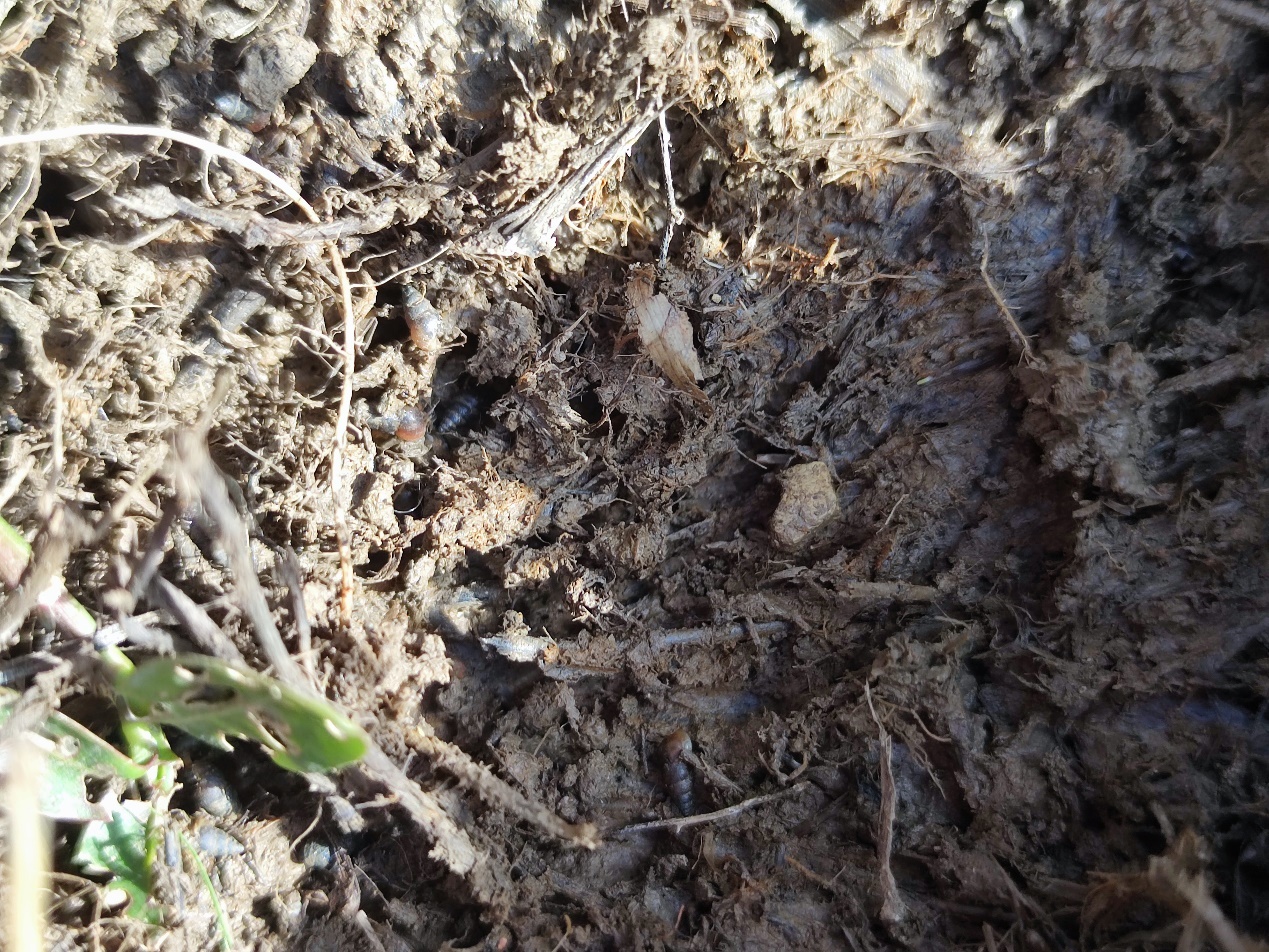


Fig D. Habitat environment of *O. hupensis* in sampling site of XY2


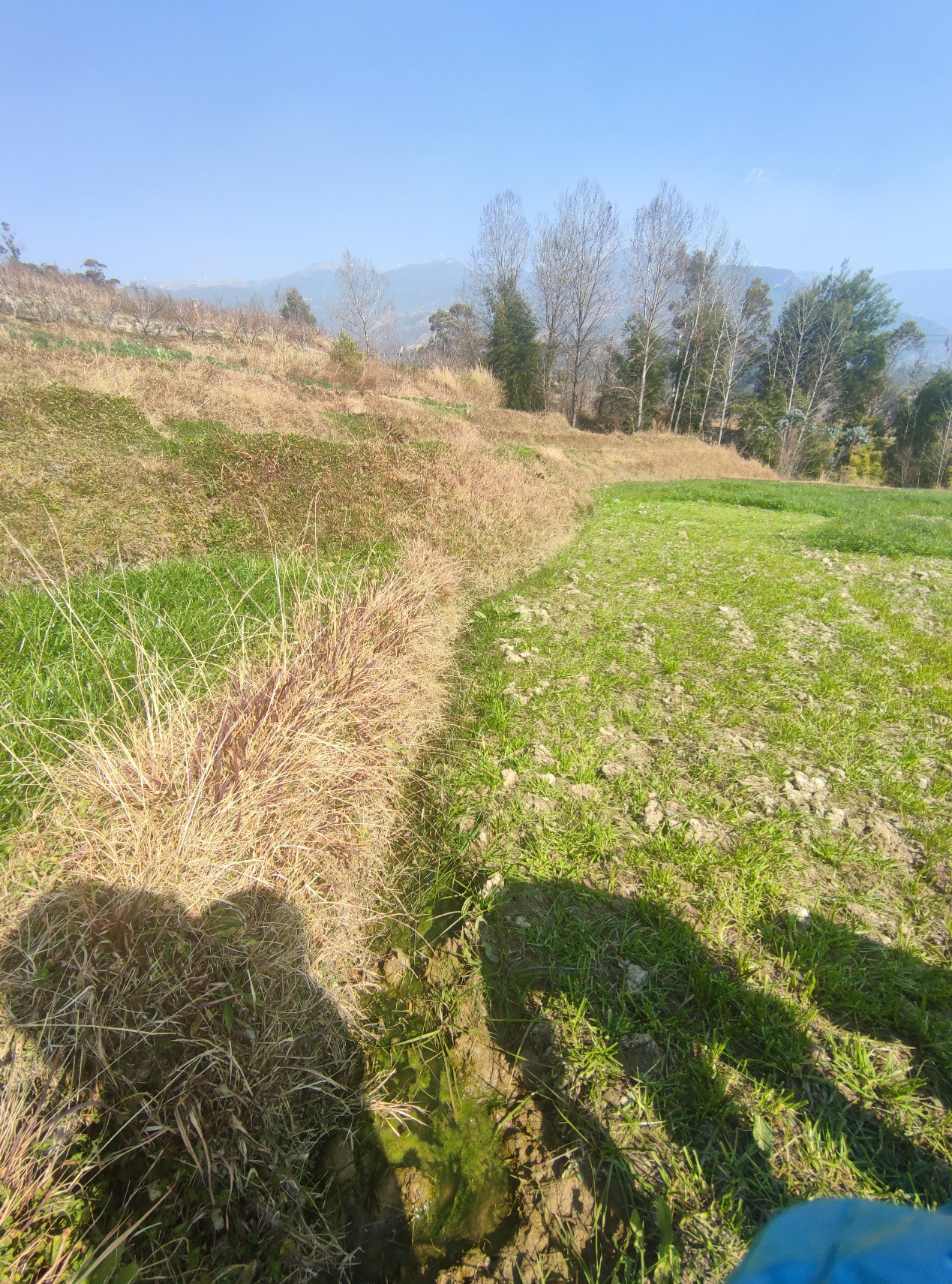


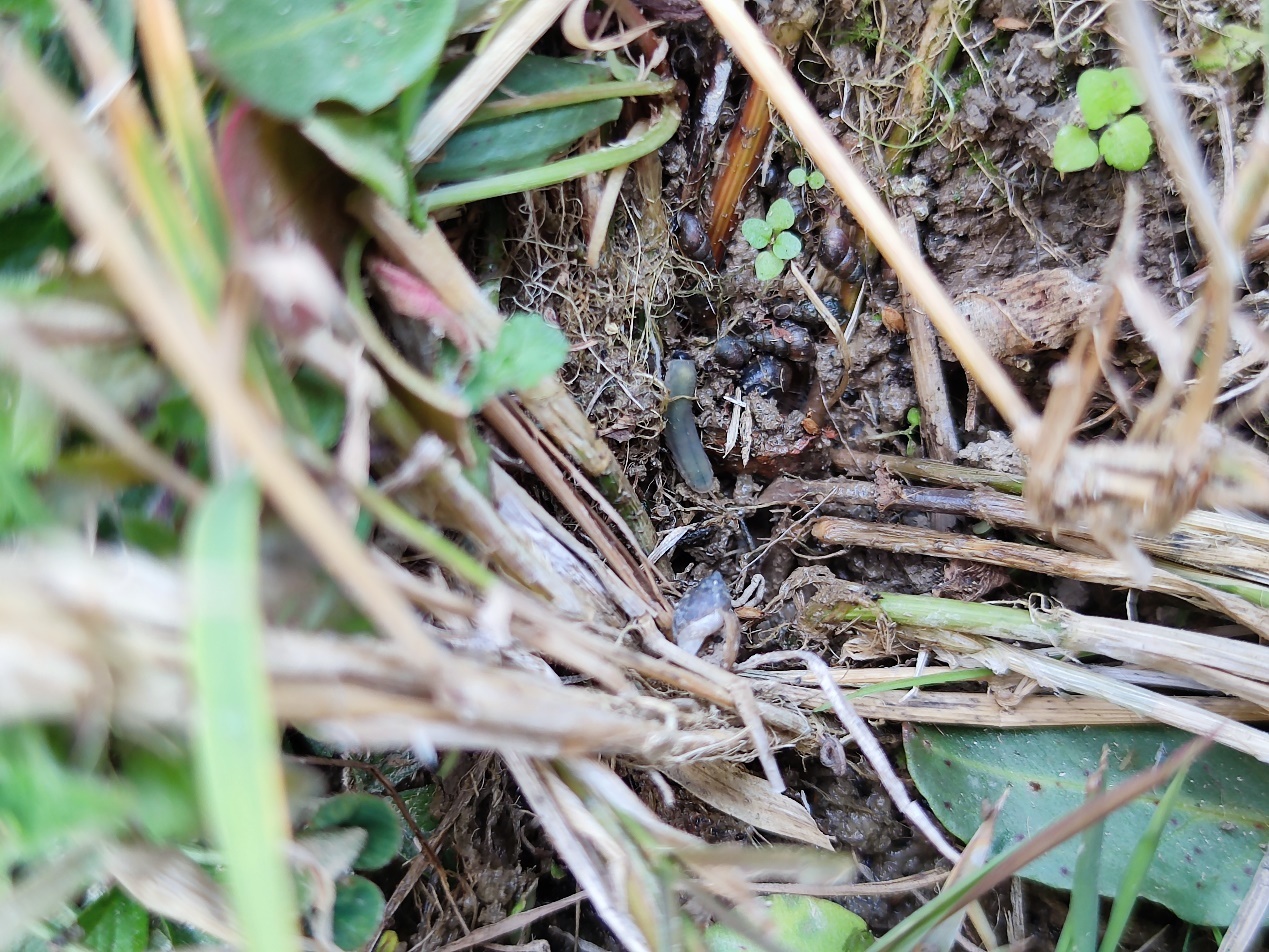


Fig E. Habitat environment of *O. hupensis* in sampling site of EY1


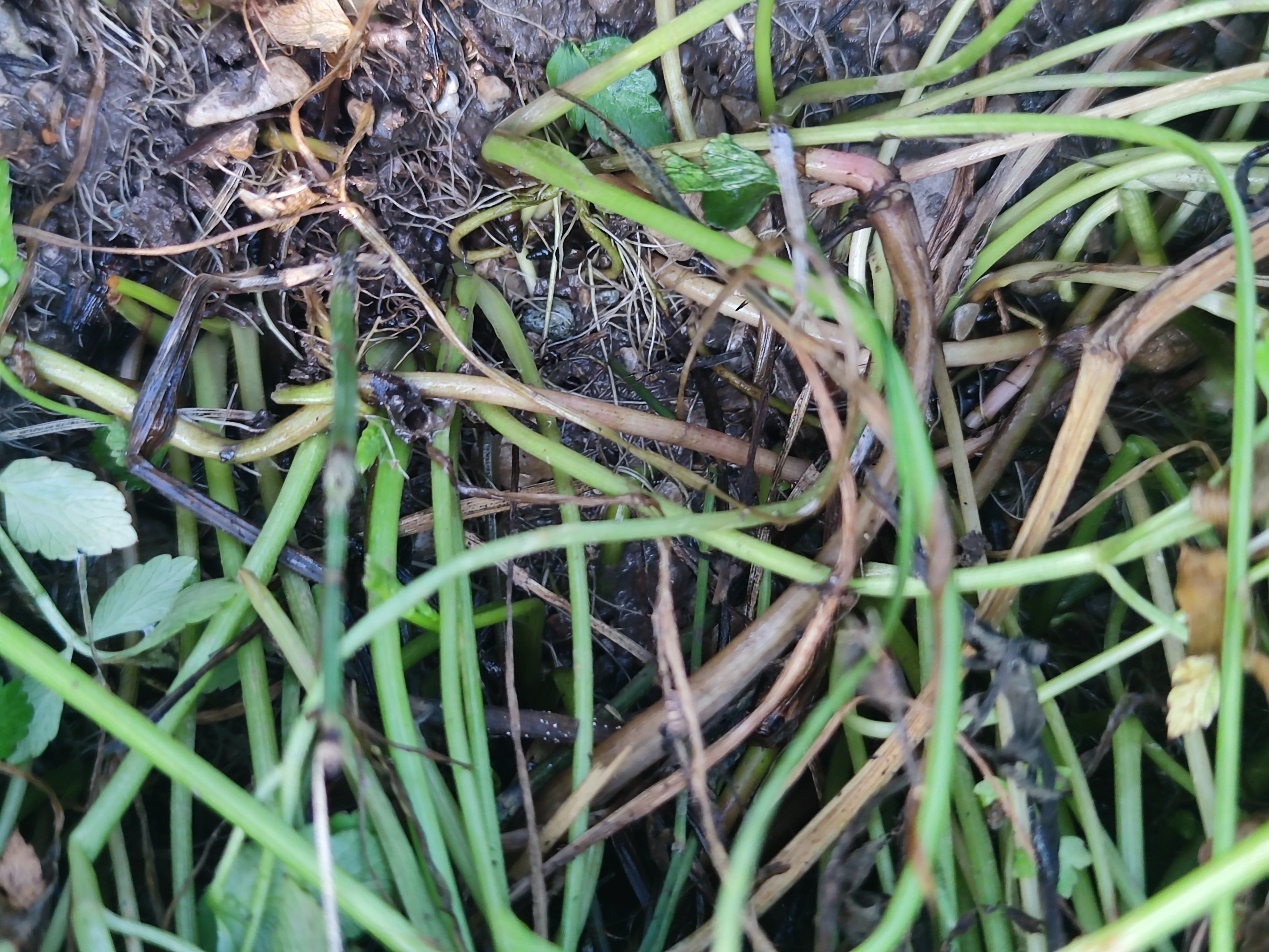


Fig F. Habitat environment of *O. hupensis* in sampling site of EY2


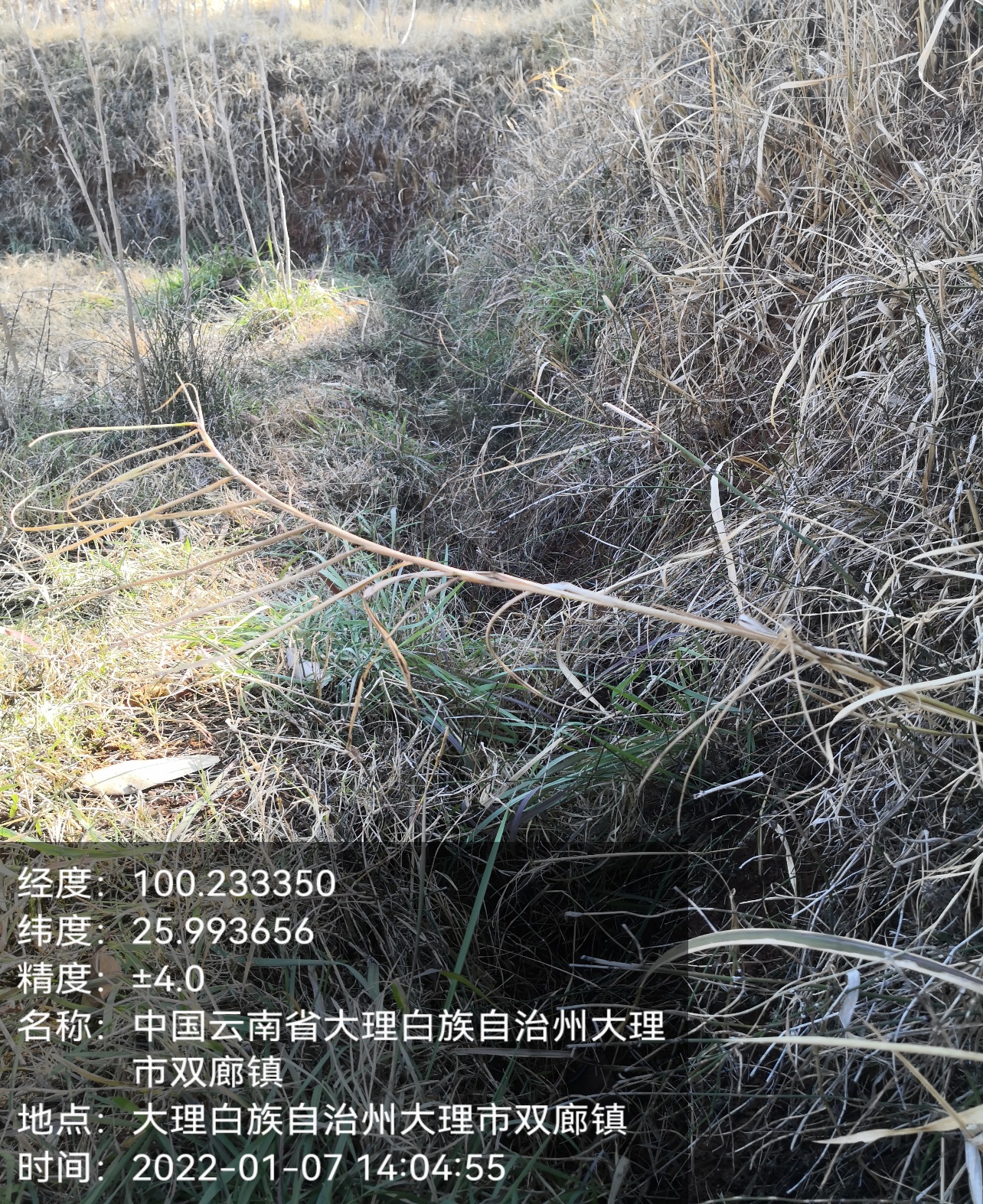

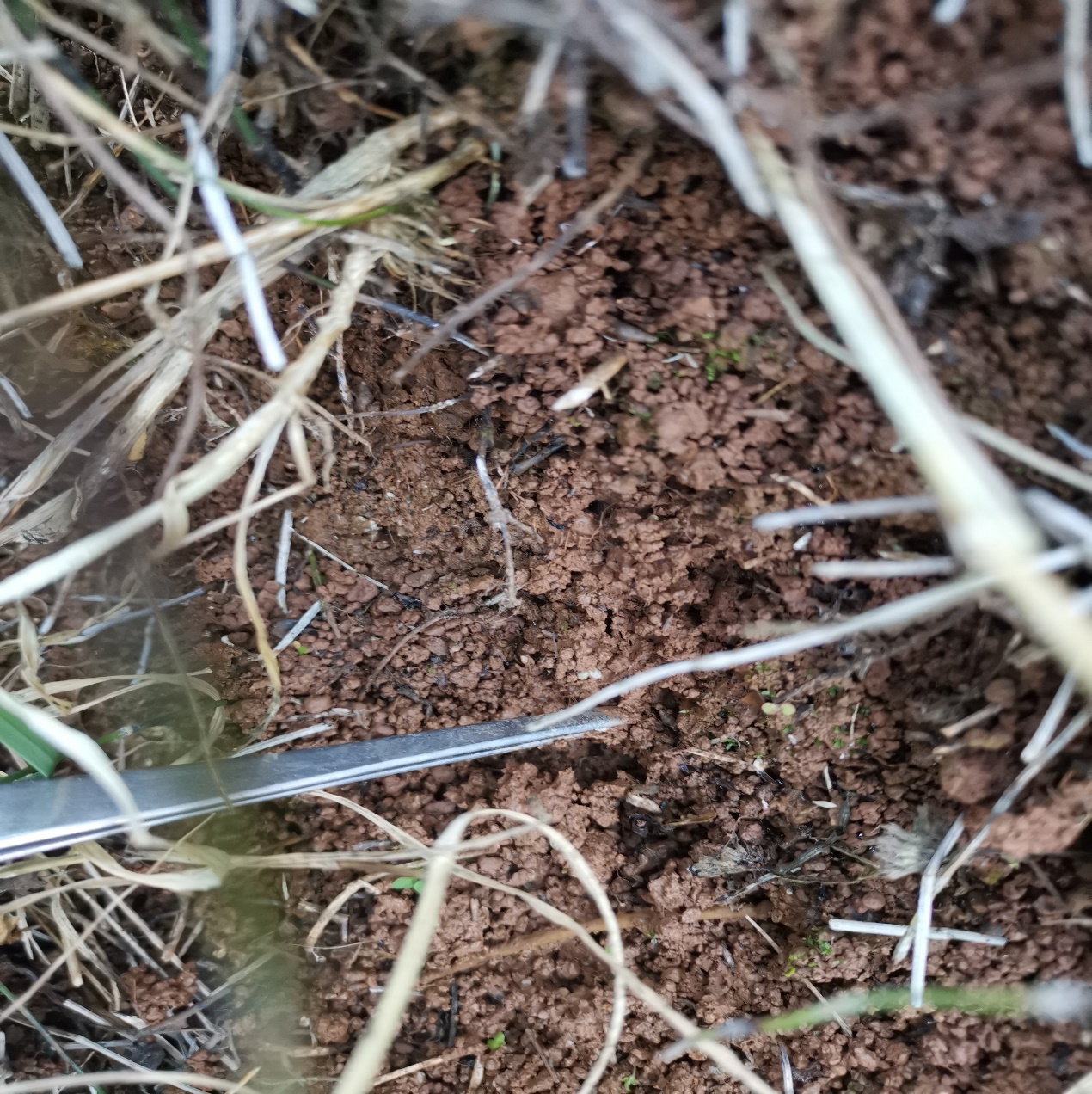


Fig G. Habitat environment of *O. hupensis* in sampling site of DL2


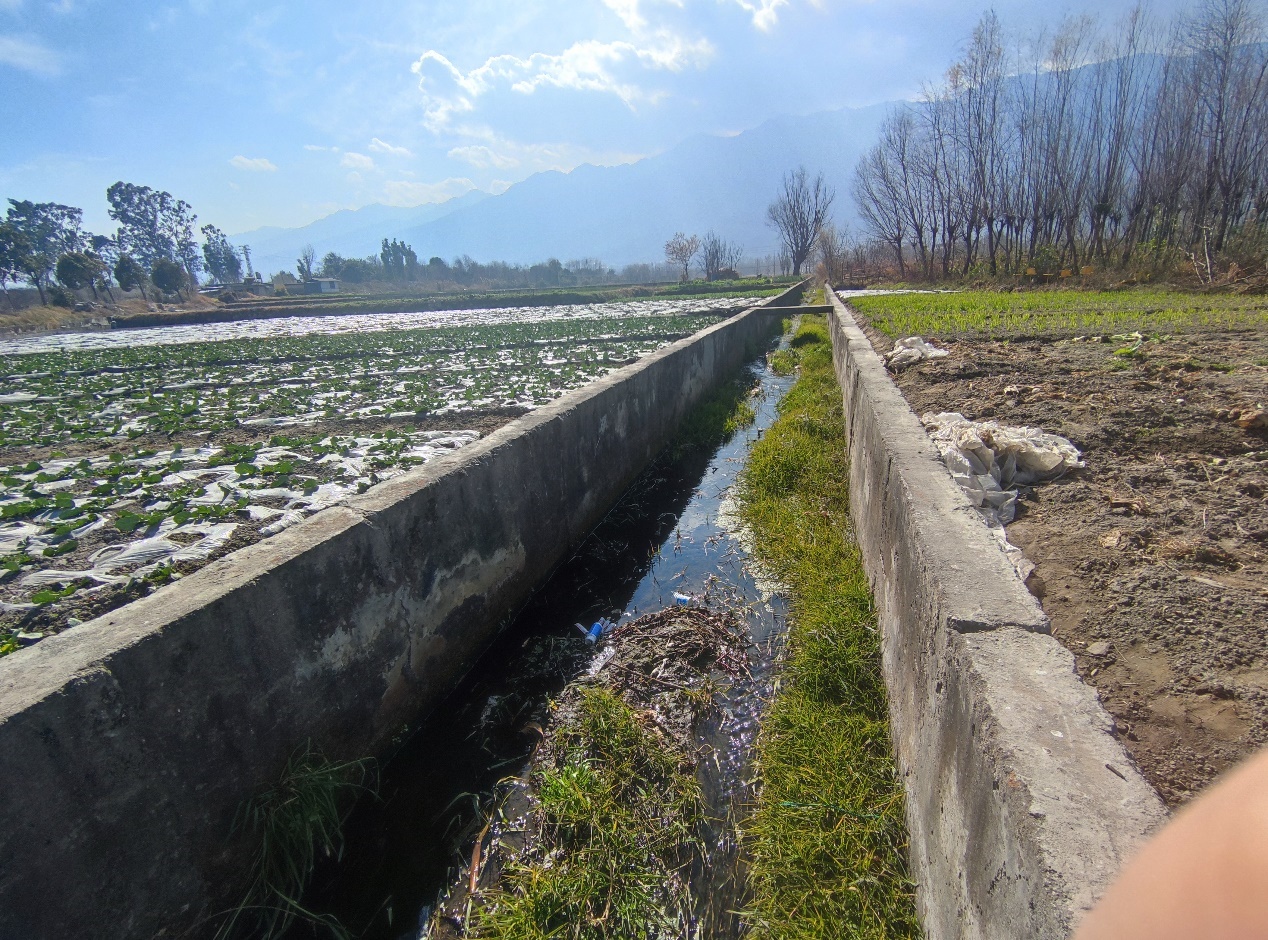

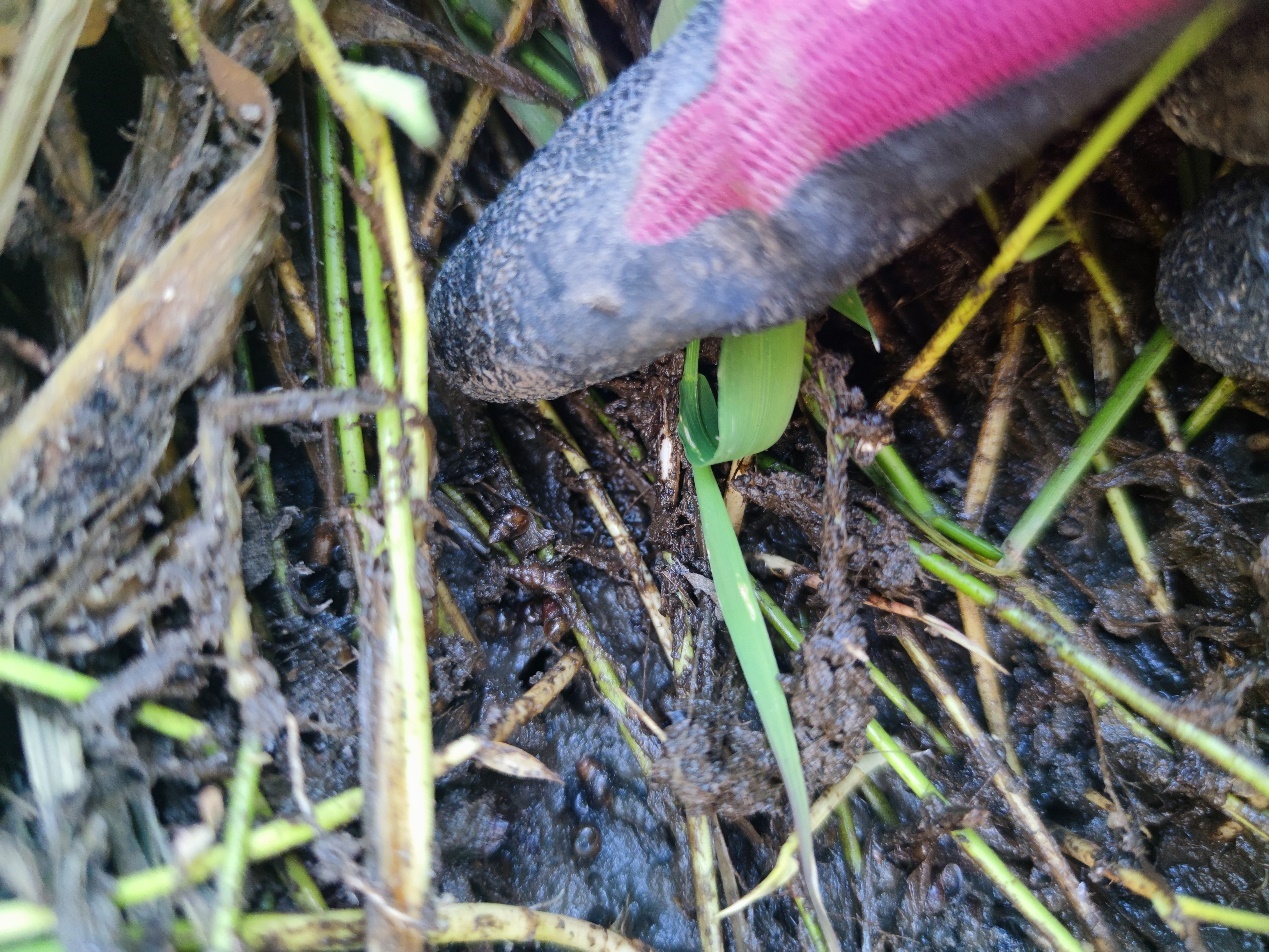


Fig H. Habitat environment of *O. hupensis* in sampling site of DL3


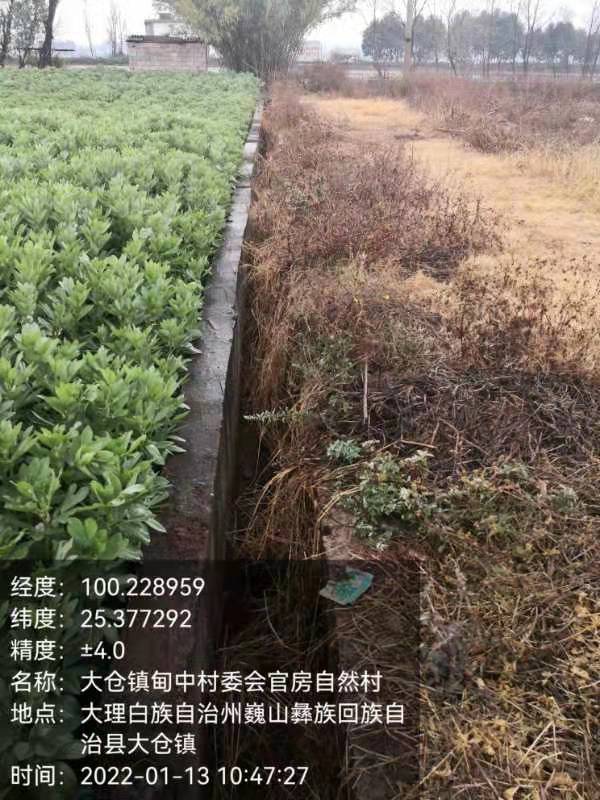

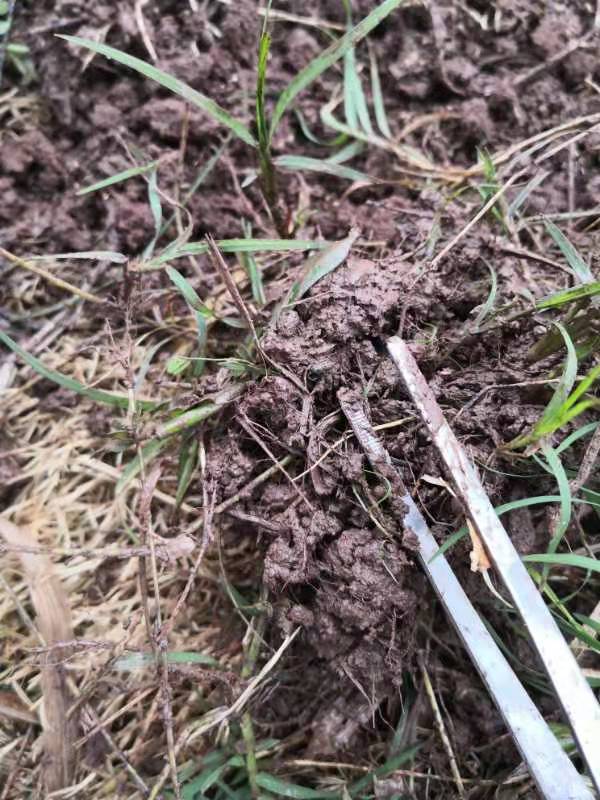


Fig I. Habitat environment of *O. hupensis* in sampling site of WS1


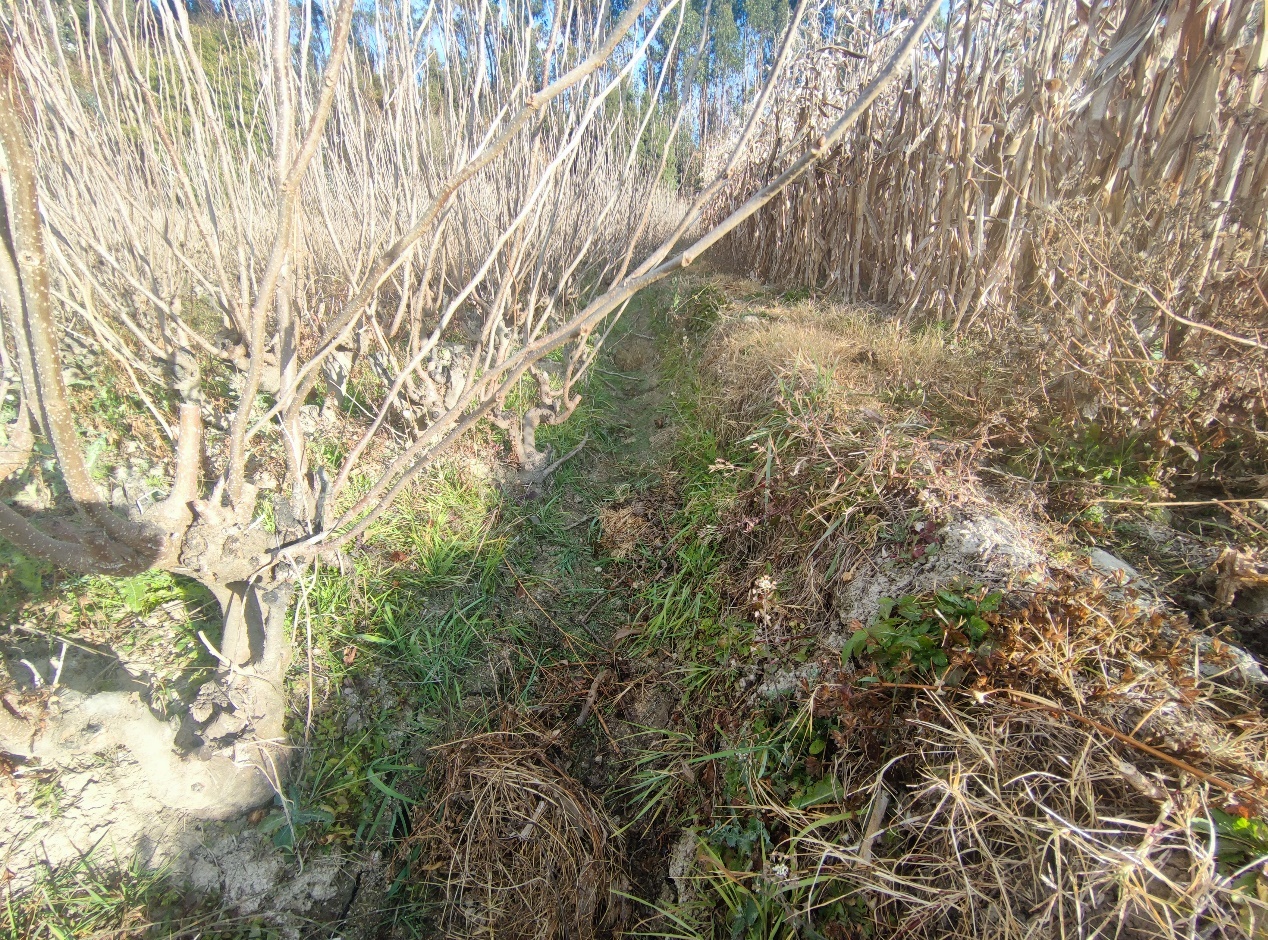

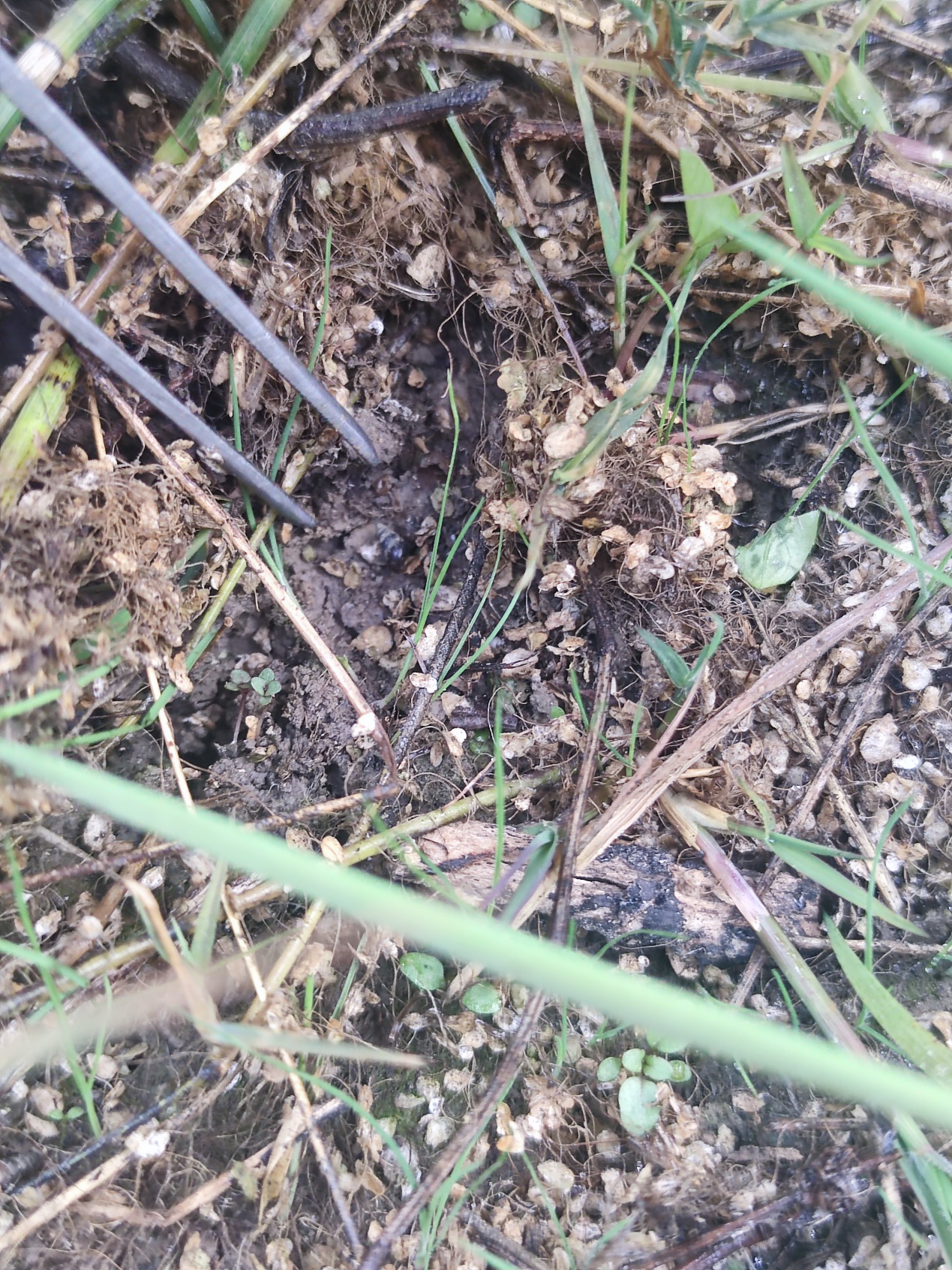


Fig J. Habitat environment of *O. hupensis* in sampling site of HQ1


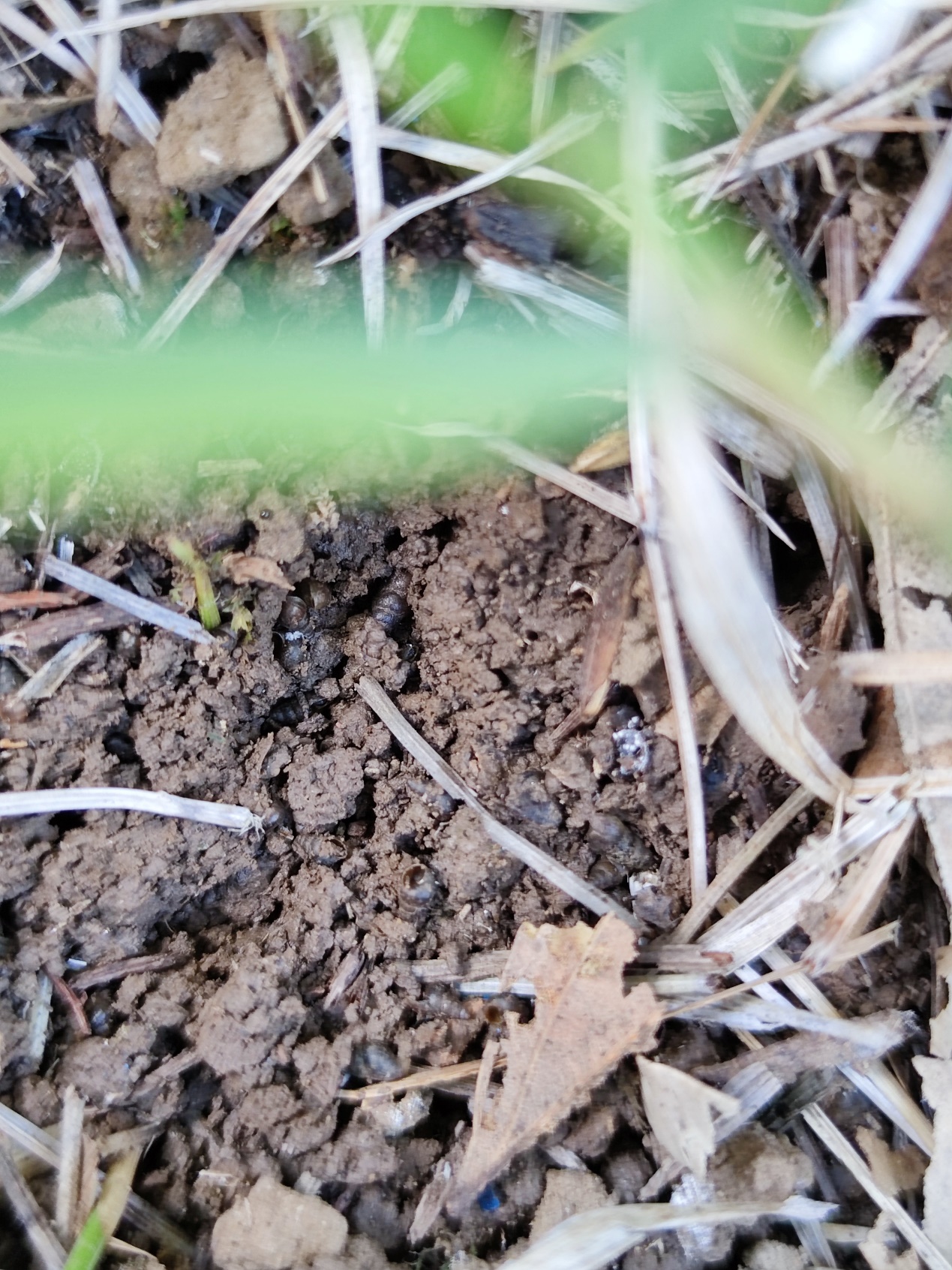


Fig K. Habitat environment of *O. hupensis* in sampling site of YS3


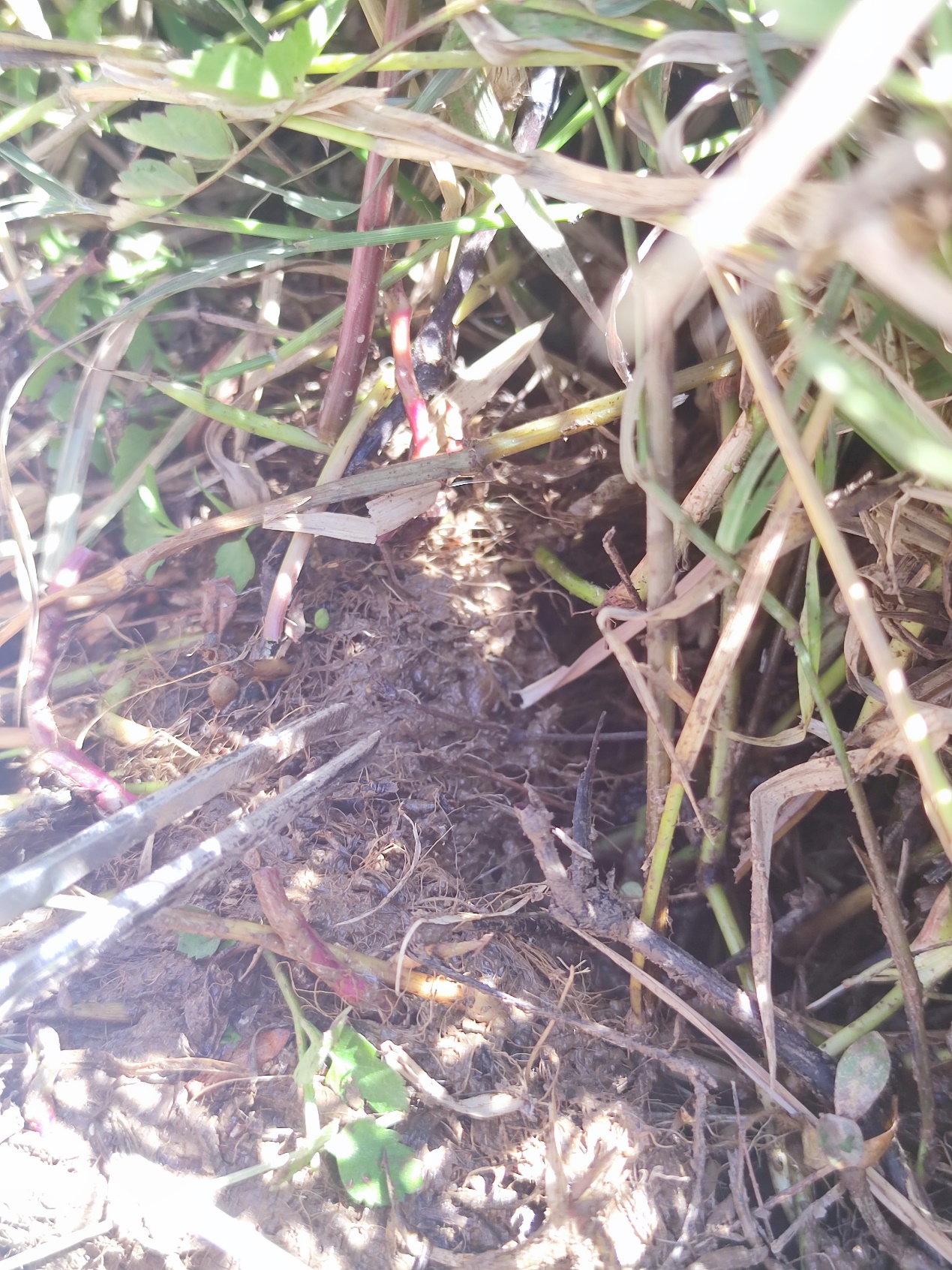


Fig L. Habitat environment of *O. hupensis* in sampling site of GC1


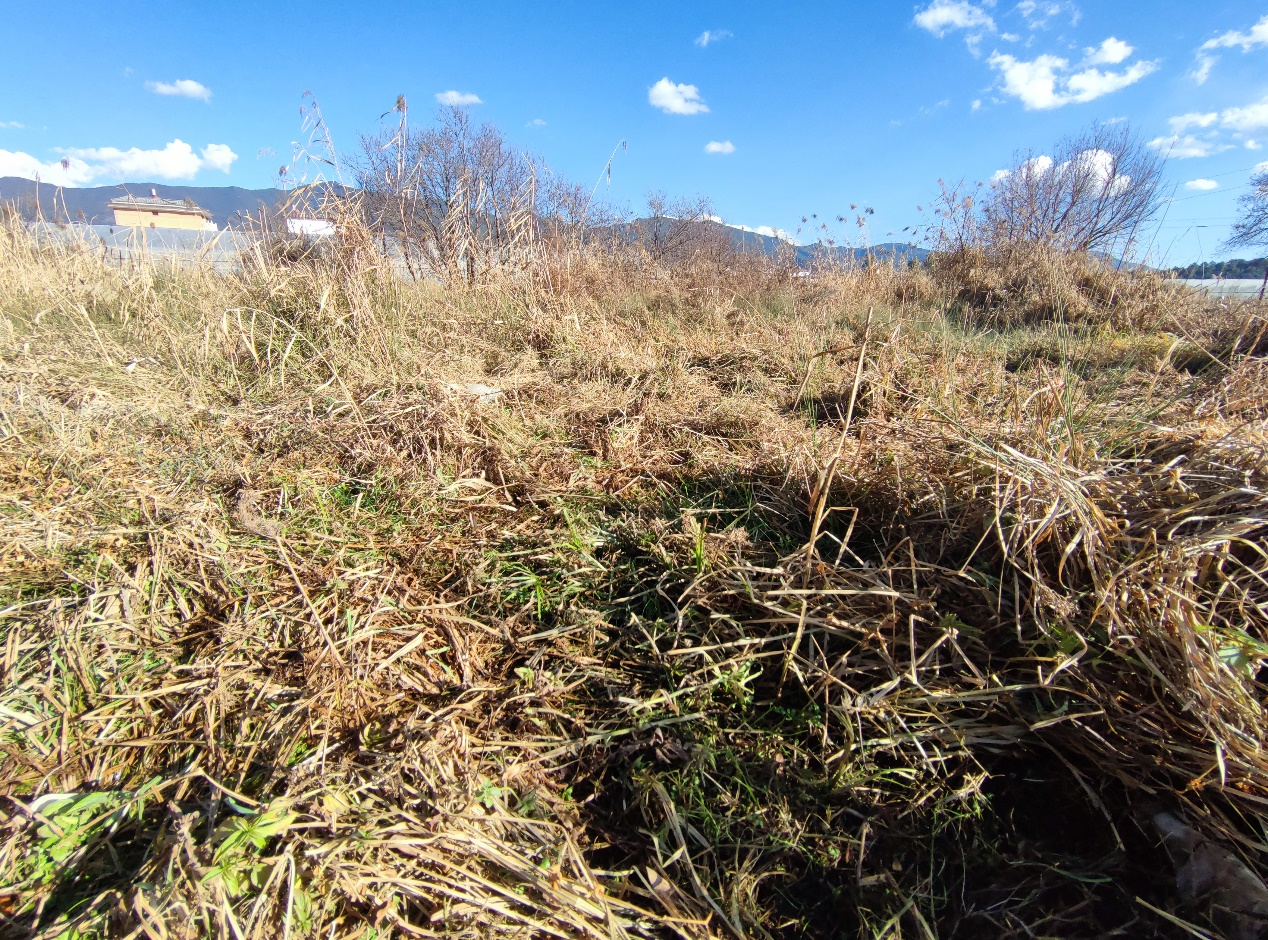


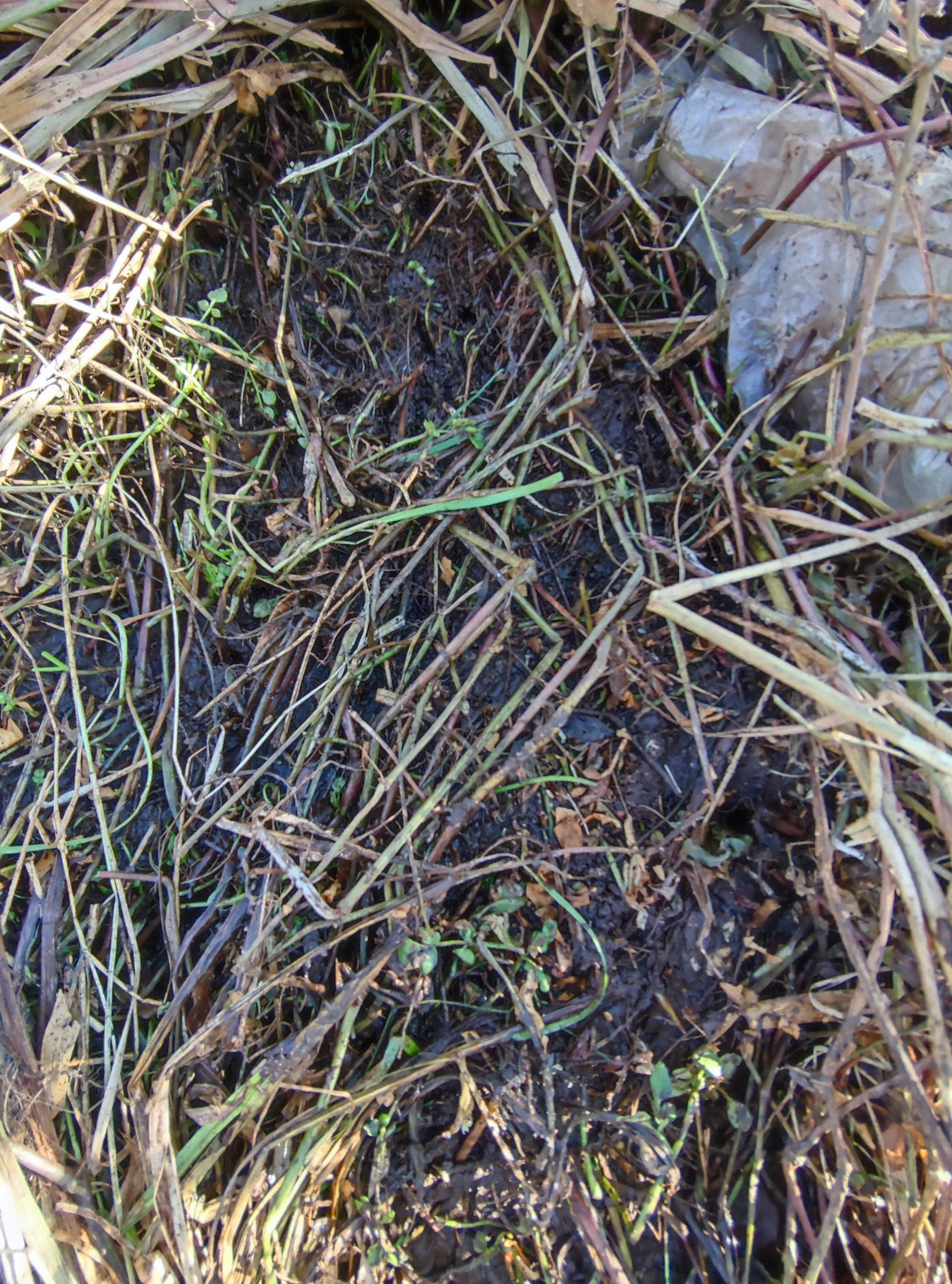


Fig M. Habitat environment of *O. hupensis* in sampling site of GC2


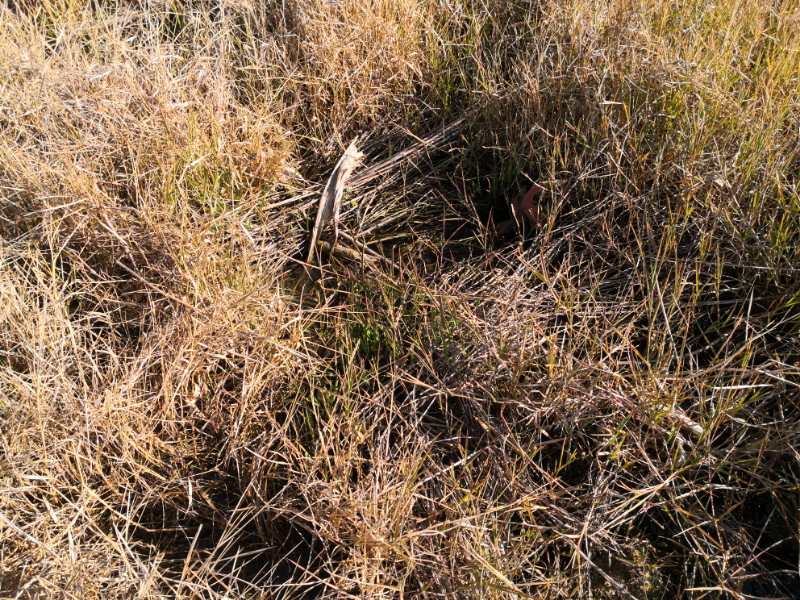


Fig N. Habitat environment of *O. hupensis* in sampling site of CX2
