# Supplementary material for: Genetic differentiation of Oncomelania hupensis robertsoni in hilly regions of China: Using the complete mitochondrial genome
Source: PLoS Negl Trop Dis. 2024 Nov 26;18(11):e0012094. doi: 10.1371/journal.pntd.0012094 (PMC11630586; doi:10.1371/journal.pntd.0012094)
Supplement: S2 File — Table A. The organization of the mitochondrial genome of O. h. r. Yunnan strain in sampling sites of Nanjian County. Table B. The organization of the mitochondrial genome of O. h. r. Yunnan strain in sampling sites of Midu County (MD1-06). Table C. The organization of the mitochondrial genome of O. h. r. Yunnan strain in sampling sites of Xiangyun County (XY2-03). Table D. The organization of the mitochondrial genome of O. h. r. Yunnan strain in sampling sites of Eryuan County. Table E. The organization of the mitochondrial genome of O. h. r. Yunnan strain in sampling sites of Dali City. Table F. The organization of the mitochondrial genome of O. h. r. Yunnan strain in sampling sites of Heqing County. Table G. The organization of the mitochondrial genome of O. h. r. Yunnan strain in sampling sites of Yongsheng County. Table H. The organization of the mitochondrial genome of O. h. r. Yunnan strain in sampling sites of Lijiang City. Table I. The organization of the mitochondrial genome of O. h. r. Yunnan strain in sampling sites of Chuxiong City. (DOCX) [file pntd.0012094.s002.docx]

Table A. The organization of the mitochondrial genome of *O. h. r.* Yunnan strain in sampling sites of Nanjian County

| Genes | Location | | Length (bp) | Start codon | Termination codon | AT content (%) | Interval (bp) | Coding strand |
| --- | --- | --- | --- | --- | --- | --- | --- | --- |
|  | Start | Stop |  |  |  |  |  |  |
| **NJ1-01** | | | | | | | | |
| COX1 | 1 | 1536 | 1536 | ATG | TAA | 62.76 | 16 | + |
| COX2 | 1553 | 2239 | 687 | ATG | TAA | 65.21 | 2 | + |
| trnD (guc) | 2242 | 2308 | 67 |  |  | 79.1 | 0 | + |
| ATP8 | 2309 | 2467 | 159 | ATG | TAA | 75.47 | 7 | + |
| ATP6 | 2475 | 3170 | 696 | ATG | TAA | 68.53 | 30 | + |
| trnM (cau) | 3201 | 3265 | 65 |  |  | 67.69 | 1 | - |
| trnY (gua) | 3267 | 3331 | 65 |  |  | 60 | 9 | - |
| trnC (gca) | 3341 | 3404 | 64 |  |  | 76.56 | 1 | - |
| trnW (uca) | 3406 | 3471 | 66 |  |  | 69.7 | 1 | - |
| trnQ (uug) | 3473 | 3534 | 62 |  |  | 62.3 | 3 | - |
| trnG (ucc) | 3538 | 3603 | 66 |  |  | 66.67 | 5 | - |
| trnE (uuc) | 3609 | 3675 | 67 |  |  | 68.66 | 0 | - |
| s-rRNA | 3676 | 4620 | 945 |  |  | 69.6 | -2 | + |
| trnV (uac) | 4619 | 4685 | 67 |  |  | 70.15 | 0 | + |
| l-rRNA | 4686 | 6030 | 1345 |  |  | 73.29 | 0 | + |
| trnL (uaa) | 6031 | 6098 | 68 |  |  | 73.53 | 0 | + |
| trnL (uag) | 6099 | 6167 | 69 |  |  | 76.81 | 0 | + |
| ND1 | 6168 | 7118 | 951 | ATG | TAG | 67.26 | -11 | + |
| trnP (ugg) | 7108 | 7174 | 67 |  |  | 64.18 | 9 | + |
| ND6 | 7184 | 7675 | 492 | ATT | TAG | 70.33 | 4 | + |
| CYTB | 7677 | 8819 | 1143 | ATT | TAG | 64.48 | 0 | + |
| trnS (uga) | 8820 | 8884 | 65 |  |  | 55.38 | 0 | + |
| trnT (ugu) | 8885 | 8950 | 66 |  |  | 72.73 | 7 | - |
| ND4L | 8958 | 9254 | 297 | ATG | TAG | 69.36 | 38 | + |
| ND4 | 9293 | 10618 | 1326 | ATA | TAA | 68.6 | 2 | + |
| trnH (gug) | 10621 | 10685 | 65 |  |  | 67.69 | 0 | + |
| ND5 | 10686 | 12398 | 1713 | ATG | TAG | 66.73 | 1 | + |
| trnF (gaa) | 12400 | 12466 | 67 |  |  | 65.67 | 68 | + |
| COX3 | 12535 | 13314 | 780 | ATG | TAA | 61.15 | 11 | + |
| trnK (uuu) | 13326 | 13393 | 68 |  |  | 73.53 | 19 | + |
| trnA (ugc) | 13413 | 13479 | 67 |  |  | 71.64 | 0 | + |
| trnR (ucg) | 13480 | 13548 | 69 |  |  | 60.87 | 17 | + |
| trnN (guu) | 13566 | 13632 | 67 |  |  | 67.16 | 11 | + |
| trnI (gau) | 13644 | 13710 | 67 |  |  | 65.67 | 0 | + |
| ND3 | 13711 | 14064 | 354 | ATG | TAA | 64.97 | 0 | + |
| trnS (gcu) | 14065 | 14132 | 68 |  |  | 58.82 | 0 | + |
| ND2 | 14133 | 15185 | 1053 | ATT | TAA | 68.92 | 1 | + |
| Table A. The organization of the mitochondrial genome of *O. h. r.* Yunnan strain in sampling sites of Nanjian County (continued) | | | | | | | | |
| **NJ2-04** | | | | | | | | |
| COX1 | 1 | 1536 | 1536 | ATG | TAG | 62.63 | 16 | + |
| COX2 | 1553 | 2239 | 687 | ATG | TAA | 65.94 | 2 | + |
| trnD (guc) | 2242 | 2308 | 67 |  |  | 79.1 | 0 | + |
| ATP8 | 2309 | 2467 | 159 | ATG | TAA | 74.21 | 7 | + |
| ATP6 | 2475 | 3170 | 696 | ATG | TAA | 67.96 | 30 | + |
| trnM (cau) | 3201 | 3265 | 65 |  |  | 64.62 | 1 | - |
| trnY (gua) | 3267 | 3331 | 65 |  |  | 60 | 9 | - |
| trnC (gca) | 3341 | 3404 | 64 |  |  | 76.56 | 1 | - |
| trnW (uca) | 3406 | 3472 | 67 |  |  | 70.15 | 1 | - |
| trnQ (uug) | 3474 | 3535 | 62 |  |  | 62.3 | 3 | - |
| trnG (ucc) | 3539 | 3604 | 66 |  |  | 63.64 | 4 | - |
| trnE (uuc) | 3609 | 3675 | 67 |  |  | 68.66 | 0 | - |
| s-rRNA | 3676 | 4619 | 944 |  |  | 69.67 | -2 | + |
| trnV (uac) | 4618 | 4684 | 67 |  |  | 71.64 | 0 | + |
| l-rRNA | 4685 | 6029 | 1345 |  |  | 72.99 | 0 | + |
| trnL (uaa) | 6030 | 6097 | 68 |  |  | 73.53 | 0 | + |
| trnL (uag) | 6098 | 6166 | 69 |  |  | 75.36 | 0 | + |
| ND1 | 6167 | 7117 | 951 | ATG | TAG | 67.47 | -11 | + |
| trnP (ugg) | 7107 | 7173 | 67 |  |  | 62.69 | 9 | + |
| ND6 | 7183 | 7674 | 492 | ATT | TAA | 69.51 | 4 | + |
| CYTB | 7676 | 8818 | 1143 | ATT | TAG | 64.83 | 0 | + |
| trnS (uga) | 8819 | 8883 | 65 |  |  | 53.85 | 0 | + |
| trnT (ugu) | 8884 | 8949 | 66 |  |  | 74.24 | 7 | - |
| ND4L | 8957 | 9253 | 297 | ATG | TAG | 70.37 | 38 | + |
| ND4 | 9292 | 10617 | 1326 | ATA | TAA | 69.36 | 2 | + |
| trnH (gug) | 10620 | 10684 | 65 |  |  | 67.69 | 0 | + |
| ND5 | 10685 | 12397 | 1713 | ATG | TAG | 66.9 | 1 | + |
| trnF (gaa) | 12399 | 12465 | 67 |  |  | 65.67 | 68 | + |
| COX3 | 12534 | 13313 | 780 | ATG | TAA | 61.03 | 11 | + |
| trnK (uuu) | 13325 | 13392 | 68 |  |  | 73.53 | 19 | + |
| trnA (ugc) | 13412 | 13478 | 67 |  |  | 73.13 | 0 | + |
| trnR (ucg) | 13479 | 13547 | 69 |  |  | 60.87 | 17 | + |
| trnN (guu) | 13565 | 13631 | 67 |  |  | 64.18 | 11 | + |
| trnI (gau) | 13643 | 13709 | 67 |  |  | 64.18 | 0 | + |
| ND3 | 13710 | 14063 | 354 | ATG | TAA | 64.41 | 0 | + |
| trnS (gcu) | 14064 | 14131 | 68 |  |  | 60.29 | 0 | + |
| ND2 | 14132 | 15184 | 1053 | ATT | TAA | 68.54 | 1 | + |
| **NJ2-09** | | | | | | | | |
| COX1 | 1 | 1536 | 1536 | ATG | TAG | 62.63 | 16 | + |
| COX2 | 1553 | 2239 | 687 | ATG | TAA | 65.94 | 2 | + |
| trnD (guc) | 2242 | 2308 | 67 |  |  | 79.1 | 0 | + |
| Table A. The organization of the mitochondrial genome of *O. h. r.* Yunnan strain in sampling sites of Nanjian County (continued) | | | | | | | | |
| ATP8 | 2309 | 2467 | 159 | ATG | TAA | 74.21 | 7 | + |
| ATP6 | 2475 | 3170 | 696 | ATG | TAA | 67.96 | 30 | + |
| trnM (cau) | 3201 | 3265 | 65 |  |  | 64.62 | 1 | - |
| trnY (gua) | 3267 | 3331 | 65 |  |  | 60 | 9 | - |
| trnC (gca) | 3341 | 3404 | 64 |  |  | 76.56 | 1 | - |
| trnW (uca) | 3406 | 3472 | 67 |  |  | 70.15 | 1 | - |
| trnQ (uug) | 3474 | 3535 | 62 |  |  | 62.3 | 3 | - |
| trnG (ucc) | 3539 | 3604 | 66 |  |  | 63.64 | 4 | - |
| trnE (uuc) | 3609 | 3675 | 67 |  |  | 68.66 | 0 | - |
| s-rRNA | 3676 | 4619 | 944 |  |  | 69.57 | -2 | + |
| trnV (uac) | 4618 | 4684 | 67 |  |  | 71.64 | 0 | + |
| l-rRNA | 4685 | 6029 | 1345 |  |  | 72.99 | 0 | + |
| trnL (uaa) | 6030 | 6097 | 68 |  |  | 73.53 | 0 | + |
| trnL (uag) | 6098 | 6166 | 69 |  |  | 75.36 | 0 | + |
| ND1 | 6167 | 7117 | 951 | ATG | TAG | 67.47 | -11 | + |
| trnP (ugg) | 7107 | 7173 | 67 |  |  | 62.69 | 9 | + |
| ND6 | 7183 | 7674 | 492 | ATT | TAA | 69.51 | 4 | + |
| CYTB | 7676 | 8818 | 1143 | ATT | TAG | 64.83 | 0 | + |
| trnS (uga) | 8819 | 8883 | 65 |  |  | 53.85 | 0 | + |
| trnT (ugu) | 8884 | 8949 | 66 |  |  | 74.24 | 7 | - |
| ND4L | 8957 | 9253 | 297 | ATG | TAG | 70.37 | 38 | + |
| ND4 | 9292 | 10617 | 1326 | ATA | TAA | 69.28 | 2 | + |
| trnH (gug) | 10620 | 10684 | 65 |  |  | 67.69 | 0 | + |
| ND5 | 10685 | 12397 | 1713 | ATG | TAG | 66.9 | 1 | + |
| trnF (gaa) | 12399 | 12465 | 67 |  |  | 65.67 | 68 | + |
| COX3 | 12534 | 13313 | 780 | ATG | TAA | 61.15 | 11 | + |
| trnK (uuu) | 13325 | 13392 | 68 |  |  | 73.53 | 19 | + |
| trnA (ugc) | 13412 | 13478 | 67 |  |  | 73.13 | 0 | + |
| trnR (ucg) | 13479 | 13547 | 69 |  |  | 60.87 | 17 | + |
| trnN (guu) | 13565 | 13631 | 67 |  |  | 64.18 | 11 | + |
| trnI (gau) | 13643 | 13709 | 67 |  |  | 64.18 | 0 | + |
| ND3 | 13710 | 14063 | 354 | ATG | TAA | 64.41 | 0 | + |
| trnS (gcu) | 14064 | 14131 | 68 |  |  | 60.29 | 0 | + |
| ND2 | 14132 | 15184 | 1053 | ATT | TAA | 68.54 | 1 | + |

Table B. The organization of the mitochondrial genome of *O. h. r.* Yunnan strain in sampling sites of Midu County (MD1-06)

| Genes | Location | | Length (bp) | Start codon | Termination codon | AT content (%) | Interval (bp) | Coding strand |
| --- | --- | --- | --- | --- | --- | --- | --- | --- |
|  | Start | Stop |  |  |  |  |  |  |
| COX1 | 1 | 1536 | 1536 | ATG | TAG | 62.83 | 16 | + |
| COX2 | 1553 | 2239 | 687 | ATG | TAA | 65.65 | 2 | + |
| trnD (guc) | 2242 | 2308 | 67 |  |  | 79.1 | 0 | + |
| ATP8 | 2309 | 2467 | 159 | ATG | TAA | 75.47 | 7 | + |
| ATP6 | 2475 | 3170 | 696 | ATG | TAA | 68.97 | 30 | + |
| trnM (cau) | 3201 | 3265 | 65 |  |  | 66.15 | 1 | - |
| trnY (gua) | 3267 | 3331 | 65 |  |  | 60 | 9 | - |
| trnC (gca) | 3341 | 3404 | 64 |  |  | 76.56 | 1 | - |
| trnW (uca) | 3406 | 3471 | 66 |  |  | 69.7 | 1 | - |
| trnQ (uug) | 3473 | 3534 | 62 |  |  | 62.3 | 3 | - |
| trnG (ucc) | 3538 | 3603 | 66 |  |  | 63.64 | 3 | - |
| trnE (uuc) | 3607 | 3673 | 67 |  |  | 68.66 | 0 | - |
| s-rRNA | 3674 | 4617 | 944 |  |  | 69.88 | -2 | + |
| trnV (uac) | 4616 | 4682 | 67 |  |  | 70.15 | 0 | + |
| l-rRNA | 4683 | 6025 | 1343 |  |  | 73.25 | 0 | + |
| trnL (uaa) | 6026 | 6093 | 68 |  |  | 73.53 | 0 | + |
| trnL (uag) | 6094 | 6162 | 69 |  |  | 75.36 | 0 | + |
| ND1 | 6163 | 7113 | 951 | ATG | TAG | 67.47 | -11 | + |
| trnP (ugg) | 7103 | 7169 | 67 |  |  | 64.18 | 9 | + |
| ND6 | 7179 | 7670 | 492 | ATT | TAA | 70.12 | 4 | + |
| CYTB | 7672 | 8814 | 1143 | ATT | TAG | 64.65 | 0 | + |
| trnS (uga) | 8815 | 8879 | 65 |  |  | 53.85 | 0 | + |
| trnT (ugu) | 8880 | 8945 | 66 |  |  | 72.73 | 7 | - |
| ND4L | 8953 | 9249 | 297 | ATG | TAG | 70.71 | 38 | + |
| ND4 | 9288 | 10613 | 1325 | ATA | TAA | 68.68 | 2 | + |
| trnH (gug) | 10616 | 10680 | 65 |  |  | 67.69 | 0 | + |
| ND5 | 10681 | 12393 | 1713 | ATG | TAG | 66.84 | 1 | + |
| trnF (gaa) | 12395 | 12461 | 67 |  |  | 67.16 | 69 | + |
| COX3 | 12531 | 13310 | 780 | ATG | TAA | 60.9 | 11 | + |
| trnK (uuu) | 13322 | 13389 | 68 |  |  | 73.53 | 19 | + |
| trnA (ugc) | 13409 | 13475 | 67 |  |  | 71.64 | 0 | + |
| trnR (ucg) | 13476 | 13544 | 69 |  |  | 60.87 | 17 | + |
| trnN (guu) | 13562 | 13628 | 67 |  |  | 67.16 | 11 | + |
| trnI (gau) | 13640 | 13706 | 67 |  |  | 65.67 | 0 | + |
| ND3 | 13740 | 14060 | 354 | ATG | TAA | 65.25 | 0 | + |
| trnS (gcu) | 14061 | 14128 | 68 |  |  | 58.82 | 0 | + |
| ND2 | 14129 | 15181 | 1053 | ATT | TAA | 69.3 | 1 | + |

Table C. The organization of the mitochondrial genome of *O. h. r.* Yunnan strain in sampling sites of Xiangyun County (XY2-03)

| Genes | Location | | Length (bp) | Start codon | Termination codon | AT content (%) | Interval (bp) | Coding strand |
| --- | --- | --- | --- | --- | --- | --- | --- | --- |
|  | Start | Stop |  |  |  |  |  |  |
| COX1 | 1 | 1536 | 1536 | ATG | TAA | 62.83 | 16 | + |
| COX2 | 1553 | 2239 | 687 | ATG | TAA | 65.21 | 2 | + |
| trnD (guc) | 2242 | 2308 | 67 |  |  | 79.1 | 0 | + |
| ATP8 | 2309 | 2467 | 159 | ATG | TAA | 75.47 | 7 | + |
| ATP6 | 2475 | 3170 | 696 | ATG | TAA | 68.68 | 30 | + |
| trnM (cau) | 3201 | 3265 | 65 |  |  | 67.69 | 1 | - |
| trnY (gua) | 3267 | 3331 | 65 |  |  | 60 | 9 | - |
| trnC (gca) | 3341 | 3404 | 64 |  |  | 76.56 | 1 | - |
| trnW (uca) | 3406 | 3471 | 66 |  |  | 69.7 | 1 | - |
| trnQ (uug) | 3473 | 3534 | 62 |  |  | 62.3 | 3 | - |
| trnG (ucc) | 3538 | 3603 | 66 |  |  | 66.67 | 5 | - |
| trnE (uuc) | 3609 | 3675 | 67 |  |  | 68.66 | 0 | - |
| s-rRNA | 3676 | 4620 | 945 |  |  | 69.7 | -2 | + |
| trnV (uac) | 4619 | 4685 | 67 |  |  | 70.15 | 0 | + |
| l-rRNA | 4686 | 6030 | 1345 |  |  | 73.21 | 0 | + |
| trnL (uaa) | 6031 | 6098 | 68 |  |  | 73.53 | 0 | + |
| trnL (uag) | 6099 | 6167 | 69 |  |  | 76.81 | 0 | + |
| ND1 | 6168 | 7118 | 951 | ATG | TAG | 67.37 | -11 | + |
| trnP (ugg) | 7108 | 7174 | 67 |  |  | 64.18 | 9 | + |
| ND6 | 7184 | 7675 | 492 | ATT | TAG | 70.73 | 4 | + |
| CYTB | 7677 | 8819 | 1143 | ATT | TAG | 64.57 | 0 | + |
| trnS (uga) | 8820 | 8884 | 65 |  |  | 52.31 | 0 | + |
| trnT (ugu) | 8885 | 8950 | 66 |  |  | 72.73 | 7 | - |
| ND4L | 8958 | 9254 | 297 | ATG | TAG | 69.36 | 38 | + |
| ND4 | 9293 | 10618 | 1326 | ATA | TAA | 68.75 | 2 | + |
| trnH (gug) | 10621 | 10685 | 65 |  |  | 67.69 | 0 | + |
| ND5 | 10686 | 12398 | 1713 | ATG | TAG | 66.73 | 1 | + |
| trnF (gaa) | 12400 | 12466 | 67 |  |  | 65.67 | 68 | + |
| COX3 | 12535 | 13314 | 780 | ATG | TAA | 61.15 | 11 | + |
| trnK (uuu) | 13326 | 13393 | 68 |  |  | 73.53 | 19 | + |
| trnA (ugc) | 13413 | 13479 | 67 |  |  | 71.64 | 0 | + |
| trnR (ucg) | 13480 | 13548 | 69 |  |  | 60.87 | 17 | + |
| trnN (guu) | 13566 | 13632 | 67 |  |  | 67.16 | 11 | + |
| trnI (gau) | 13644 | 13710 | 67 |  |  | 65.67 | 0 | + |
| ND3 | 13711 | 14064 | 354 | ATG | TAA | 64.97 | 0 | + |
| trnS (gcu) | 14065 | 14132 | 68 |  |  | 58.82 | 0 | + |
| ND2 | 14133 | 15185 | 1053 | ATT | TAA | 68.82 | 1 | + |

Table D. The organization of the mitochondrial genome of *O. h. r.* Yunnan strain in sampling sites of Eryuan County

| Genes | Location | | Length (bp) | Start codon | Termination codon | AT content (%) | Interval (bp) | Coding strand |
| --- | --- | --- | --- | --- | --- | --- | --- | --- |
|  | Start | Stop |  |  |  |  |  |  |
| **EY1-10** | | | | | | | | |
| COX1 | 1 | 1536 | 1536 | ATG | TAA | 62.76 | 16 | + |
| COX2 | 1553 | 2239 | 687 | ATG | TAA | 65.21 | 2 | + |
| trnD (guc) | 2242 | 2308 | 67 |  |  | 79.1 | 0 | + |
| ATP8 | 2309 | 2467 | 159 | ATG | TAA | 75.47 | 7 | + |
| ATP6 | 2475 | 3170 | 696 | ATG | TAA | 68.82 | 30 | + |
| trnM (cau) | 3201 | 3265 | 65 |  |  | 67.69 | 1 | - |
| trnY (gua) | 3267 | 3331 | 65 |  |  | 60 | 9 | - |
| trnC (gca) | 3341 | 3404 | 64 |  |  | 76.56 | 1 | - |
| trnW (uca) | 3406 | 3471 | 66 |  |  | 69.7 | 1 | - |
| trnQ (uug) | 3473 | 3534 | 62 |  |  | 62.3 | 3 | - |
| trnG (ucc) | 3538 | 3603 | 66 |  |  | 66.67 | 5 | - |
| trnE (uuc) | 3609 | 3675 | 67 |  |  | 68.66 | 0 | - |
| s-rRNA | 3676 | 4620 | 945 |  |  | 69.6 | -2 | + |
| trnV (uac) | 4619 | 4685 | 67 |  |  | 70.15 | 0 | + |
| l-rRNA | 4686 | 6030 | 1345 |  |  | 73.14 | 0 | + |
| trnL (uaa) | 6031 | 6098 | 68 |  |  | 73.53 | 0 | + |
| trnL (uag) | 6099 | 6167 | 69 |  |  | 76.81 | 0 | + |
| ND1 | 6168 | 7118 | 951 | ATG | TAG | 67.47 | -11 | + |
| trnP (ugg) | 7108 | 7174 | 67 |  |  | 64.18 | 9 | + |
| ND6 | 7184 | 7675 | 492 | ATT | TAG | 70.33 | 4 | + |
| CYTB | 7677 | 8819 | 1143 | ATT | TAG | 64.57 | 0 | + |
| trnS (uga) | 8820 | 8884 | 65 |  |  | 52.31 | 0 | + |
| trnT (ugu) | 8885 | 8950 | 66 |  |  | 72.73 | 7 | - |
| ND4L | 8958 | 9254 | 297 | ATG | TAG | 69.02 | 38 | + |
| ND4 | 9293 | 10618 | 1326 | ATA | TAA | 68.91 | 2 | + |
| trnH (gug) | 10621 | 10685 | 65 |  |  | 67.69 | 0 | + |
| ND5 | 10686 | 12398 | 1713 | ATG | TAG | 66.9 | 1 | + |
| trnF (gaa) | 12400 | 12466 | 67 |  |  | 65.67 | 68 | + |
| COX3 | 12535 | 13314 | 780 | ATG | TAA | 61.15 | 11 | + |
| trnK (uuu) | 13326 | 13393 | 68 |  |  | 73.53 | 19 | + |
| trnA (ugc) | 13413 | 13479 | 67 |  |  | 71.64 | 0 | + |
| trnR (ucg) | 13480 | 13548 | 69 |  |  | 60.87 | 17 | + |
| trnN (guu) | 13566 | 13632 | 67 |  |  | 67.16 | 11 | + |
| trnI (gau) | 13644 | 13710 | 67 |  |  | 65.67 | 0 | + |
| ND3 | 13711 | 14064 | 354 | ATG | TAA | 64.97 | 0 | + |
| trnS (gcu) | 14065 | 14132 | 68 |  |  | 58.82 | 0 | + |
| ND2 | 14133 | 15185 | 1053 | ATT | TAA | 68.82 | 1 | + |
| Table D. The organization of the mitochondrial genome of *O. h. r.* Yunnan strain in sampling sites of Eryuan County (continued) | | | | | | | | |
| **EY2-06** | | | | | | | | |
| COX1 | 1 | 1536 | 1536 | ATG | TAA | 62.83 | 16 | + |
| COX2 | 1553 | 2239 | 687 | ATG | TAA | 65.21 | 2 | + |
| trnD (guc) | 2242 | 2308 | 67 |  |  | 79.1 | 0 | + |
| ATP8 | 2309 | 2467 | 159 | ATG | TAA | 76.1 | 7 | + |
| ATP6 | 2475 | 3170 | 696 | ATG | TAA | 68.53 | 30 | + |
| trnM (cau) | 3201 | 3265 | 65 |  |  | 67.69 | 1 | - |
| trnY (gua) | 3267 | 3331 | 65 |  |  | 60 | 9 | - |
| trnC (gca) | 3341 | 3404 | 64 |  |  | 76.56 | 1 | - |
| trnW (uca) | 3406 | 3471 | 66 |  |  | 71.21 | 1 | - |
| trnQ (uug) | 3473 | 3534 | 62 |  |  | 62.3 | 3 | - |
| trnG (ucc) | 3538 | 3603 | 66 |  |  | 66.67 | 5 | - |
| trnE (uuc) | 3609 | 3675 | 67 |  |  | 68.66 | 0 | - |
| s-rRNA | 3676 | 4620 | 945 |  |  | 69.6 | -2 | + |
| trnV (uac) | 4619 | 4685 | 67 |  |  | 70.15 | 0 | + |
| l-rRNA | 4686 | 6030 | 1345 |  |  | 73.07 | 0 | + |
| trnL (uaa) | 6031 | 6098 | 68 |  |  | 73.53 | 0 | + |
| trnL (uag) | 6099 | 6167 | 69 |  |  | 76.81 | 0 | + |
| ND1 | 6168 | 7118 | 951 | ATG | TAG | 67.47 | -11 | + |
| trnP (ugg) | 7108 | 7174 | 67 |  |  | 64.18 | 9 | + |
| ND6 | 7184 | 7675 | 492 | ATT | TAG | 70.33 | 4 | + |
| CYTB | 7677 | 8819 | 1143 | ATT | TAG | 64.57 | 0 | + |
| trnS (uga) | 8820 | 8884 | 65 |  |  | 52.31 | 0 | + |
| trnT (ugu) | 8885 | 8950 | 66 |  |  | 72.73 | 7 | - |
| ND4L | 8958 | 9254 | 297 | ATA | TAG | 69.36 | 38 | + |
| ND4 | 9293 | 10618 | 1326 | ATA | TAA | 68.75 | 2 | + |
| trnH (gug) | 10621 | 10685 | 65 |  |  | 67.69 | 0 | + |
| ND5 | 10686 | 12398 | 1713 | ATG | TAG | 66.78 | 1 | + |
| trnF (gaa) | 12400 | 12466 | 67 |  |  | 65.67 | 68 | + |
| COX3 | 12535 | 13314 | 780 | ATG | TAA | 61.28 | 11 | + |
| trnK (uuu) | 13326 | 13393 | 68 |  |  | 73.53 | 19 | + |
| trnA (ugc) | 13413 | 13479 | 67 |  |  | 71.64 | 0 | + |
| trnR (ucg) | 13480 | 13548 | 69 |  |  | 60.87 | 17 | + |
| trnN (guu) | 13566 | 13632 | 67 |  |  | 67.16 | 11 | + |
| trnI (gau) | 13644 | 13710 | 67 |  |  | 65.67 | 0 | + |
| ND3 | 13711 | 14064 | 354 | ATG | TAA | 64.97 | 0 | + |
| trnS (gcu) | 14065 | 14132 | 68 |  |  | 58.82 | 0 | + |
| ND2 | 14133 | 15185 | 1053 | ATT | TAA | 68.92 | 1 | + |

Table E. The organization of the mitochondrial genome of *O. h. r.* Yunnan strain in sampling sites of Dali City

| Genes | Location | | Length (bp) | Start codon | Termination codon | AT content (%) | Interval (bp) | Coding strand |
| --- | --- | --- | --- | --- | --- | --- | --- | --- |
|  | Start | Stop |  |  |  |  |  |  |
| **DL2-01** | | | | | | | | |
| COX1 | 1 | 1536 | 1536 | ATG | TAA | 62.89 | 16 | + |
| COX2 | 1553 | 2239 | 687 | ATG | TAA | 65.21 | 2 | + |
| trnD (guc) | 2242 | 2308 | 67 |  |  | 79.1 | 0 | + |
| ATP8 | 2309 | 2467 | 159 | ATG | TAA | 76.1 | 7 | + |
| ATP6 | 2475 | 3170 | 696 | ATG | TAA | 68.53 | 30 | + |
| trnM (cau) | 3201 | 3265 | 65 |  |  | 67.69 | 1 | - |
| trnY (gua) | 3267 | 3331 | 65 |  |  | 60 | 9 | - |
| trnC (gca) | 3341 | 3404 | 64 |  |  | 76.56 | 1 | - |
| trnW (uca) | 3406 | 3471 | 66 |  |  | 71.21 | 1 | - |
| trnQ (uug) | 3473 | 3534 | 62 |  |  | 62.3 | 3 | - |
| trnG (ucc) | 3538 | 3603 | 66 |  |  | 66.67 | 5 | - |
| trnE (uuc) | 3609 | 3675 | 67 |  |  | 68.66 | 0 | - |
| s-rRNA | 3676 | 4620 | 945 |  |  | 69.6 | -2 | + |
| trnV (uac) | 4619 | 4685 | 67 |  |  | 70.15 | 0 | + |
| l-rRNA | 4686 | 6030 | 1345 |  |  | 73.21 | 0 | + |
| trnL (uaa) | 6031 | 6098 | 68 |  |  | 73.53 | 0 | + |
| trnL (uag) | 6099 | 6167 | 69 |  |  | 76.81 | 0 | + |
| ND1 | 6168 | 7118 | 951 | ATG | TAG | 67.47 | -11 | + |
| trnP (ugg) | 7108 | 7174 | 67 |  |  | 64.18 | 9 | + |
| ND6 | 7184 | 7675 | 492 | ATT | TAG | 70.33 | 4 | + |
| CYTB | 7677 | 8819 | 1143 | ATT | TAG | 64.57 | 0 | + |
| trnS (uga) | 8820 | 8884 | 65 |  |  | 52.31 | 0 | + |
| trnT (ugu) | 8885 | 8950 | 66 |  |  | 72.73 | 7 | - |
| ND4L | 8958 | 9254 | 297 | ATG | TAG | 69.36 | 38 | + |
| ND4 | 9293 | 10618 | 1326 | ATA | TAA | 68.75 | 2 | + |
| trnH (gug) | 10621 | 10685 | 65 |  |  | 67.69 | 0 | + |
| ND5 | 10686 | 12398 | 1713 | ATG | TAG | 66.67 | 1 | + |
| trnF (gaa) | 12400 | 12466 | 67 |  |  | 65.67 | 68 | + |
| COX3 | 12535 | 13314 | 780 | ATG | TAA | 61.28 | 11 | + |
| trnK (uuu) | 13326 | 13393 | 68 |  |  | 73.53 | 19 | + |
| trnA (ugc) | 13413 | 13479 | 67 |  |  | 71.64 | 0 | + |
| trnR (ucg) | 13480 | 13548 | 69 |  |  | 60.87 | 17 | + |
| trnN (guu) | 13566 | 13632 | 67 |  |  | 67.16 | 11 | + |
| trnI (gau) | 13644 | 13710 | 67 |  |  | 65.67 | 0 | + |
| ND3 | 13711 | 14064 | 354 | ATG | TAA | 64.97 | 0 | + |
| trnS (gcu) | 14065 | 14132 | 68 |  |  | 58.82 | 0 | + |
| ND2 | 14133 | 15185 | 1053 | ATT | TAA | 68.92 | 1 | + |
|  | | | | | | | | |
| Table E. The organization of the mitochondrial genome of *O. h. r.* Yunnan strain in sampling sites of Dali City (continued) | | | | | | | | |
| **DL2-03** | | | | | | | | |
| COX1 | 1 | 1536 | 1536 | ATG | TAA | 62.89 | 16 | + |
| COX2 | 1553 | 2239 | 687 | ATG | TAA | 65.21 | 2 | + |
| trnD (guc) | 2242 | 2308 | 67 |  |  | 79.1 | 0 | + |
| ATP8 | 2309 | 2467 | 159 | ATG | TAA | 76.1 | 7 | + |
| ATP6 | 2475 | 3170 | 696 | ATG | TAA | 68.53 | 30 | + |
| trnM (cau) | 3201 | 3265 | 65 |  |  | 67.69 | 1 | - |
| trnY (gua) | 3267 | 3331 | 65 |  |  | 60 | 9 | - |
| trnC (gca) | 3341 | 3404 | 64 |  |  | 76.56 | 1 | - |
| trnW (uca) | 3406 | 3471 | 66 |  |  | 71.21 | 1 | - |
| trnQ (uug) | 3473 | 3534 | 62 |  |  | 62.3 | 3 | - |
| trnG (ucc) | 3538 | 3603 | 66 |  |  | 66.67 | 5 | - |
| trnE (uuc) | 3609 | 3675 | 67 |  |  | 68.66 | 0 | - |
| s-rRNA | 3676 | 4620 | 945 |  |  | 69.6 | -2 | + |
| trnV (uac) | 4619 | 4685 | 67 |  |  | 70.15 | 0 | + |
| l-rRNA | 4686 | 6030 | 1345 |  |  | 73.21 | 0 | + |
| trnL (uaa) | 6031 | 6098 | 68 |  |  | 73.53 | 0 | + |
| trnL (uag) | 6099 | 6167 | 69 |  |  | 76.81 | 0 | + |
| ND1 | 6168 | 7118 | 951 | ATG | TAG | 67.47 | -11 | + |
| trnP (ugg) | 7108 | 7174 | 67 |  |  | 64.18 | 9 | + |
| ND6 | 7184 | 7675 | 492 | ATT | TAG | 70.33 | 4 | + |
| CYTB | 7677 | 8819 | 1143 | ATT | TAG | 64.57 | 0 | + |
| trnS (uga) | 8820 | 8884 | 65 |  |  | 52.31 | 0 | + |
| trnT (ugu) | 8885 | 8950 | 66 |  |  | 72.73 | 7 | - |
| ND4L | 8958 | 9254 | 297 | ATG | TAG | 69.36 | 38 | + |
| ND4 | 9293 | 10618 | 1326 | ATA | TAA | 68.75 | 2 | + |
| trnH (gug) | 10621 | 10685 | 65 |  |  | 67.69 | 0 | + |
| ND5 | 10686 | 12398 | 1713 | ATG | TAG | 66.67 | 1 | + |
| trnF (gaa) | 12400 | 12466 | 67 |  |  | 65.67 | 68 | + |
| COX3 | 12535 | 13314 | 780 | ATG | TAA | 61.41 | 11 | + |
| trnK (uuu) | 13326 | 13393 | 68 |  |  | 73.53 | 19 | + |
| trnA (ugc) | 13413 | 13479 | 67 |  |  | 71.64 | 0 | + |
| trnR (ucg) | 13480 | 13548 | 69 |  |  | 60.87 | 17 | + |
| trnN (guu) | 13566 | 13632 | 67 |  |  | 67.16 | 11 | + |
| trnI (gau) | 13644 | 13710 | 67 |  |  | 65.67 | 0 | + |
| ND3 | 13711 | 14064 | 354 | ATG | TAA | 64.97 | 0 | + |
| trnS (gcu) | 14065 | 14132 | 68 |  |  | 58.82 | 0 | + |
| ND2 | 14133 | 15185 | 1053 | ATT | TAA | 68.92 | 1 | + |
| **DL2-08** | | | | | | | | |
| COX1 | 1 | 1536 | 1536 | ATG | TAA | 62.89 | 16 | + |
| COX2 | 1553 | 2239 | 687 | ATG | TAA | 65.21 | 2 | + |
| trnD (guc) | 2242 | 2308 | 67 |  |  | 79.1 | 0 | + |
| Table E. The organization of the mitochondrial genome of *O. h. r.* Yunnan strain in sampling sites of Dali City (continued) | | | | | | | | |
| ATP8 | 2309 | 2467 | 159 | ATG | TAA | 76.1 | 7 | + |
| ATP6 | 2475 | 3170 | 696 | ATG | TAA | 68.53 | 30 | + |
| trnM (cau) | 3201 | 3265 | 65 |  |  | 67.69 | 1 | - |
| trnY (gua) | 3267 | 3331 | 65 |  |  | 60 | 9 | - |
| trnC (gca) | 3341 | 3404 | 64 |  |  | 76.56 | 1 | - |
| trnW (uca) | 3406 | 3471 | 66 |  |  | 71.21 | 1 | - |
| trnQ (uug) | 3473 | 3534 | 62 |  |  | 62.3 | 3 | - |
| trnG (ucc) | 3538 | 3603 | 66 |  |  | 66.67 | 5 | - |
| trnE (uuc) | 3609 | 3675 | 67 |  |  | 68.66 | 0 | - |
| s-rRNA | 3676 | 4620 | 945 |  |  | 69.6 | -2 | + |
| trnV (uac) | 4619 | 4685 | 67 |  |  | 70.15 | 0 | + |
| l-rRNA | 4686 | 6030 | 1345 |  |  | 73.21 | 0 | + |
| trnL (uaa) | 6031 | 6098 | 68 |  |  | 73.53 | 0 | + |
| trnL (uag) | 6099 | 6167 | 69 |  |  | 76.81 | 0 | + |
| ND1 | 6168 | 7118 | 951 | ATG | TAG | 67.47 | -11 | + |
| trnP (ugg) | 7108 | 7174 | 67 |  |  | 64.18 | 9 | + |
| ND6 | 7184 | 7675 | 492 | ATT | TAG | 70.33 | 4 | + |
| CYTB | 7677 | 8819 | 1143 | ATT | TAG | 64.57 | 0 | + |
| trnS (uga) | 8820 | 8884 | 65 |  |  | 52.31 | 0 | + |
| trnT (ugu) | 8885 | 8950 | 66 |  |  | 72.73 | 7 | - |
| ND4L | 8958 | 9254 | 297 | ATG | TAG | 69.36 | 38 | + |
| ND4 | 9293 | 10618 | 1326 | ATA | TAA | 68.75 | 2 | + |
| trnH (gug) | 10621 | 10685 | 65 |  |  | 67.69 | 0 | + |
| ND5 | 10686 | 12398 | 1713 | ATG | TAG | 66.73 | 1 | + |
| trnF (gaa) | 12400 | 12466 | 67 |  |  | 65.67 | 68 | + |
| COX3 | 12535 | 13314 | 780 | ATG | TAA | 61.54 | 11 | + |
| trnK (uuu) | 13326 | 13393 | 68 |  |  | 73.53 | 19 | + |
| trnA (ugc) | 13413 | 13479 | 67 |  |  | 71.64 | 0 | + |
| trnR (ucg) | 13480 | 13548 | 69 |  |  | 60.87 | 17 | + |
| trnN (guu) | 13566 | 13632 | 67 |  |  | 67.16 | 11 | + |
| trnI (gau) | 13644 | 13710 | 67 |  |  | 65.67 | 0 | + |
| ND3 | 13711 | 14064 | 354 | ATG | TAA | 64.97 | 0 | + |
| trnS (gcu) | 14065 | 14132 | 68 |  |  | 58.82 | 0 | + |
| ND2 | 14133 | 15185 | 1053 | ATT | TAA | 68.92 | 1 | + |
| **DL3-06** | | | | | | | | |
| COX1 | 1 | 1536 | 1536 | ATG | TAA | 62.83 | 16 | + |
| COX2 | 1553 | 2239 | 687 | ATG | TAA | 65.21 | 2 | + |
| trnD (guc) | 2242 | 2308 | 67 |  |  | 79.1 | 0 | + |
| ATP8 | 2309 | 2467 | 159 | ATG | TAA | 75.47 | 7 | + |
| ATP6 | 2475 | 3170 | 696 | ATG | TAA | 68.68 | 30 | + |
| trnM (cau) | 3201 | 3265 | 65 |  |  | 67.69 | 1 | - |
| trnY (gua) | 3267 | 3331 | 65 |  |  | 60 | 9 | - |
| Table E. The organization of the mitochondrial genome of *O. h. r.* Yunnan strain in sampling sites of Dali City (continued) | | | | | | | | |
| trnC (gca) | 3341 | 3404 | 64 |  |  | 76.56 | 1 | - |
| trnW (uca) | 3406 | 3471 | 66 |  |  | 69.7 | 1 | - |
| trnQ (uug) | 3473 | 3534 | 62 |  |  | 62.3 | 3 | - |
| trnG (ucc) | 3538 | 3603 | 66 |  |  | 66.67 | 5 | - |
| trnE (uuc) | 3609 | 3675 | 67 |  |  | 68.66 | 0 | - |
| s-rRNA | 3676 | 4620 | 945 |  |  | 69.7 | -2 | + |
| trnV (uac) | 4619 | 4685 | 67 |  |  | 70.15 | 0 | + |
| l-rRNA | 4686 | 6030 | 1345 |  |  | 73.14 | 0 | + |
| trnL (uaa) | 6031 | 6098 | 68 |  |  | 73.53 | 0 | + |
| trnL (uag) | 6099 | 6167 | 69 |  |  | 76.81 | 0 | + |
| ND1 | 6168 | 7118 | 951 | ATG | TAG | 67.37 | -11 | + |
| trnP (ugg) | 7108 | 7174 | 67 |  |  | 64.18 | 9 | + |
| ND6 | 7184 | 7675 | 492 | ATT | TAG | 70.33 | 4 | + |
| CYTB | 7677 | 8819 | 1143 | ATT | TAA | 64.57 | 0 | + |
| trnS (uga) | 8820 | 8884 | 65 |  |  | 53.12 | 0 | + |
| trnT (ugu) | 8885 | 8950 | 66 |  |  | 72.31 | 7 | - |
| ND4L | 8958 | 9254 | 297 | ATG | TAG | 69.26 | 38 | + |
| ND4 | 9293 | 10618 | 1326 | ATA | TAA | 68.75 | 2 | + |
| trnH (gug) | 10621 | 10685 | 65 |  |  | 68.75 | 0 | + |
| ND5 | 10686 | 12398 | 1713 | ATG | TAG | 66.71 | 1 | + |
| trnF (gaa) | 12400 | 12466 | 67 |  |  | 65.15 | 68 | + |
| COX3 | 12535 | 13314 | 780 | ATG | TAA | 61.1 | 11 | + |
| trnK (uuu) | 13326 | 13393 | 68 |  |  | 73.13 | 19 | + |
| trnA (ugc) | 13413 | 13479 | 67 |  |  | 72.73 | 0 | + |
| trnR (ucg) | 13480 | 13548 | 69 |  |  | 60.29 | 17 | + |
| trnN (guu) | 13566 | 13632 | 67 |  |  | 66.67 | 11 | + |
| trnI (gau) | 13644 | 13710 | 67 |  |  | 65.15 | 0 | + |
| ND3 | 13711 | 14064 | 354 | ATG | TAA | 64.87 | 0 | + |
| trnS (gcu) | 14065 | 14132 | 68 |  |  | 58.21 | 0 | + |
| ND2 | 14133 | 15185 | 1053 | ATT | TAA | 68.73 | 1 | + |
| **DL3-10** | | | | | | | | |
| COX1 | 1 | 1536 | 1536 | ATG | TAA | 62.76 | 16 | + |
| COX2 | 1553 | 2239 | 687 | ATG | TAA | 65.21 | 2 | + |
| trnD (guc) | 2242 | 2308 | 67 |  |  | 79.1 | 0 | + |
| ATP8 | 2309 | 2467 | 159 | ATG | TAA | 75.47 | 7 | + |
| ATP6 | 2475 | 3170 | 696 | ATG | TAA | 68.68 | 30 | + |
| trnM (cau) | 3201 | 3265 | 65 |  |  | 67.69 | 1 | - |
| trnY (gua) | 3267 | 3331 | 65 |  |  | 60 | 9 | - |
| trnC (gca) | 3341 | 3404 | 64 |  |  | 76.56 | 1 | - |
| trnW (uca) | 3406 | 3471 | 66 |  |  | 69.7 | 1 | - |
| trnQ (uug) | 3473 | 3534 | 62 |  |  | 62.3 | 3 | - |
| trnG (ucc) | 3538 | 3603 | 66 |  |  | 66.67 | 5 | - |
| Table E. The organization of the mitochondrial genome of *O. h. r.* Yunnan strain in sampling sites of Dali City (continued) | | | | | | | | |
| trnE (uuc) | 3609 | 3675 | 67 |  |  | 68.66 | 0 | - |
| s-rRNA | 3676 | 4620 | 945 |  |  | 69.6 | -2 | + |
| trnV (uac) | 4619 | 4685 | 67 |  |  | 71.64 | 0 | + |
| l-rRNA | 4686 | 6030 | 1345 |  |  | 73.14 | 0 | + |
| trnL (uaa) | 6031 | 6098 | 68 |  |  | 73.53 | 0 | + |
| trnL (uag) | 6099 | 6167 | 69 |  |  | 76.81 | 0 | + |
| ND1 | 6168 | 7118 | 951 | ATG | TAG | 67.37 | -11 | + |
| trnP (ugg) | 7108 | 7174 | 67 |  |  | 64.18 | 9 | + |
| ND6 | 7184 | 7675 | 492 | ATT | TAG | 70.33 | 4 | + |
| CYTB | 7677 | 8819 | 1143 | ATT | TAG | 64.57 | 0 | + |
| trnS (uga) | 8820 | 8884 | 65 |  |  | 52.31 | 0 | + |
| trnT (ugu) | 8885 | 8949 | 65 |  |  | 72.31 | 7 | - |
| ND4L | 8957 | 9253 | 297 | ATG | TAG | 69.02 | 38 | + |
| ND4 | 9292 | 10617 | 1326 | ATA | TAA | 68.75 | 2 | + |
| trnH (gug) | 10620 | 10684 | 65 |  |  | 67.69 | 0 | + |
| ND5 | 10685 | 12397 | 1713 | ATG | TAG | 66.73 | 1 | + |
| trnF (gaa) | 12399 | 12465 | 67 |  |  | 65.67 | 68 | + |
| COX3 | 12534 | 13313 | 780 | ATG | TAA | 61.15 | 11 | + |
| trnK (uuu) | 13325 | 13392 | 68 |  |  | 73.53 | 19 | + |
| trnA (ugc) | 13412 | 13478 | 67 |  |  | 71.64 | 0 | + |
| trnR (ucg) | 13479 | 13547 | 69 |  |  | 60.87 | 17 | + |
| trnN (guu) | 13565 | 13631 | 67 |  |  | 67.16 | 11 | + |
| trnI (gau) | 13643 | 13709 | 67 |  |  | 65.67 | 0 | + |
| ND3 | 13710 | 14063 | 354 | ATG | TAA | 64.97 | 0 | + |
| trnS (gcu) | 14064 | 14131 | 68 |  |  | 58.82 | 0 | + |
| ND2 | 14132 | 15184 | 1053 | ATT | TAA | 68.82 | 1 | + |
| **DL3-14** | | | | | | | | |
| COX1 | 1 | 1536 | 1536 | ATG | TAA | 62.76 | 16 | + |
| COX2 | 1553 | 2239 | 687 | ATG | TAA | 65.21 | 2 | + |
| trnD (guc) | 2242 | 2308 | 67 |  |  | 79.1 | 0 | + |
| ATP8 | 2309 | 2467 | 159 | ATG | TAA | 75.47 | 7 | + |
| ATP6 | 2475 | 3170 | 696 | ATG | TAA | 68.68 | 30 | + |
| trnM (cau) | 3201 | 3265 | 65 |  |  | 67.69 | 1 | - |
| trnY (gua) | 3267 | 3331 | 65 |  |  | 60 | 9 | - |
| trnC (gca) | 3341 | 3404 | 64 |  |  | 76.56 | 1 | - |
| trnW (uca) | 3406 | 3471 | 66 |  |  | 69.7 | 1 | - |
| trnQ (uug) | 3473 | 3534 | 62 |  |  | 62.3 | 3 | - |
| trnG (ucc) | 3538 | 3603 | 66 |  |  | 66.67 | 5 | - |
| trnE (uuc) | 3609 | 3675 | 67 |  |  | 68.66 | 0 | - |
| s-rRNA | 3676 | 4620 | 945 |  |  | 69.7 | -2 | + |
| trnV (uac) | 4619 | 4685 | 67 |  |  | 70.15 | 0 | + |
| l-rRNA | 4686 | 6030 | 1345 |  |  | 73.14 | 0 | + |
| Table E. The organization of the mitochondrial genome of *O. h. r.* Yunnan strain in sampling sites of Dali City (continued) | | | | | | | | |
| trnL (uaa) | 6031 | 6098 | 68 |  |  | 73.53 | 0 | + |
| trnL (uag) | 6099 | 6167 | 69 |  |  | 76.81 | 0 | + |
| ND1 | 6168 | 7118 | 951 | ATG | TAG | 67.37 | -11 | + |
| trnP (ugg) | 7108 | 7174 | 67 |  |  | 64.18 | 9 | + |
| ND6 | 7184 | 7675 | 492 | ATT | TAG | 70.33 | 4 | + |
| CYTB | 7677 | 8819 | 1143 | ATT | TAG | 64.57 | 0 | + |
| trnS (uga) | 8820 | 8884 | 65 |  |  | 52.31 | 0 | + |
| trnT (ugu) | 8885 | 8950 | 66 |  |  | 72.73 | 7 | - |
| ND4L | 8958 | 9254 | 297 | ATG | TAG | 69.36 | 38 | + |
| ND4 | 9293 | 10618 | 1327 | ATA | TAA | 68.75 | 2 | + |
| trnH (gug) | 10621 | 10685 | 65 |  |  | 67.69 | 0 | + |
| ND5 | 10686 | 12398 | 1713 | ATG | TAG | 66.73 | 1 | + |
| trnF (gaa) | 12400 | 12466 | 67 |  |  | 65.67 | 68 | + |
| COX3 | 12535 | 13314 | 780 | ATG | TAA | 61.15 | 11 | + |
| trnK (uuu) | 13326 | 13393 | 68 |  |  | 73.53 | 19 | + |
| trnA (ugc) | 13413 | 13479 | 67 |  |  | 71.64 | 0 | + |
| trnR (ucg) | 13480 | 13548 | 69 |  |  | 60.87 | 17 | + |
| trnN (guu) | 13566 | 13632 | 67 |  |  | 67.16 | 11 | + |
| trnI (gau) | 13644 | 13710 | 67 |  |  | 65.67 | 0 | + |
| ND3 | 13711 | 14064 | 354 | ATG | TAA | 64.97 | 0 | + |
| trnS (gcu) | 14065 | 14132 | 68 |  |  | 58.82 | 0 | + |
| ND2 | 14133 | 15185 | 1053 | ATT | TAA | 68.73 | 1 | + |

Table F. The organization of the mitochondrial genome of *O. h. r.* Yunnan strain in sampling sites of Heqing County

| Genes | Location | | Length (bp) | Start codon | Termination codon | AT content (%) | Interval (bp) | Coding strand |
| --- | --- | --- | --- | --- | --- | --- | --- | --- |
|  | Start | Stop |  |  |  |  |  |  |
| **HQ1-03** | | | | | | | | |
| COX1 | 1 | 1536 | 1536 | ATG | TAG | 62.24 | 16 | + |
| COX2 | 1553 | 2239 | 687 | ATG | TAA | 65.21 | 2 | + |
| trnD (guc) | 2242 | 2308 | 67 |  |  | 79.1 | 0 | + |
| ATP8 | 2309 | 2467 | 159 | ATG | TAA | 74.84 | 7 | + |
| ATP6 | 2475 | 3170 | 696 | ATG | TAA | 68.82 | 30 | + |
| trnM (cau) | 3201 | 3265 | 65 |  |  | 67.69 | 1 | - |
| trnY (gua) | 3267 | 3331 | 65 |  |  | 60 | 9 | - |
| trnC (gca) | 3341 | 3404 | 64 |  |  | 76.56 | 1 | - |
| trnW (uca) | 3406 | 3471 | 66 |  |  | 69.7 | 1 | - |
| trnQ (uug) | 3473 | 3534 | 62 |  |  | 63.93 | 3 | - |
| trnG (ucc) | 3538 | 3603 | 66 |  |  | 63.64 | 3 | - |
| trnE (uuc) | 3607 | 3673 | 67 |  |  | 68.66 | 0 | - |
| s-rRNA | 3674 | 4617 | 944 |  |  | 69.67 | -2 | + |
| trnV (uac) | 4616 | 4682 | 67 |  |  | 68.66 | 0 | + |
| l-rRNA | 4683 | 6026 | 1344 |  |  | 73.27 | 0 | + |
| trnL (uaa) | 6027 | 6094 | 68 |  |  | 73.53 | 0 | + |
| trnL (uag) | 6095 | 6163 | 69 |  |  | 75.36 | 0 | + |
| ND1 | 6164 | 7114 | 951 | ATG | TAG | 67.05 | -11 | + |
| trnP (ugg) | 7104 | 7170 | 67 |  |  | 62.69 | 9 | + |
| ND6 | 7180 | 7671 | 492 | ATT | TAA | 69.72 | 4 | + |
| CYTB | 7673 | 8815 | 1143 | ATT | TAG | 64.65 | 0 | + |
| trnS (uga) | 8816 | 8880 | 65 |  |  | 53.85 | 0 | + |
| trnT (ugu) | 8881 | 8946 | 66 |  |  | 71.21 | 7 | - |
| ND4L | 8954 | 9250 | 297 | ATG | TAG | 70.37 | 38 | + |
| ND4 | 9289 | 10614 | 1325 | ATA | TAA | 69.06 | 2 | + |
| trnH (gug) | 10617 | 10681 | 65 |  |  | 67.69 | 0 | + |
| ND5 | 10682 | 12394 | 1713 | ATG | TAA | 66.61 | 1 | + |
| trnF (gaa) | 12396 | 12462 | 67 |  |  | 67.16 | 68 | + |
| COX3 | 12531 | 13310 | 780 | ATG | TAA | 60.38 | 11 | + |
| trnK (uuu) | 13322 | 13389 | 68 |  |  | 72.06 | 19 | + |
| trnA (ugc) | 13409 | 13475 | 67 |  |  | 71.64 | 0 | + |
| trnR (ucg) | 13476 | 13544 | 69 |  |  | 59.42 | 17 | + |
| trnN (guu) | 13562 | 13628 | 67 |  |  | 65.67 | 11 | + |
| trnI (gau) | 13640 | 13706 | 67 |  |  | 65.67 | 0 | + |
| ND3 | 13707 | 14060 | 354 | ATG | TAG | 63.56 | 0 | + |
| trnS (gcu) | 14061 | 14128 | 68 |  |  | 58.82 | 0 | + |
| ND2 | 14129 | 15181 | 1053 | ATT | TAA | 68.54 | 1 | + |
| Table F. The organization of the mitochondrial genome of *O. h. r.* Yunnan strain in sampling sites of Heqing County (continued) | | | | | | | | |
| **HQ1-06** | | | | | | | | |
| COX1 | 1 | 1536 | 1536 | ATG | TAG | 62.24 | 16 | + |
| COX2 | 1553 | 2239 | 687 | ATG | TAA | 65.21 | 2 | + |
| trnD (guc) | 2242 | 2308 | 67 |  |  | 79.1 | 0 | + |
| ATP8 | 2309 | 2467 | 159 | ATG | TAA | 74.84 | 7 | + |
| ATP6 | 2475 | 3170 | 696 | ATG | TAA | 68.82 | 30 | + |
| trnM (cau) | 3201 | 3265 | 65 |  |  | 67.69 | 1 | - |
| trnY (gua) | 3267 | 3331 | 65 |  |  | 60 | 9 | - |
| trnC (gca) | 3341 | 3404 | 64 |  |  | 76.56 | 1 | - |
| trnW (uca) | 3406 | 3471 | 66 |  |  | 69.7 | 1 | - |
| trnQ (uug) | 3473 | 3534 | 62 |  |  | 62.3 | 3 | - |
| trnG (ucc) | 3538 | 3603 | 66 |  |  | 63.64 | 3 | - |
| trnE (uuc) | 3607 | 3673 | 67 |  |  | 68.66 | 0 | - |
| s-rRNA | 3674 | 4617 | 944 |  |  | 69.67 | -2 | + |
| trnV (uac) | 4616 | 4682 | 67 |  |  | 68.66 | 0 | + |
| l-rRNA | 4683 | 6026 | 1344 |  |  | 73.27 | 0 | + |
| trnL (uaa) | 6027 | 6094 | 68 |  |  | 73.53 | 0 | + |
| trnL (uag) | 6095 | 6163 | 69 |  |  | 75.36 | 0 | + |
| ND1 | 6164 | 7114 | 951 | ATG | TAG | 67.05 | -11 | + |
| trnP (ugg) | 7104 | 7170 | 67 |  |  | 62.69 | 9 | + |
| ND6 | 7180 | 7671 | 492 | ATT | TAA | 69.72 | 4 | + |
| CYTB | 7673 | 8815 | 1143 | ATT | TAG | 64.65 | 0 | + |
| trnS (uga) | 8816 | 8880 | 65 |  |  | 53.85 | 0 | + |
| trnT (ugu) | 8881 | 8946 | 66 |  |  | 71.21 | 7 | - |
| ND4L | 8954 | 9250 | 297 | ATG | TAG | 70.37 | 38 | + |
| ND4 | 9289 | 10614 | 1326 | ATA | TAA | 69.06 | 2 | + |
| trnH (gug) | 10617 | 10681 | 65 |  |  | 67.69 | 0 | + |
| ND5 | 10682 | 12394 | 1713 | ATG | TAA | 66.61 | 1 | + |
| trnF (gaa) | 12396 | 12462 | 67 |  |  | 67.16 | 68 | + |
| COX3 | 12531 | 13310 | 780 | ATG | TAA | 60.38 | 11 | + |
| trnK (uuu) | 13322 | 13389 | 68 |  |  | 72.06 | 19 | + |
| trnA (ugc) | 13409 | 13475 | 67 |  |  | 71.64 | 0 | + |
| trnR (ucg) | 13476 | 13544 | 69 |  |  | 59.42 | 17 | + |
| trnN (guu) | 13562 | 13628 | 67 |  |  | 65.67 | 11 | + |
| trnI (gau) | 13640 | 13706 | 67 |  |  | 65.67 | 0 | + |
| ND3 | 13707 | 14060 | 354 | ATG | TAG | 63.56 | 0 | + |
| trnS (gcu) | 14061 | 14128 | 68 |  |  | 58.82 | 0 | + |
| ND2 | 14129 | 15181 | 1053 | ATT | TAA | 68.54 | 1 | + |

Table G. The organization of the mitochondrial genome of *O. h. r.* Yunnan strain in sampling sites of Yongsheng County

| Genes | Location | | Length (bp) | Start codon | Termination codon | AT content (%) | Interval (bp) | Coding strand |
| --- | --- | --- | --- | --- | --- | --- | --- | --- |
|  | Start | Stop |  |  |  |  |  |  |
| **YS3-01** | | | | | | | | |
| COX1 | 1 | 1536 | 1536 | ATG | TAG | 62.24 | 16 | + |
| COX2 | 1553 | 2239 | 687 | ATG | TAA | 65.07 | 2 | + |
| trnD (guc) | 2242 | 2308 | 67 |  |  | 79.1 | 0 | + |
| ATP8 | 2309 | 2467 | 159 | ATG | TAA | 74.84 | 7 | + |
| ATP6 | 2475 | 3170 | 696 | ATG | TAA | 68.68 | 30 | + |
| trnM (cau) | 3201 | 3265 | 65 |  |  | 67.69 | 1 | - |
| trnY (gua) | 3267 | 3331 | 65 |  |  | 60 | 9 | - |
| trnC (gca) | 3341 | 3404 | 64 |  |  | 76.56 | 1 | - |
| trnW (uca) | 3406 | 3471 | 66 |  |  | 69.7 | 1 | - |
| trnQ (uug) | 3473 | 3534 | 62 |  |  | 62.3 | 3 | - |
| trnG (ucc) | 3538 | 3603 | 66 |  |  | 63.64 | 3 | - |
| trnE (uuc) | 3607 | 3673 | 67 |  |  | 68.66 | 0 | - |
| s-rRNA | 3674 | 4617 | 944 |  |  | 69.57 | -2 | + |
| trnV (uac) | 4616 | 4682 | 67 |  |  | 68.66 | 0 | + |
| l-rRNA | 4683 | 6026 | 1344 |  |  | 73.27 | 0 | + |
| trnL (uaa) | 6027 | 6094 | 68 |  |  | 73.53 | 0 | + |
| trnL (uag) | 6095 | 6163 | 69 |  |  | 75.36 | 0 | + |
| ND1 | 6164 | 7114 | 951 | ATG | TAG | 67.16 | -11 | + |
| trnP (ugg) | 7104 | 7170 | 67 |  |  | 62.69 | 9 | + |
| ND6 | 7180 | 7671 | 492 | ATT | TAA | 69.92 | 4 | + |
| CYTB | 7673 | 8815 | 1143 | ATT | TAG | 64.74 | 0 | + |
| trnS (uga) | 8816 | 8880 | 65 |  |  | 53.85 | 0 | + |
| trnT (ugu) | 8881 | 8946 | 66 |  |  | 71.21 | 7 | - |
| ND4L | 8954 | 9250 | 297 | ATG | TAG | 70.71 | 38 | + |
| ND4 | 9289 | 10614 | 1326 | ATA | TAA | 68.91 | 2 | + |
| trnH (gug) | 10617 | 10681 | 65 |  |  | 67.69 | 0 | + |
| ND5 | 10682 | 12394 | 1713 | ATG | TAA | 66.61 | 1 | + |
| trnF (gaa) | 12396 | 12462 | 67 |  |  | 67.16 | 68 | + |
| COX3 | 12531 | 13310 | 780 | ATG | TAA | 60.38 | 11 | + |
| trnK (uuu) | 13322 | 13389 | 68 |  |  | 72.06 | 19 | + |
| trnA (ugc) | 13409 | 13475 | 67 |  |  | 71.64 | 0 | + |
| trnR (ucg) | 13476 | 13544 | 69 |  |  | 59.42 | 17 | + |
| trnN (guu) | 13562 | 13628 | 67 |  |  | 65.67 | 11 | + |
| trnI (gau) | 13640 | 13706 | 67 |  |  | 67.16 | 0 | + |
| ND3 | 13707 | 14060 | 354 | ATG | TAG | 63.56 | 0 | + |
| trnS (gcu) | 14061 | 14128 | 68 |  |  | 58.82 | 0 | + |
| ND2 | 14129 | 15181 | 1053 | ATT | TAA | 68.63 | 1 | + |
| Table G. The organization of the mitochondrial genome of *O. h. r.* Yunnan strain in sampling sites of Yongsheng County (continued) | | | | | | | | |
| **YS3-02** | | | | | | | | |
| COX1 | 1 | 1536 | 1536 | ATG | TAG | 61.91 | 16 | + |
| COX2 | 1553 | 2239 | 687 | ATG | TAA | 65.07 | 2 | + |
| trnD (guc) | 2242 | 2308 | 67 |  |  | 79.1 | 0 | + |
| ATP8 | 2309 | 2467 | 159 | ATG | TAA | 74.84 | 7 | + |
| ATP6 | 2475 | 3170 | 696 | ATG | TAA | 68.97 | 30 | + |
| trnM (cau) | 3201 | 3265 | 65 |  |  | 67.69 | 1 | - |
| trnY (gua) | 3267 | 3331 | 65 |  |  | 60 | 9 | - |
| trnC (gca) | 3341 | 3404 | 64 |  |  | 76.56 | 1 | - |
| trnW (uca) | 3406 | 3471 | 66 |  |  | 69.7 | 1 | - |
| trnQ (uug) | 3473 | 3534 | 62 |  |  | 60.66 | 3 | - |
| trnG (ucc) | 3538 | 3603 | 66 |  |  | 62.12 | 5 | - |
| trnE (uuc) | 3609 | 3675 | 67 |  |  | 70.15 | 0 | - |
| s-rRNA | 3676 | 4619 | 944 |  |  | 69.88 | -2 | + |
| trnV (uac) | 4618 | 4684 | 67 |  |  | 70.15 | 0 | + |
| l-rRNA | 4685 | 6029 | 1345 |  |  | 73.36 | 0 | + |
| trnL (uaa) | 6030 | 6097 | 68 |  |  | 73.53 | 0 | + |
| trnL (uag) | 6098 | 6166 | 69 |  |  | 75.36 | 0 | + |
| ND1 | 6167 | 7117 | 951 | ATG | TAG | 67.26 | -11 | + |
| trnP (ugg) | 7107 | 7173 | 67 |  |  | 62.69 | 9 | + |
| ND6 | 7183 | 7674 | 492 | ATT | TAA | 70.12 | 4 | + |
| CYTB | 7676 | 8818 | 1143 | ATT | TAG | 64.57 | 0 | + |
| trnS (uga) | 8819 | 8883 | 65 |  |  | 55.38 | 0 | + |
| trnT (ugu) | 8884 | 8949 | 66 |  |  | 72.73 | 7 | - |
| ND4L | 8957 | 9253 | 297 | ATG | TAG | 70.71 | 38 | + |
| ND4 | 9292 | 10617 | 1326 | ATA | TAA | 69.13 | 2 | + |
| trnH (gug) | 10620 | 10684 | 65 |  |  | 67.69 | 0 | + |
| ND5 | 10685 | 12397 | 1713 | ATG | TAG | 66.78 | 1 | + |
| trnF (gaa) | 12399 | 12465 | 67 |  |  | 65.67 | 125 | + |
| COX3 | 12591 | 13313 | 723 | ATG | TAA | 58.92 | 11 | + |
| trnK (uuu) | 13325 | 13392 | 68 |  |  | 73.53 | 19 | + |
| trnA (ugc) | 13412 | 13478 | 67 |  |  | 70.15 | 0 | + |
| trnR (ucg) | 13479 | 13547 | 69 |  |  | 59.42 | 17 | + |
| trnN (guu) | 13565 | 13631 | 67 |  |  | 65.67 | 11 | + |
| trnI (gau) | 13643 | 13709 | 67 |  |  | 65.67 | 0 | + |
| ND3 | 13710 | 14063 | 354 | ATG | TAA | 64.69 | 0 | + |
| trnS (gcu) | 14064 | 14131 | 68 |  |  | 58.82 | 0 | + |
| ND2 | 14132 | 15184 | 1053 | ATT | TAA | 68.54 | 1 | + |
| **YS3-08** | | | | | | | | |
| COX1 | 1 | 1536 | 1536 | ATG | TAG | 61.91 | 16 | + |
| COX2 | 1553 | 2239 | 687 | ATG | TAA | 65.07 | 2 | + |
| trnD (guc) | 2242 | 2308 | 67 |  |  | 79.1 | 0 | + |
| Table G. The organization of the mitochondrial genome of *O. h. r.* Yunnan strain in sampling sites of Yongsheng County (continued) | | | | | | | | |
| ATP8 | 2309 | 2467 | 159 | ATG | TAA | 74.84 | 7 | + |
| ATP6 | 2475 | 3170 | 696 | ATG | TAA | 68.97 | 30 | + |
| trnM (cau) | 3201 | 3265 | 65 |  |  | 67.69 | 1 | - |
| trnY (gua) | 3267 | 3331 | 65 |  |  | 60 | 9 | - |
| trnC (gca) | 3341 | 3404 | 64 |  |  | 76.56 | 1 | - |
| trnW (uca) | 3406 | 3471 | 66 |  |  | 69.7 | 1 | - |
| trnQ (uug) | 3473 | 3534 | 62 |  |  | 60.66 | 3 | - |
| trnG (ucc) | 3538 | 3603 | 66 |  |  | 62.12 | 5 | - |
| trnE (uuc) | 3609 | 3675 | 67 |  |  | 70.15 | 0 | - |
| s-rRNA | 3676 | 4619 | 944 |  |  | 69.88 | -2 | + |
| trnV (uac) | 4618 | 4684 | 67 |  |  | 70.15 | 0 | + |
| l-rRNA | 4685 | 6029 | 1345 |  |  | 73.36 | 0 | + |
| trnL (uaa) | 6030 | 6097 | 68 |  |  | 73.53 | 0 | + |
| trnL (uag) | 6098 | 6166 | 69 |  |  | 75.36 | 0 | + |
| ND1 | 6167 | 7117 | 951 | ATG | TAG | 67.26 | -11 | + |
| trnP (ugg) | 7107 | 7173 | 67 |  |  | 62.69 | 9 | + |
| ND6 | 7183 | 7674 | 492 | ATT | TAA | 70.12 | 4 | + |
| CYTB | 7676 | 8818 | 1143 | ATT | TAG | 64.57 | 0 | + |
| trnS (uga) | 8819 | 8883 | 65 |  |  | 55.38 | 0 | + |
| trnT (ugu) | 8884 | 8950 | 67 |  |  | 71.64 | 7 | - |
| ND4L | 8958 | 9254 | 297 | ATG | TAG | 70.71 | 38 | + |
| ND4 | 9293 | 10618 | 1326 | ATA | TAA | 69.13 | 2 | + |
| trnH (gug) | 10621 | 10685 | 65 |  |  | 67.69 | 0 | + |
| ND5 | 10686 | 12398 | 1713 | ATG | TAG | 66.78 | 1 | + |
| trnF (gaa) | 12400 | 12466 | 67 |  |  | 65.67 | 68 | + |
| COX3 | 12535 | 13314 | 780 | ATG | TAA | 59.74 | 11 | + |
| trnK (uuu) | 13326 | 13393 | 68 |  |  | 73.53 | 19 | + |
| trnA (ugc) | 13413 | 13479 | 67 |  |  | 70.15 | 0 | + |
| trnR (ucg) | 13480 | 13548 | 69 |  |  | 59.42 | 17 | + |
| trnN (guu) | 13566 | 13632 | 67 |  |  | 65.67 | 11 | + |
| trnI (gau) | 13644 | 13710 | 67 |  |  | 65.67 | 0 | + |
| ND3 | 13711 | 14064 | 354 | ATG | TAA | 64.69 | 0 | + |
| trnS (gcu) | 14065 | 14132 | 68 |  |  | 58.82 | 0 | + |
| ND2 | 14133 | 15185 | 1053 | ATT | TAA | 68.54 | 1 | + |
| **YS3-10** | | | | | | | | |
| COX1 | 1 | 1536 | 1536 | ATG | TAG | 62.3 | 16 | + |
| COX2 | 1553 | 2239 | 687 | ATG | TAA | 65.21 | 2 | + |
| trnD (guc) | 2242 | 2308 | 67 |  |  | 79.1 | 0 | + |
| ATP8 | 2309 | 2467 | 159 | ATG | TAA | 74.84 | 7 | + |
| ATP6 | 2475 | 3170 | 696 | ATG | TAA | 68.97 | 30 | + |
| trnM (cau) | 3201 | 3265 | 65 |  |  | 66.15 | 1 | - |
| trnY (gua) | 3267 | 3331 | 65 |  |  | 60 | 9 | - |
| Table G. The organization of the mitochondrial genome of *O. h. r.* Yunnan strain in sampling sites of Yongsheng County (continued) | | | | | | | | |
| trnC (gca) | 3341 | 3404 | 64 |  |  | 76.56 | 1 | - |
| trnW (uca) | 3406 | 3471 | 66 |  |  | 69.7 | 1 | - |
| trnQ (uug) | 3473 | 3534 | 62 |  |  | 62.3 | 3 | - |
| trnG (ucc) | 3538 | 3603 | 66 |  |  | 63.64 | 3 | - |
| trnE (uuc) | 3607 | 3673 | 67 |  |  | 68.66 | 0 | - |
| s-rRNA | 3674 | 4617 | 944 |  |  | 69.57 | -2 | + |
| trnV (uac) | 4616 | 4682 | 67 |  |  | 70.15 | 0 | + |
| l-rRNA | 4683 | 6026 | 1344 |  |  | 73.27 | 0 | + |
| trnL (uaa) | 6027 | 6094 | 68 |  |  | 73.53 | 0 | + |
| trnL (uag) | 6095 | 6163 | 69 |  |  | 75.36 | 0 | + |
| ND1 | 6164 | 7114 | 951 | ATG | TAG | 67.05 | -11 | + |
| trnP (ugg) | 7104 | 7170 | 67 |  |  | 62.69 | 9 | + |
| ND6 | 7180 | 7671 | 492 | ATT | TAA | 69.51 | 4 | + |
| CYTB | 7673 | 8815 | 1143 | ATT | TAG | 64.74 | 0 | + |
| trnS (uga) | 8816 | 8880 | 65 |  |  | 53.85 | 0 | + |
| trnT (ugu) | 8881 | 8946 | 66 |  |  | 71.21 | 7 | - |
| ND4L | 8954 | 9250 | 297 | ATG | TAG | 70.71 | 38 | + |
| ND4 | 9289 | 10614 | 1326 | ATA | TAA | 68.91 | 2 | + |
| trnH (gug) | 10617 | 10681 | 65 |  |  | 67.69 | 0 | + |
| ND5 | 10682 | 12394 | 1713 | ATG | TAA | 66.73 | 1 | + |
| trnF (gaa) | 12396 | 12462 | 67 |  |  | 67.16 | 68 | + |
| COX3 | 12531 | 13310 | 780 | ATG | TAA | 60.77 | 11 | + |
| trnK (uuu) | 13322 | 13389 | 68 |  |  | 72.06 | 19 | + |
| trnA (ugc) | 13409 | 13475 | 67 |  |  | 71.64 | 0 | + |
| trnR (ucg) | 13476 | 13544 | 69 |  |  | 59.42 | 17 | + |
| trnN (guu) | 13562 | 13628 | 67 |  |  | 65.67 | 11 | + |
| trnI (gau) | 13640 | 13706 | 67 |  |  | 65.67 | 0 | + |
| ND3 | 13707 | 14060 | 354 | ATG | TAG | 63.56 | 0 | + |
| trnS (gcu) | 14061 | 14128 | 68 |  |  | 58.82 | 0 | + |
| ND2 | 14129 | 15181 | 1053 | ATT | TAA | 68.63 | 1 | + |

Table H. The organization of the mitochondrial genome of *O. h. r.* Yunnan strain in sampling sites of Lijiang City

| Genes | Location | | Length (bp) | Start codon | Termination codon | AT content (%) | Interval (bp) | Coding strand |
| --- | --- | --- | --- | --- | --- | --- | --- | --- |
|  | Start | Stop |  |  |  |  |  |  |
| **GC1-01** | | | | | | | | |
| COX1 | 1 | 1536 | 1536 | ATG | TAG | 62.24 | 16 | + |
| COX2 | 1553 | 2239 | 687 | ATG | TAA | 65.21 | 2 | + |
| trnD (guc) | 2242 | 2308 | 67 |  |  | 79.1 | 0 | + |
| ATP8 | 2309 | 2467 | 159 | ATG | TAA | 74.84 | 7 | + |
| ATP6 | 2475 | 3170 | 696 | ATG | TAA | 68.82 | 30 | + |
| trnM (cau) | 3201 | 3265 | 65 |  |  | 67.69 | 1 | - |
| trnY (gua) | 3267 | 3331 | 65 |  |  | 60 | 9 | - |
| trnC (gca) | 3341 | 3404 | 64 |  |  | 76.56 | 1 | - |
| trnW (uca) | 3406 | 3471 | 66 |  |  | 69.7 | 1 | - |
| trnQ (uug) | 3473 | 3534 | 62 |  |  | 62.3 | 3 | - |
| trnG (ucc) | 3538 | 3603 | 66 |  |  | 63.64 | 3 | - |
| trnE (uuc) | 3607 | 3673 | 67 |  |  | 68.66 | 0 | - |
| s-rRNA | 3674 | 4617 | 944 |  |  | 69.67 | -2 | + |
| trnV (uac) | 4616 | 4682 | 67 |  |  | 68.66 | 0 | + |
| l-rRNA | 4683 | 6026 | 1344 |  |  | 73.27 | 0 | + |
| trnL (uaa) | 6027 | 6094 | 68 |  |  | 73.53 | 0 | + |
| trnL (uag) | 6095 | 6163 | 69 |  |  | 75.36 | 0 | + |
| ND1 | 6164 | 7114 | 951 | ATG | TAG | 67.05 | -11 | + |
| trnP (ugg) | 7104 | 7170 | 67 |  |  | 62.69 | 9 | + |
| ND6 | 7180 | 7671 | 492 | ATT | TAA | 69.72 | 4 | + |
| CYTB | 7673 | 8815 | 1143 | ATT | TAG | 64.65 | 0 | + |
| trnS (uga) | 8816 | 8880 | 65 |  |  | 53.85 | 0 | + |
| trnT (ugu) | 8881 | 8946 | 66 |  |  | 71.21 | 7 | - |
| ND4L | 8954 | 9250 | 297 | ATG | TAG | 70.37 | 38 | + |
| ND4 | 9289 | 10614 | 1325 | ATA | TAA | 69.13 | 2 | + |
| trnH (gug) | 10617 | 10681 | 65 |  |  | 67.69 | 0 | + |
| ND5 | 10682 | 12394 | 1713 | ATG | TAA | 66.61 | 1 | + |
| trnF (gaa) | 12396 | 12462 | 67 |  |  | 67.16 | 68 | + |
| COX3 | 12531 | 13310 | 780 | ATG | TAA | 60.38 | 11 | + |
| trnK (uuu) | 13322 | 13389 | 68 |  |  | 72.06 | 19 | + |
| trnA (ugc) | 13409 | 13475 | 67 |  |  | 71.64 | 0 | + |
| trnR (ucg) | 13476 | 13544 | 69 |  |  | 59.42 | 17 | + |
| trnN (guu) | 13562 | 13628 | 67 |  |  | 65.67 | 11 | + |
| trnI (gau) | 13640 | 13706 | 67 |  |  | 65.67 | 0 | + |
| ND3 | 13707 | 14060 | 354 | ATG | TAG | 63.56 | 0 | + |
| trnS (gcu) | 14061 | 14128 | 68 |  |  | 58.82 | 0 | + |
| ND2 | 14129 | 15181 | 1053 | ATT | TAA | 68.73 | 1 | + |
| Table H. The organization of the mitochondrial genome of *O. h. r.* Yunnan strain in sampling sites of Lijiang City (continued) | | | | | | | | |
| **GC1-06** | | | | | | | | |
| COX1 | 1 | 1536 | 1536 | ATG | TAG | 62.24 | 16 | + |
| COX2 | 1553 | 2239 | 687 | ATG | TAA | 65.07 | 2 | + |
| trnD (guc) | 2242 | 2308 | 67 |  |  | 79.1 | 0 | + |
| ATP8 | 2309 | 2467 | 159 | ATG | TAA | 74.84 | 7 | + |
| ATP6 | 2475 | 3170 | 696 | ATG | TAA | 68.82 | 30 | + |
| trnM (cau) | 3201 | 3265 | 65 |  |  | 67.69 | 1 | - |
| trnY (gua) | 3267 | 3331 | 65 |  |  | 60 | 9 | - |
| trnC (gca) | 3341 | 3404 | 64 |  |  | 76.56 | 1 | - |
| trnW (uca) | 3406 | 3471 | 66 |  |  | 69.7 | 1 | - |
| trnQ (uug) | 3473 | 3534 | 62 |  |  | 62.3 | 3 | - |
| trnG (ucc) | 3538 | 3603 | 66 |  |  | 63.64 | 3 | - |
| trnE (uuc) | 3607 | 3673 | 67 |  |  | 68.66 | 0 | - |
| s-rRNA | 3674 | 4617 | 944 |  |  | 69.67 | -2 | + |
| trnV (uac) | 4616 | 4682 | 67 |  |  | 68.66 | 0 | + |
| l-rRNA | 4683 | 6026 | 1344 |  |  | 73.27 | 0 | + |
| trnL (uaa) | 6027 | 6094 | 68 |  |  | 73.53 | 0 | + |
| trnL (uag) | 6095 | 6163 | 69 |  |  | 75.36 | 0 | + |
| ND1 | 6164 | 7114 | 951 | ATG | TAG | 67.05 | -11 | + |
| trnP (ugg) | 7104 | 7170 | 67 |  |  | 62.69 | 9 | + |
| ND6 | 7180 | 7671 | 492 | ATT | TAA | 69.72 | 4 | + |
| CYTB | 7673 | 8815 | 1143 | ATT | TAG | 64.65 | 0 | + |
| trnS (uga) | 8816 | 8880 | 65 |  |  | 53.85 | 0 | + |
| trnT (ugu) | 8881 | 8946 | 66 |  |  | 71.21 | 7 | - |
| ND4L | 8954 | 9250 | 297 | ATG | TAG | 70.37 | 38 | + |
| ND4 | 9289 | 10614 | 1325 | ATA | TAA | 69.06 | 2 | + |
| trnH (gug) | 10617 | 10681 | 65 |  |  | 67.69 | 0 | + |
| ND5 | 10682 | 12394 | 1713 | ATG | TAA | 66.61 | 1 | + |
| trnF (gaa) | 12396 | 12462 | 67 |  |  | 67.16 | 68 | + |
| COX3 | 12531 | 13310 | 780 | ATG | TAA | 60.38 | 11 | + |
| trnK (uuu) | 13322 | 13389 | 68 |  |  | 72.06 | 19 | + |
| trnA (ugc) | 13409 | 13475 | 67 |  |  | 71.64 | 0 | + |
| trnR (ucg) | 13476 | 13544 | 69 |  |  | 59.42 | 17 | + |
| trnN (guu) | 13562 | 13628 | 67 |  |  | 65.67 | 11 | + |
| trnI (gau) | 13640 | 13706 | 67 |  |  | 65.67 | 0 | + |
| ND3 | 13707 | 14060 | 354 | ATG | TAG | 63.56 | 0 | + |
| trnS (gcu) | 14061 | 14128 | 68 |  |  | 58.82 | 0 | + |
| ND2 | 14129 | 15181 | 1053 | ATT | TAA | 68.63 | 1 | + |
| **GC2-09** | | | | | | | | |
| COX1 | 1 | 1536 | 1536 | ATG | TAG | 62.24 | 16 | + |
| COX2 | 1553 | 2239 | 687 | ATG | TAA | 65.21 | 2 | + |
| trnD (guc) | 2242 | 2308 | 67 |  |  | 79.1 | 0 | + |
| Table H. The organization of the mitochondrial genome of *O. h. r.* Yunnan strain in sampling sites of Lijiang City (continued) | | | | | | | | |
| ATP8 | 2309 | 2467 | 159 | ATG | TAA | 74.84 | 7 | + |
| ATP6 | 2475 | 3170 | 696 | ATG | TAA | 68.97 | 30 | + |
| trnM (cau) | 3201 | 3265 | 65 |  |  | 67.69 | 1 | - |
| trnY (gua) | 3267 | 3331 | 65 |  |  | 60 | 9 | - |
| trnC (gca) | 3341 | 3404 | 64 |  |  | 76.56 | 1 | - |
| trnW (uca) | 3406 | 3471 | 66 |  |  | 69.7 | 1 | - |
| trnQ (uug) | 3473 | 3534 | 62 |  |  | 62.3 | 3 | - |
| trnG (ucc) | 3538 | 3603 | 66 |  |  | 63.64 | 3 | - |
| trnE (uuc) | 3607 | 3673 | 67 |  |  | 68.66 | 0 | - |
| s-rRNA | 3674 | 4617 | 944 |  |  | 69.57 | -2 | + |
| trnV (uac) | 4616 | 4682 | 67 |  |  | 70.15 | 0 | + |
| l-rRNA | 4683 | 6026 | 1344 |  |  | 73.27 | 0 | + |
| trnL (uaa) | 6027 | 6094 | 68 |  |  | 73.53 | 0 | + |
| trnL (uag) | 6095 | 6163 | 69 |  |  | 75.36 | 0 | + |
| ND1 | 6164 | 7114 | 951 | ATG | TAG | 67.05 | -11 | + |
| trnP (ugg) | 7104 | 7170 | 67 |  |  | 62.69 | 9 | + |
| ND6 | 7180 | 7671 | 492 | ATT | TAA | 69.51 | 4 | + |
| CYTB | 7673 | 8815 | 1143 | ATT | TAG | 64.74 | 0 | + |
| trnS (uga) | 8816 | 8880 | 65 |  |  | 53.85 | 0 | + |
| trnT (ugu) | 8881 | 8946 | 66 |  |  | 71.21 | 7 | - |
| ND4L | 8954 | 9250 | 297 | ATG | TAG | 70.71 | 38 | + |
| ND4 | 9289 | 10614 | 1326 | ATA | TAA | 68.91 | 2 | + |
| trnH (gug) | 10617 | 10681 | 65 |  |  | 67.69 | 0 | + |
| ND5 | 10682 | 12394 | 1713 | ATG | TAA | 66.73 | 1 | + |
| trnF (gaa) | 12396 | 12462 | 67 |  |  | 67.16 | 68 | + |
| COX3 | 12531 | 13310 | 780 | ATG | TAA | 60.64 | 11 | + |
| trnK (uuu) | 13322 | 13389 | 68 |  |  | 72.06 | 19 | + |
| trnA (ugc) | 13409 | 13475 | 67 |  |  | 71.64 | 0 | + |
| trnR (ucg) | 13476 | 13544 | 69 |  |  | 59.42 | 17 | + |
| trnN (guu) | 13562 | 13628 | 67 |  |  | 65.67 | 11 | + |
| trnI (gau) | 13640 | 13706 | 67 |  |  | 65.67 | 0 | + |
| ND3 | 13707 | 14060 | 354 | ATG | TAG | 63.84 | 0 | + |
| trnS (gcu) | 14061 | 14128 | 68 |  |  | 58.82 | 0 | + |
| ND2 | 14129 | 15181 | 1053 | ATT | TAA | 68.63 | 1 | + |

Table I. The organization of the mitochondrial genome of *O. h. r.* Yunnan strain in sampling sites of Chuxiong City

| Genes | Location | | Length (bp) | Start codon | Termination codon | AT content (%) | Interval (bp) | Coding strand |
| --- | --- | --- | --- | --- | --- | --- | --- | --- |
|  | Start | Stop |  |  |  |  |  |  |
| **CX2-19** | | | | | | | | |
| COX1 | 1 | 1536 | 1536 | ATG | TAA | 62.89 | 16 | + |
| COX2 | 1553 | 2239 | 687 | ATG | TAA | 65.36 | 2 | + |
| trnD (guc) | 2242 | 2308 | 67 |  |  | 79.1 | 0 | + |
| ATP8 | 2309 | 2467 | 159 | ATG | TAA | 76.1 | 7 | + |
| ATP6 | 2475 | 3170 | 696 | ATG | TAA | 68.53 | 30 | + |
| trnM (cau) | 3201 | 3265 | 65 |  |  | 67.69 | 1 | - |
| trnY (gua) | 3267 | 3331 | 65 |  |  | 60 | 9 | - |
| trnC (gca) | 3341 | 3404 | 64 |  |  | 76.56 | 1 | - |
| trnW (uca) | 3406 | 3471 | 66 |  |  | 71.21 | 1 | - |
| trnQ (uug) | 3473 | 3534 | 62 |  |  | 62.3 | 3 | - |
| trnG (ucc) | 3538 | 3603 | 66 |  |  | 66.67 | 5 | - |
| trnE (uuc) | 3609 | 3675 | 67 |  |  | 68.66 | 0 | - |
| s-rRNA | 3676 | 4620 | 945 |  |  | 69.6 | -2 | + |
| trnV (uac) | 4619 | 4685 | 67 |  |  | 70.15 | 0 | + |
| l-rRNA | 4686 | 6030 | 1345 |  |  | 73.14 | 0 | + |
| trnL (uaa) | 6031 | 6098 | 68 |  |  | 73.53 | 0 | + |
| trnL (uag) | 6099 | 6167 | 69 |  |  | 76.81 | 0 | + |
| ND1 | 6168 | 7118 | 951 | ATG | TAG | 67.47 | -11 | + |
| trnP (ugg) | 7108 | 7174 | 67 |  |  | 64.18 | 9 | + |
| ND6 | 7184 | 7675 | 492 | ATT | TAG | 70.33 | 4 | + |
| CYTB | 7677 | 8819 | 1143 | ATT | TAG | 64.65 | 0 | + |
| trnS (uga) | 8820 | 8884 | 65 |  |  | 52.31 | 0 | + |
| trnT (ugu) | 8885 | 8950 | 66 |  |  | 72.73 | 7 | - |
| ND4L | 8958 | 9254 | 297 | ATG | TAG | 69.36 | 38 | + |
| ND4 | 9293 | 10618 | 1326 | ATA | TAA | 68.75 | 2 | + |
| trnH (gug) | 10621 | 10685 | 65 |  |  | 67.69 | 0 | + |
| ND5 | 10686 | 12398 | 1713 | ATG | TAG | 66.73 | 1 | + |
| trnF (gaa) | 12400 | 12466 | 67 |  |  | 65.67 | 68 | + |
| COX3 | 12535 | 13314 | 780 | ATG | TAA | 61.41 | 11 | + |
| trnK (uuu) | 13326 | 13393 | 68 |  |  | 73.53 | 19 | + |
| trnA (ugc) | 13413 | 13479 | 67 |  |  | 71.64 | 0 | + |
| trnR (ucg) | 13480 | 13548 | 69 |  |  | 60.87 | 17 | + |
| trnN (guu) | 13566 | 13632 | 67 |  |  | 67.16 | 11 | + |
| trnI (gau) | 13644 | 13710 | 67 |  |  | 65.67 | 0 | + |
| ND3 | 13711 | 14064 | 354 | ATG | TAA | 64.97 | 0 | + |
| trnS (gcu) | 14065 | 14132 | 68 |  |  | 58.82 | 0 | + |
| ND2 | 14133 | 15185 | 1053 | ATT | TAA | 68.92 | 1 | + |
| Table I. The organization of the mitochondrial genome of *O. h. r.* Yunnan strain in sampling sites of Chuxiong City (continued) | | | | | | | | |
| **CX2-20** | | | | | | | | |
| COX1 | 1 | 1536 | 1536 | ATG | TAA | 62.83 | 16 | + |
| COX2 | 1553 | 2239 | 687 | ATG | TAA | 65.21 | 2 | + |
| trnD (guc) | 2242 | 2308 | 67 |  |  | 79.1 | 0 | + |
| ATP8 | 2309 | 2467 | 159 | ATG | TAA | 76.1 | 7 | + |
| ATP6 | 2475 | 3170 | 696 | ATG | TAA | 68.53 | 30 | + |
| trnM (cau) | 3201 | 3265 | 65 |  |  | 67.69 | 1 | - |
| trnY (gua) | 3267 | 3331 | 65 |  |  | 60 | 9 | - |
| trnC (gca) | 3341 | 3404 | 64 |  |  | 76.56 | 1 | - |
| trnW (uca) | 3406 | 3471 | 66 |  |  | 71.21 | 1 | - |
| trnQ (uug) | 3473 | 3534 | 62 |  |  | 62.3 | 3 | - |
| trnG (ucc) | 3538 | 3603 | 66 |  |  | 66.67 | 5 | - |
| trnE (uuc) | 3609 | 3675 | 67 |  |  | 68.66 | 0 | - |
| s-rRNA | 3676 | 4620 | 945 |  |  | 69.6 | -2 | + |
| trnV (uac) | 4619 | 4685 | 67 |  |  | 70.15 | 0 | + |
| l-rRNA | 4686 | 6030 | 1345 |  |  | 73.14 | 0 | + |
| trnL (uaa) | 6031 | 6098 | 68 |  |  | 73.53 | 0 | + |
| trnL (uag) | 6099 | 6167 | 69 |  |  | 76.81 | 0 | + |
| ND1 | 6168 | 7118 | 951 | ATG | TAG | 67.47 | -11 | + |
| trnP (ugg) | 7108 | 7174 | 67 |  |  | 64.18 | 9 | + |
| ND6 | 7184 | 7675 | 492 | ATT | TAG | 70.33 | 4 | + |
| CYTB | 7677 | 8819 | 1143 | ATT | TAG | 64.65 | 0 | + |
| trnS (uga) | 8820 | 8884 | 65 |  |  | 52.31 | 0 | + |
| trnT (ugu) | 8885 | 8950 | 66 |  |  | 72.73 | 7 | - |
| ND4L | 8958 | 9254 | 297 | ATG | TAG | 69.36 | 38 | + |
| ND4 | 9293 | 10618 | 1326 | ATA | TAA | 68.75 | 2 | + |
| trnH (gug) | 10621 | 10685 | 65 |  |  | 67.69 | 0 | + |
| ND5 | 10686 | 12398 | 1713 | ATG | TAG | 66.73 | 1 | + |
| trnF (gaa) | 12400 | 12466 | 67 |  |  | 65.67 | 68 | + |
| COX3 | 12535 | 13314 | 780 | ATG | TAA | 61.41 | 11 | + |
| trnK (uuu) | 13326 | 13393 | 68 |  |  | 73.53 | 19 | + |
| trnA (ugc) | 13413 | 13479 | 67 |  |  | 71.64 | 0 | + |
| trnR (ucg) | 13480 | 13548 | 69 |  |  | 60.87 | 17 | + |
| trnN (guu) | 13566 | 13632 | 67 |  |  | 67.16 | 11 | + |
| trnI (gau) | 13644 | 13710 | 67 |  |  | 65.67 | 0 | + |
| ND3 | 13711 | 14064 | 354 | ATG | TAA | 64.97 | 0 | + |
| trnS (gcu) | 14065 | 14132 | 68 |  |  | 58.82 | 0 | + |
| ND2 | 14133 | 15185 | 1053 | ATT | TAA | 68.92 | 1 | + |
| **CX2-23** | | | | | | | | |
| COX1 | 1 | 1536 | 1536 | ATG | TAA | 62.83 | 16 | + |
| COX2 | 1553 | 2239 | 687 | ATG | TAA | 65.21 | 2 | + |
| trnD (guc) | 2242 | 2308 | 67 |  |  | 79.1 | 0 | + |
| Table I. The organization of the mitochondrial genome of *O. h. r.* Yunnan strain in sampling sites of Chuxiong City (continued) | | | | | | | | |
| ATP8 | 2309 | 2467 | 159 | ATG | TAA | 76.1 | 7 | + |
| ATP6 | 2475 | 3170 | 696 | ATG | TAA | 68.53 | 30 | + |
| trnM (cau) | 3201 | 3265 | 65 |  |  | 67.69 | 1 | - |
| trnY (gua) | 3267 | 3331 | 65 |  |  | 60 | 9 | - |
| trnC (gca) | 3341 | 3404 | 64 |  |  | 76.56 | 1 | - |
| trnW (uca) | 3406 | 3471 | 66 |  |  | 71.21 | 1 | - |
| trnQ (uug) | 3473 | 3534 | 62 |  |  | 62.3 | 3 | - |
| trnG (ucc) | 3538 | 3603 | 66 |  |  | 66.67 | 5 | - |
| trnE (uuc) | 3609 | 3675 | 67 |  |  | 68.66 | 0 | - |
| s-rRNA | 3676 | 4620 | 945 |  |  | 69.6 | -2 | + |
| trnV (uac) | 4619 | 4685 | 67 |  |  | 70.15 | 0 | + |
| l-rRNA | 4686 | 6030 | 1345 |  |  | 73.14 | 0 | + |
| trnL (uaa) | 6031 | 6098 | 68 |  |  | 73.53 | 0 | + |
| trnL (uag) | 6099 | 6167 | 69 |  |  | 76.81 | 0 | + |
| ND1 | 6168 | 7118 | 951 | ATG | TAG | 67.47 | -11 | + |
| trnP (ugg) | 7108 | 7174 | 67 |  |  | 64.18 | 9 | + |
| ND6 | 7184 | 7675 | 492 | ATT | TAG | 70.33 | 4 | + |
| CYTB | 7677 | 8819 | 1143 | ATT | TAG | 64.65 | 0 | + |
| trnS (uga) | 8820 | 8884 | 65 |  |  | 52.31 | 0 | + |
| trnT (ugu) | 8885 | 8950 | 66 |  |  | 72.73 | 7 | - |
| ND4L | 8958 | 9254 | 297 | ATG | TAG | 69.36 | 38 | + |
| ND4 | 9293 | 10618 | 1326 | ATA | TAA | 68.75 | 2 | + |
| trnH (gug) | 10621 | 10685 | 65 |  |  | 67.69 | 0 | + |
| ND5 | 10686 | 12398 | 1713 | ATG | TAG | 66.73 | 1 | + |
| trnF (gaa) | 12400 | 12466 | 67 |  |  | 65.67 | 68 | + |
| COX3 | 12535 | 13314 | 780 | ATG | TAA | 61.41 | 11 | + |
| trnK (uuu) | 13326 | 13393 | 68 |  |  | 73.53 | 19 | + |
| trnA (ugc) | 13413 | 13479 | 67 |  |  | 71.64 | 0 | + |
| trnR (ucg) | 13480 | 13548 | 69 |  |  | 60.87 | 17 | + |
| trnN (guu) | 13566 | 13632 | 67 |  |  | 67.16 | 11 | + |
| trnI (gau) | 13644 | 13710 | 67 |  |  | 65.67 | 0 | + |
| ND3 | 13711 | 14064 | 354 | ATG | TAA | 64.97 | 0 | + |
| trnS (gcu) | 14065 | 14132 | 68 |  |  | 58.82 | 0 | + |
| ND2 | 14133 | 15185 | 1053 | ATT | TAA | 68.92 | 1 | + |
| **CX2-24** | | | | | | | | |
| COX1 | 1 | 1536 | 1536 | ATG | TAA | 62.83 | 16 | + |
| COX2 | 1553 | 2239 | 687 | ATG | TAA | 65.21 | 2 | + |
| trnD (guc) | 2242 | 2308 | 67 |  |  | 79.1 | 0 | + |
| ATP8 | 2309 | 2467 | 159 | ATG | TAA | 76.1 | 7 | + |
| ATP6 | 2475 | 3170 | 696 | ATG | TAA | 68.53 | 30 | + |
| trnM (cau) | 3201 | 3265 | 65 |  |  | 67.69 | 1 | - |
| trnY (gua) | 3267 | 3331 | 65 |  |  | 60 | 9 | - |
| Table I. The organization of the mitochondrial genome of *O. h. r.* Yunnan strain in sampling sites of Chuxiong City (continued) | | | | | | | | |
| trnC (gca) | 3341 | 3404 | 64 |  |  | 76.56 | 1 | - |
| trnW (uca) | 3406 | 3471 | 66 |  |  | 71.21 | 1 | - |
| trnQ (uug) | 3473 | 3534 | 62 |  |  | 62.3 | 3 | - |
| trnG (ucc) | 3538 | 3603 | 66 |  |  | 66.67 | 5 | - |
| trnE (uuc) | 3609 | 3675 | 67 |  |  | 68.66 | 0 | - |
| s-rRNA | 3676 | 4620 | 945 |  |  | 69.6 | -2 | + |
| trnV (uac) | 4619 | 4685 | 67 |  |  | 70.15 | 0 | + |
| l-rRNA | 4686 | 6030 | 1345 |  |  | 73.14 | 0 | + |
| trnL (uaa) | 6031 | 6098 | 68 |  |  | 73.53 | 0 | + |
| trnL (uag) | 6099 | 6167 | 69 |  |  | 76.81 | 0 | + |
| ND1 | 6168 | 7118 | 951 | ATG | TAG | 67.47 | -11 | + |
| trnP (ugg) | 7108 | 7174 | 67 |  |  | 64.18 | 9 | + |
| ND6 | 7184 | 7675 | 492 | ATT | TAG | 70.33 | 4 | + |
| CYTB | 7677 | 8819 | 1143 | ATT | TAG | 64.65 | 0 | + |
| trnS (uga) | 8820 | 8884 | 65 |  |  | 52.31 | 0 | + |
| trnT (ugu) | 8885 | 8950 | 66 |  |  | 72.73 | 7 | - |
| ND4L | 8958 | 9254 | 297 | ATG | TAG | 69.36 | 38 | + |
| ND4 | 9293 | 10618 | 1326 | ATA | TAA | 68.75 | 2 | + |
| trnH (gug) | 10621 | 10685 | 65 |  |  | 67.69 | 0 | + |
| ND5 | 10686 | 12398 | 1713 | ATG | TAG | 66.73 | 1 | + |
| trnF (gaa) | 12400 | 12466 | 67 |  |  | 65.67 | 68 | + |
| COX3 | 12535 | 13314 | 780 | ATG | TAA | 61.41 | 11 | + |
| trnK (uuu) | 13326 | 13393 | 68 |  |  | 73.53 | 19 | + |
| trnA (ugc) | 13413 | 13479 | 67 |  |  | 71.64 | 0 | + |
| trnR (ucg) | 13480 | 13548 | 69 |  |  | 60.87 | 17 | + |
| trnN (guu) | 13566 | 13632 | 67 |  |  | 67.16 | 11 | + |
| trnI (gau) | 13644 | 13710 | 67 |  |  | 65.67 | 0 | + |
| ND3 | 13711 | 14064 | 354 | ATG | TAA | 64.97 | 0 | + |
| trnS (gcu) | 14065 | 14132 | 68 |  |  | 58.82 | 0 | + |
| ND2 | 14133 | 15185 | 1053 | ATT | TAA | 68.92 | 1 | + |
