# Supplementary material for: Genetic differentiation of Oncomelania hupensis robertsoni in hilly regions of China: Using the complete mitochondrial genome
Source: PLoS Negl Trop Dis. 2024 Nov 26;18(11):e0012094. doi: 10.1371/journal.pntd.0012094 (PMC11630586; doi:10.1371/journal.pntd.0012094)
Supplement: Attachment — Submitted filename: response.pdf [file pntd.0012094.s003.pdf]

Ref: Submission ID (PNTD-D-24-00426) - [EMID: a6df68801cc9ac4a]

Genetic differentiation of *Oncomelania hupensis robertsoni* in hilly regions of China: using the complete mitochondrial genome

PLOS Neglected Tropical Diseases

Dear Prof. Luc E. Coffeng,

Thank you for giving us the opportunity to revise our manuscript. We would also like to thank the editors for their careful review and thoughtful comments. We have revised the manuscript would like to re-submit it. The comments proposed by the reviewers were addressed and all the amendments are highlighted in red in the revised manuscript.

Based on the comments provided by editor and reviewers, we have modified our manuscript to meet with requirements of your journal. We are highly appreciative of the careful critique of the reviewer and hope that the improvements in this manuscript have sufficiently addressed all of your concerns. Our point-by-point responses are listed below.

We look forward to hearing from you soon.

With kindest regards,

Yours sincerely,

Yi Dong   Email: dydali@sina.com

Methods

Reviewer #1: The objective of the study, which is to sequence the complete mitochondrial genome of *Oncomelania hupensis robertsoni* and analyze the genetic differentiation of *Oncomelania hupensis robertsoni*, is clearly stated. The study design is appropriate in addressing the objective. The sample size and source population are likewise adequate. I have also listed below specific comments regarding the Methods section:

Comment 1: 144 This statement needs further clarity. Did the authors mean the live snails were distinguished and collected from the dead ones by the crawling method?

Response 1: We truly appreciate your time in reviewing our article, and thanks for your useful suggestion. Crawling method is one of the most commonly used methods to identify the survival status of the snail, first of all, the snail will be placed in the center of the petri dish with a diameter of 15cm, and dripping water on the snail, with a mesh cover to cover the petri dish to prevent the snail from crawling out. Place it at room temperature (25℃), and observe it after 24 hours, the one that can crawl out of the way is the live snail. As suggested, we have further clarified the statement about crawling method to make it clearer. (please see page 10, lines 163-168 in Manuscript with track change).

Comment 2: 146 Please clarify regarding the pleopod. I don’t think snails have pleopods, which are forked appendages typically found in crustaceans and are used for swimming.

Response 2: Thank you for your kind suggestion. We apologize for this confusion. As suggested, we have changed “pleopod” to “head-foot muscle”. (please see page 10, lines 169-170 in Manuscript with track change).

Comment 3: 151-152 Please clarify what you mean by ‘except for the reverse primer of primer pair 10 and the primer pair 14, which were not consistent with the literature.”

So did you mean you did not design them, or you did not include them in amplifying the specific fragments?

Response 3: Thank you for your kind suggestion. We apologize for this confusion. In our study, since the sequence could not be amplified using the reverse primer of primer pair 10 and the primer pair 14 from the literature[1], these three primers were redesigned. As suggested, we have revised the Primer design, PCR amplification and sequencing Section to make it more reliable. (please see page 10, lines 174-176 in Manuscript with track change).

Comment 4: 199-208 (1) Except for MP, how did you identify the optimal model for DNA substation for each tree construction method? Did you test for the optimal model, or did you just select a default model? If you use a selected model, did you do a partition homology test for uniform phylogenetic signal? (2) Did you use a single model for the entire genome, or did you partition the genome into segments, with each segment having its own model of DNA substitution?

Response 4: We thank the reviewer for these comments.

In our study, we used MEGA 11.0.9 software for phylogenetic tree construction. Since this software only provides the selection of the optimal model for the ML method, we selected the optimal model “General Time Reversible model” for the ML method using MODELS/Find Best DNA/Protein (ML); the other three methods (ME/NJ/MP) were not selected for the optimal model and used the default model. Moreover, we used a single model for the entire genome.

However, we are not sure if there are any misunderstanding about the partition homology test for uniform phylogenetic signal. Perhaps, the protein-coding genes and RNA genes in the mitochondrial genome were selected to construct separate phylogenetic trees, which were then compared with those constructed using the whole genome?

We still would like to take this opportunity to thank you again for all your time involved and this great opportunity for us to improve the manuscript.

Reviewer #2: consider current insights for annotation of molluscan mitogenomes as indicated in comments to authors. Specifically, employ visual check after automated mitos annotation, especially considering recommendations as provided by Fourdrilis et al., 2018; doi: 10.1038/s41598-018-36428-7 and Ghiselli et al 2020; DOI: 10.1098/rstb.2020.0159. Inspection of Genbank entries indicates potential errors in annotation. (That does not impact the phylogenetic analyses). See comment to authors.

Results

Reviewer #1: The analysis used was adequate. I have a few comments about the way some of the results are presented.

Comment 1: For Figure 3: (1) A more effective way to present the phylogenetic trees is to select one tree (e.g. ML tree) and incorporate the bootstrap values of all the tree construction methods for all the clades that appear in all. (2) Please remove '.1' in the accession numbers. (3) Please make sure that the figure can stand on its own. For instance, mention in the caption the outgroup taxon, what the values on node signify, and what the scale bar at the base of the tree mean.

Response 1: Thank you for your kind suggestion, these comments are very important. As suggested, we have revised the Figure 3 and Results Section to make it clearer.

In Figure 3, we have selected ML tree and incorporated the bootstrap values of all the tree (i.e., MP tree, ME tree, and NJ tree) for all clades that appear in each. Additionally, we have added the text in legends of Figure 3 to describe the outgroup taxon, clarified the significance of node values and the scale bar at the base of the tree mean. In our study, we selected the genotype from Philippines (*O. h. quadrasi*) as the outgroup taxon. The values on node signify refer to the bootstrap supports, and the scale bar at the base of the tree refer to the unit length of the sequence difference value in phylogenetic tree. (please see Figure 3)

Furthermore, in the Results Section, we have removed '.1' in the accession numbers. (please see page 13, lines 219-227 in Manuscript with track change)

Comment 2: For Table 2: You need to add a column to indicate what region/gene of the mitochondrial genome each primer pair amplifies and their expected PCR product range in bp.

Response 2: Thanks for pointing this out, this comment is very important. As suggested, we have added two columns to indicate the region of the mitochondrial genome each primer pair amplifies and their expected PCR product range in bp. (please see Table 2)

Reviewer #2: no concerns, see comments to authors

Conclusions

Reviewer #1: Though the results provide the first full mitochondrial genomic sequence for the Yunnan strain of *O. hupensis robersoni*, the heterogeneity they present for the snails based on the molecular data does not explain how the snail can be controlled. I think the discussion needs to incorporate how the genetic data can actually be used for developing strategies for schistosomiasis monitoring and control.

Response: Thank you for providing this comment which enlarge the value of this manuscript. As suggested, we have further discussed how the genetic data of *Oncomelania hupensis* (*O. hupensis*) can be used for developing strategies for schistosomiasis control. (please see page 20, lines 359-363, and page 18, lines 344-346 in Manuscript with track change)

O. hupensis snail is the sole intermediate host of *Schistosome japonicum*, and the current measures of schistosomiasis control primarily focused on the monitoring and control of *O. hupensis*. Our work reported the first mitochondrial genome of *Oncomelania hupensis robertsoni* Yunnan strain, which could be used as an important reference genome for *O. hupensis*, and also provide the important information for control of *Schistosoma japonicum*.

First of all, the genetic data of *O. hupensis* can be used for monitoring of schistosomiasis. Low host genetic differentiation can be associated with increased susceptibility to parasite infection[2, 3]. For example, in the Senegal River Basin,

perturbations (i.e., construction of the Diama dam) in population genetics that lead to decreased intrapopulation diversity could contribute to the major *Schistosome mansoni* outbreak[4].

Moreover, genetic differences between *O. hupensis* populations are associated with different environmental adaptations for survival[5]. The fact that snail populations in some regions are genetically more different from other regions implies that snails in that region have unique adaptations for survival or risk of schistosomiasis transmission.

Furthermore, genetic data of snails can also be used for targeted development of snail control drugs. Different populations of snails may differ in their susceptibility to certain drugs at the genetic level[6], and genetic data from snails can be used to optimize drug formulations and methods of use.

Findings of our study may provide important information for targeted formulation of schistosomiasis prevention and control measures.

Reviewer #2

Are the conclusions supported by the data presented?

Data presented do not support statement of noncoding AT region, clarification is needed whether authors refer to collection of small intergenic regions. Otherwise no concerns.

Are the limitations of analysis clearly described?

no concern although the paper can more clearly contrast the benefits, drawbacks of phylogenetics employing mitogenome versus nuclear genome data.

Do the authors discuss how these data can be helpful to advance our understanding of the topic under study?

Modestly.

Is public health relevance addressed?

Role of *O hupensis* as vector for schistosomiasis is considered, discussion could include consideration of how genetic diversity relates to disease transmission.

Editorial and Data Presentation Modifications?

Reviewer #1: The results and discussion sections can be merged to minimize repetitiveness of the data/results mentioned. Other minor editorial modifications are listed below.

Response: Thank you for your kind suggestion. As suggested, we have trimmed the repetitiveness of results/data that appeared in the Discussion Section to make it more concise. (please see Manuscript with track change)

Line no. Comment: 53 Change to 'Philippine'; 57 Change to 'sub-branches'; 75-77 All subsequent Schistosoma after Schistosoma japonicum can be abbreviated to S.; 83 Change to 'inhabit'; 89 Add 'and' after '(O. h. tangi),'; 97 Change to 'Province'; 98 Remove ',' after 'China'; 101 Shouldn't 'province' be capitalized? 106 Change 'in areas of' to 'particularly in areas such as'; 124 Remove ',' after 'O. hupensis'; 141 Add 'in' after 'presented'; 142 Remove 'were'; 175 Did you mean 'mitochondrial genome' for the 'mitochondrial gene' mentioned here? 176 Please indicate here the subspecies of the reference *O. hupensis* mitochondrial genome.; 190-198 Please remove '.1' in all the accession numbers.; 230 Remove 'was'; 236 Add 'in' after 'presented'; 237 Change 'weas' to 'were'; 244 Change to 'is presented in Figure 3.'; 249 Change to 'sub-branches'; 255 Add 'regions' after 'southern'; 265 Change to 'Branch'; 275 Change to 'Discussion'; 277 Change 'is' to 'are'; 279 Add 'useful' after 'might be'; 283 Change to 'results'; 290 Add 'and' after 'maternal inheritance'; 361 Change to '*O. hupensis*. This…"; 434-601 All generic and scientific names in the list of references should be italicized.

Response: Thank you for your kind and valuable suggestion. As suggested, we have revised these sentences and checked the manuscript carefully. (please see Manuscript with track change)

Reviewer #2: Revisions invited are textual, (also ask for additional technical details) but also include some re-analysis of annotation. I consider this a major revision.

Summary and General Comments

Reviewer #1: The study provides the full mitochondrial genome of *O. hupensis robertsoni* from Yunnan Province, which allows its placement among the different strains of *O. hupensis robertsoni*. Though the writers hinted at the possibility of using the genetic information to control the snail, which is an important vector of Schistosoma japonicum, this was not clearly expounded in the paper. I would have wanted that this be further discussed in order to justify obtaining the full mitochondrial genome from 26 individuals. I have also listed below some minor comments to help improve the paper.

Comment 1: 110-112 It is not stated why heterogeneity of the snail can pose 'challenges to monitoring and controlling the snails'. Perhaps the authors need to explain this briefly in order to justify the need to explore genetic variation among the different populations of *O. h. robertsoni*.

Response 1: Thanks for pointing this out, this comment is very important. Previous studies have shown that genetic variation among the different populations of *O. hupensis* snail is associated with different environmental adaptations for survival and susceptibility to parasite infection[2, 3], which poses a challenge for the development of targeted snail monitoring and controlling. As suggested, we have further explained the need to explore genetic variation among the different populations of *O. h. robertsoni*. (please see page 7, line 126 in Manuscript with track change)

Comment 2: 114-118 The authors should mention that the 16S and 12S markers were also used to differentiate the subspecies of *O. hupensis*. These include the following:

Chua JC, Fontanilla IKC, Tabios IKB, Tamayo PG, De Chavez ERC, Agatsuma T, Kikuchi M, Kato-Hayashi N, Chigusa Y, Fornillos RJC, Leonardo LR. 2017. Genetic comparison of *Oncomelania hupensis quadrasi* (Mollendorf, 1895)

(Gastropoda: Pomatiopsidae), the intermediate host of Schistosoma japonicum in the Philippines based on 16S ribosomal RNA sequence. Science Diliman 29(2): 32-50.

Okamoto M, Lo CT, Tiu WU, Qui D, Hadidjaja P, Upatham S, Sugiyama H, Taguchi T, Hirai H, Saitoh Y, Habe S, Kawanaka M, Hirata M, Agatsuma T. 2003. Phylogenetic relationships of snails of the genera *Oncomelania* and *Tricula* inferred from the mitochondrial 12S RNA gene. Japanese Journal of Tropical Medicine and Hygiene. 31(1):5-10.

Wilke T, Davis GM, Dongchuan Q, Spear RC. 2006. Extreme mitochondrial sequence diversity in the intermediate schistosomiasis host *Oncomelania hupensis robertsoni*: Another case of ancestral polymorphism? Malacologia. 48:143-157.

Response 2: Thank you for your kind and valuable suggestion. We apologize for the omission of the literature review. As suggested, we have revised the relevant text and mentioned the 12S and 16S ribosomal RNA sequence when describing the genetic markers used to differentiate the subspecies of *O. hupensis*. (please see page 8, lines 131-132 in Manuscript with track change)

Comment 3: 256-258 Please describe in the text the barriers you mention that separate the two sub-branches. Perhaps you are referring to what you described in your discussion section regarding the Jonsha River Watershed?

Response 3: Thank you for your kind suggestion. These barriers that separate the Yunnan North and South sub-branches include the high mountains and canyons formed by the Hengduan and Yunling Mountain ranges. As suggested, we have revised the relevant text. (please see page 16, lines 289-290 in Manuscript with track change)

Comment 4: 318-325 Maybe you can also refer to other previous studies that used other markers such as 16S and 12S genes, notably Chua et al. 2017. They also analyzed several sequences of *O. hupensis robertsoni* using 16S.

Response 4: Thank you for your kind suggestion. As suggested, we have referred previous studies that used 16S ribosomal RNA gene in the Discussion Section. (please see page 18, line 323 in Manuscript with track change)

However, the taxonomic results of *O. h. robertsoni* in the study that used 12S ribosomal RNA gene was inconsistent with the currently recognized taxonomic results[3, 7], and the reliability of the results was limited by the small bootstrap support value of the phylogenetic tree, so we didn't cite the study that used 12S ribosomal RNA gene (Okamoto M, Lo CT, Tiu WU, Qui D, Hadidjaja P, Upatham S, Sugiyama H, Taguchi T, Hirai H, Saitoh Y, Habe S, Kawanaka M, Hirata M, Agatsuma T. 2003. Phylogenetic relationships of snails of the genera Oncomelania and Tricula inferred from the mitochondrial 12S RNA gene. Japanese Journal of Tropical Medicine and Hygiene. 31(1):5-10).

Comment 5: 350-351 Please clarify what you mean by 'unique natural factor in the Jinsha River Watershed or geographic isolation'. Please provide details regarding these and why they could contribute to the genetic differentiation of the two subgroups. You can hypothesize, which can be tested in subsequent research.

Response 5: Thank you for your kind suggestion. As suggested, we have further clarified the unique natural factor that may contribute to the north-south differentiation of *Oncomelania hupensis robertsoni* Yunnan strain, including soil salinity level, PH, and wetness, etc[5]. (please see page 21, lines 394-395 in Manuscript with track change)

Comment 6: 359-360 Isn't the genetic difference big enough to warrant separating them into two subspecies rather than subgroups with *O. hupensis robersoni*? Do you have threshold values for delineating subspecies based on the whole mitochondrial genome? For the COI gene, some authors suggest using the 3% threshold, though this may vary depending on the taxonomic group.

Response 6: Thank you for your kind suggestion. To the best of our knowledge, there is no threshold value for delineating subspecies based on the whole mitochondrial

genome. Previous taxonomy studies of *Oncomelania hupensis* snails based on shell morphology and techniques of isolation and identification at the cellular, protein, and molecular levels have divided the *Oncomelania hupensis robertsoni* into two subspecies[3, 7, 8], *Oncomelania hupensis robertsoni* Yunnan strain and Sichuan strain. Hence, we believe that *Oncomelania hupensis robertsoni* Yunnan strain should be categorized as a subspecies, with two subgroups that have evolved internally.

Reviewer #2: This report describes characterization of full mitogenomes from a number of *Oncomelania hupensis robertsoni* (*Ohr*), vector snails for the parasite Schistosoma japonicum, field collected from an endemic area in China. Sequence data were used in phylogenetic analyses relative to previous GenBank entries for *Oncomelania hupensis* mitogenomes to investigate population structure in the field. Results may inform transmission dynamics, tracking of populations and future control efforts.

Notably, the mitogenome sequencing did not rely on computational assembly of short reads but employed a robust approach involving PCR amplification and Sanger sequencing of overlapping amplicons to generate genome assemblies with high confidence. This helps avoid computational bioinformatics errors that may result with automated assembly of short reads, such as “stacking up” of repetitive regions (e.g. Sharbrough et al., 2023; https://doi.org/10.1093/molbev/msad007) or incorporation of NUMTs (e.g. Wei et al, 2022; https://doi.org/10.1038/s41586-022-05288-7)

The authors are encouraged to include a consideration of how the diversity of these snails and other previously characterized snails reflect the transmission dynamics (efficiency, intensity) of schistosomiasis.

Some wording and aspects of experiments can be improved for clarity of the paper. Authors are asked to address the following comments:

Abstract,

Comment 1: Delete “hilly regions” or use alternative to indicate geographical barriers?

Response 1: We truly appreciate your time in reviewing our article, and thanks for your kind suggestion. In China, based on geomorphological features, *Oncomelania hupensis* (*O. hupensis*) habitats are classified into three types, namely, lake, hill, plain water network region[9]. Different types of *O. hupensis* have different shell morphology, genetic diversity, ecological habits, etc[10]. In our manuscript, "hilly regions" is a geographic description of *O. hupensis*, not the geographical barriers, so no modifications were made.

Comment 2: Delete "the" from the genetic differentiation; the mitogenome does not inform on nuclear genome differentiation

Response 2: Thank you for your kind suggestion. As suggested, we have revised this sentence (please see page 3, line 42 in Manuscript with track change).

Comment 3: Considering that no data resulted from one location, suggest to edit from 14 to "13 villages in Yunnan",

Response 3: Thank you for your kind suggestion. As suggested, we have revised this sentence (please see page 3, line 44 in Manuscript with track change).

Comment 4: Similarly of 30 snails collected, only 2 individuals were used for mitogenome sequencing, adjust numbers accordingly

Response 4: We apologize for the confusion caused by our unclear text. In our study, instead of only 2 snails were used for mitogenome sequencing, all 30 snails collected were used for mitogenome sequencing.

Firstly, we selected 13 sampling sites and collected about 100 O. hupensis snails per sampling site, randomly selected 30 live snails after removing the dead snails by crawling method for DNA extraction.

Next, the sequence of the extracted DNA was obtained after PCR amplification, and the completed mitochondrial whole genome sequence was obtained after splicing and assembling the sequenced 16 gene sequences. In foregoing process, sequence amplification failure may occur when the PCR amplification, "bimodal mutation"

may occur when the sequencing, and the sequence at both ends of the splicing may fail to connect when the Sequence assembly, which lead to failure to obtain the mitochondrial whole genome sequence of the conch samples. Therefore, for the 30 snails per sampling site initially used for DNA extraction, the complete mitochondrial genome sequences of only 1-4 snail samples could be obtained in the end. Finally, we obtained 26 complete mitochondrial genome sequences from 13 sampling sites, 26 ≠ 2 for each collection site.

As suggested, we have added the detailed information in Additional file to make it clearer. (please see Additional file 2: Detailed information about the organization of 26 mitochondrial genome of *O. h. robertsoni*)

Comment 5: The non-coding sequence rich in AT is not evident in description, figures or GenBank entries. Provide relevant information.

Response 5: We apologize for the confusion caused by the inaccurate text. This non-coding sequence rich in A+T refers to the non-coding region between trnF(gaa) and COX3, which we named the non-coding region rich in A+T by reference to previous study[1] when we finished the manuscript.

As suggested, we first counted the A+T content of this region and found that the A+T content was about 72.06%, similar to the 70.83% in the previous study[1]. However, the A+T content of this region is less than that of most of the 37 genes in the mitochondrial genome, and we believe that it is inaccurate to refer to this region as the “region rich in A+T”. Considering that this region is a non-coding region, we have deleted the relevant text to make our manuscript more clear and reliable. (please see page 3, lines 52-53 in Manuscript with track change).

Comment 6: Mentioning of the outgroup: as “the Phillipines genotypes” is a confusing designation; and seems an unnecessary detail for the abstract.

Response 6: Thank you for your kind suggestion. As suggested, we have deleted the “the Phillipines genotypes”. (Please see page 3, line 53 in Manuscript with track change)

Comment 7: Was the comparison/phylogenetic analysis based on full-length genomes?

Response 7: We apologize for the confusion caused by our unclear text. In our study, the comparison/phylogenetic analysis was based on full-length genomes.

Comment 8: Instead of homology, a better term mat be identity when comparing DNA sequences.

Response 8: Thank you for your kind suggestion. As suggested, we have changed “homology” to “identity” in our manuscript. (please see Manuscript with track change)

Introduction

Comment 9: 93 delete “fully”. The scope of this paper cannot provide that.

Response 9: Thank you for your kind suggestion. As suggested, we have revised this sentence (please see page 7, line 107 in Manuscript with track change).

Comment 10: 97,99 Yunnan Provinces or Province, two or one entities? Also considering the map, is this a West-region or South-West region of China.

Response 10: Thank you for your kind comment. We apologize for this confusion caused by our unclear text. As suggested, we have changed “Yunnan Provinces” to “Yunnan Province”, and “western” to “south-west”. (please see page 7, lines 111-115 in Manuscript with track change).

Comment 11: 118 These references deal with blackflies, as possible, cite relevant papers from molluscan work.

Response 11: Thank you for your kind suggestion. As suggested, we have revised the reference and sited the relevant paper about *Oncomelania hupensis* work[1]. (please see page 8, line 133 in Manuscript with track change).

Comment 12: 119 Include mention of recent genome report (Liu et al 2021; 10.1186/s40249-024-01187-3)

Response 12: Thank you for your kind suggestion. As suggested, we have cited this article. (please see page 8, line 134 in Manuscript with track change).

Material and Methods.

Comment 13: Again, consider making numbers consistent for number of sites, snails collected per site, live snail selected by crawling methods,? number of snail extracted, the 2(?) snails sequenced per site.

Response 13: We apologize for the confusion caused by our unclear text.

In our study, we selected 13 sampling sites and collected about 100 *O. hupensis* snails per sampling site, randomly selected 30 live snails after removing the dead snails by crawling method for DNA extraction.

Next, the sequence of the extracted DNA was obtained after PCR amplification, and the completed mitochondrial whole genome sequence was obtained after splicing and assembling the sequenced 16 gene sequences. In this process, sequence amplification failure may occur when the PCR amplification process, "bimodal mutation" may occur when the sequencing process, and the sequence at both ends of the splicing may fail to connect when the Sequence assembly process, which lead to failure to obtain the mitochondrial whole genome sequence of the conch samples. Therefore, for the 30 snails per sampling site initially used for DNA extraction, the complete mitochondrial genome sequences of only 1-4 snail samples could be obtained in the end. Finally, we obtained 26 complete mitochondrial genome sequences from 13 sampling sites.

As suggested, we have added the detailed information in the Material and methods Section. (please see the Material and methods Section in Manuscript with track change)

Comment 14: 140 habit = habitat?

Response 14: Thank you for your kind comment. We apologize for this confusion caused by our unclear text. As suggested, we have changed "habit" to "habitat". (please see page 9, line 157 in Manuscript with track change).

Comment 15: 144-5 Genus name in full at start of sentence. Also edit sentence for meaning. Provide brief explanation of "crawling method" additional to citations.

Response 15: Thank you for your kind comment. As suggested, we have revised these sentences and added the detailed information about crawling method. (please see page 9, line 161, and page 10, lines 162-168 in Manuscript with track change).

Crawling method[11, 12] is one of the most commonly used methods to identify the survival status of the snail, first of all, the snail will be placed in the center of the petri dish with a diameter of 15cm, and dripping water on the snail, with a mesh cover to cover the petri dish to prevent the snail from crawling out. Place it at room temperature (25℃), and observe it after 24 hours, the one that can crawl out of the way is the live snail.

Comment 16: 146-7 suggest edit "of a single snail" of individual snails". How were 2(?) snails selected from the 30 snails/site for mitogenome sequencing? Indicate the specific "Qiagen extraction kit" (the website lists over 30 different kits)

Response 16: Thank you for your kind comment. As suggested, we have revised "of a single snail" of individual snails" and "Qiagen extraction kit" of "Qiagen DNeasy Blood & Tissue Kit". (please see page 10, lines 169-170 in Manuscript with track change). Additionally, the detailed information about the sample snails for mitogenome sequencing has been responded to in **Comment 13**. (please see **Response 13**)

Comment 17: 149 edit to "The entire mitogenome was…"

Response 17: Thank you for your kind comment. As suggested, we have revised this word. (please see page 10, line 173 in Manuscript with track change).

Comment 18: 151-2 explain this cryptic statement. Indicate what gene or region was different sequence-wise from "the literature [31]. How was it different and what was the origin of the snail used in that study?

Response 18: Thank you for your kind suggestion. We apologize for this confusion. In our study, since the sequence could not be amplified using the reverse primer of primer pair 10 and the primer pair 14 from the literature, these three primers were redesigned. As suggested, we have revised the Primer design, PCR amplification and sequencing Section to make it more reliable. (please see page 10, lines 174-176 in Manuscript with track change). The snails are *O. h. robertsoni* from Yunnan Province of China in our study, the snails are *O. h. hupensis*, *O. h. tangi*, and *O. h. robertsoni* form the other 9 Provinces of China, these snails is origin from *Oncomelania hupensis*.

Comment 19: 155 identify the type/kit of polymerase used. Importantly, instead of volumes indicate final concentrations of the reagents.

Response 19: Thank you for your kind suggestion. As suggested, we have identified the type/kit of polymerase used as DreamTaq Green DNA polymerase, and used final concentrations of the reagents to describe the PCR reaction system. (please see page 10, line 170 in Manuscript with track change)

Comment 20: 162 explian bimodal mutation as sequencing problem.

Response 20: Thanks for pointing this out, this comment is very important. "Bimodal mutation" in the sequencing result refers to the presence of two major peaks, making it impossible to accurately determine the sequencing result. As suggested, we have added the explain bimodal mutation to make it more reliable. (please see page 12, lines 190-191 in Manuscript with track change)

Comment 21: 164 “to splice” = “to contig assemble”?

Response 21: Thank you for your kind suggestion. As suggested, we have revised this sentence. (please see page 12, line 192 in Manuscript with track change)

Comment 22: 170 submission is not for alignment and direction confirmation? Does this refer to identification of open reading frames?

Response 22: Thanks for pointing this out, this comment is very important. The submission is for alignment and direction confirmation of the sequences. In our study, after contig assemble the 16 mitochondrial gene sequences using LaserGene 7.1.0 (DNAStar, Madison, Wisconsin, USA) software, the start genes of the whole sequence were random. Therefore, it was necessary to submit the sequences to NCBI for alignment, and the gene order of the sequences was re-adjusted, all the sequences were adjusted to have a start gene of COX1, which facilitated the subsequent analysis of all the sequences.

Comment 23: 171-174 sentence not informative, delete?

Response 23: Thank you for your kind suggestion. As suggested, we have deleted this sentence. (please see page 12, lines 199-202 in Manuscript with track change)

Comment 24: 174 “nearest” = “most similar”?

Response 24: Thank you for your kind suggestion. As suggested, we have deleted this sentence. (please see page 12, line 202 in Manuscript with track change)

Comment 25: 177 if sequences were trimmed, what information of the circular mitogenome was deleted? If no removal, suggest delete the term “trimmed”. Perhaps state, the mitogenomes sequences were oriented with the start of cox1 in the first position.

Response 25: Thanks for pointing this out, this comment is very important. Information of the circular mitogenome was not been deleted, we used COXI gene as the starting point of the mitogenomes sequences. As suggested, we have changed “trimmed” to “adjusted”. (please see page 12, line 206 in Manuscript with track change)

Comment 26: 178 define self-test.

Response 26: We apologize for the confusion caused by the unclear text. We defined sequences that we assembled and adjusted the COXI gene as a starting point as self-

test sequence. As suggested, we removed this word to avoid confusion. (please see page 12, line 207 in Manuscript with track change)

Comment 27: 182 Mitos automated sequencing is a good start but annotation requires visual checking: for recommendations see: Fourdrilis et al., 2018; doi: 10.1038/s41598-018-36428-7 and Ghiselli et al 2020; DOI: 10.1098/rstb.2020.0159. For instance, your Genbank entry OR661787: the region between ND6 and CYTB includes one more potential start codon that would extend the CYTB ORF by three nucleotides and one AA,. Ignoring this start codon goes against the genetic code, and reports a trunctated CYTB 5' sequence, and provides an incorrect start codon (ATG versus ATT). This also impacts the discussion of the number of nucleotides in intergenic, non-coding regions. Please check ALL gene ORFs and revise accordingly.

ND6 CYTB

I * (M) M R

ATT TAG C ATT ATG CGC

Response 27: Thank you for your kind suggestion. As suggested, we have visual checked all mitochondrial genome sequences. The base of OR661787 sequence is GCATT between gene ND6 and CYTB gene. According to the literature provided by the reviewer, the literature does mention "ATY" as a possible codon. However, when we aligned the mitochondrial sequences in NCBI, we found that the start codon of the mitochondrial CYTB gene was ATG, so we used ATG as the start codon as well.

Comment 28: 2.5 Construction of phylogenetic tree: what alignment program was used, were the mitogenomes aligned full length or was the analysis based on a partial sequences?

Response 28: Thanks for pointing this out, this comment is very important. In our study, we used ClustalW algorithm in MEGA11.0.9 software, and construction of phylogenetic tree was based on the full length of mitogenomes. As suggested, we have added the detailed information about construction of phylogenetic tree to make it clearer. (please see page 13, line 214 in Manuscript with track change)

Comment 29: NJ is not frequently used, what is the rational for including it here?

Response 29: We thank the reviewer for these comments. By reviewing the relevant literature, we found that the Neighbor-Joining (NJ) method is also a common finding for construction of phylogenetic tree[1, 13-15]. We would like to tentatively not remove the results of phylogenetic tree constructed by the NJ method.

Comment 30: 2.6 Sequence genetic distance and homology analysis. Again indicate if analyses used alignment of full length versus partial mitogenomes.

Response 30: Thank you for your kind suggestion. As suggested, we have revised the relevant text. (please see Manuscript with track change)

Results

Comment 31: 227 26 = 2 for each collection site? Explain the problem to obtain the/any sequences form Dianzhong.

Response 31: Thank you for your kind suggestion. 26 ≠ 2 for each collection site. In our study, 30 randomly selected *O. hupensis* snail samples from each sampling site were used for sequencing; however, due to sequence amplification failures, bimodal mutation, and failure of sequences to be spliced into loops, the number of mitochondrial genomes acquired varied from site to site, and not 2 snails from each site were selected for sequencing. Additionally, the detailed information about the sample snails for mitogenome sequencing has been responded to in Comment 13. (please see Response 13)

The snails from Dianzhong did not obtain any sequence also because of the above reasons. As suggested, we have deleted the Dianzhong sampling site in our study to avoid confusion.

Comment 32: 232 delete “significantly”. Not tested statistically and invertebrate mitogenomes typically have A+T >G+C.

Response 32: Thank you for your kind suggestion. We feel sorry for this mistake. As suggested, we have deleted "significantly". (please see page 15, line 261 in Manuscript with track change)

Comment 33: 234 "a non-coding region rich in A+T". It is unclear from the paper if this is a region independent from the intergenic regions described in 237-239.

If this a region such as described by Sharbough et al., 2023; https://doi.org/10.1093/molbev/msad007, provide details in the paper for location, length, possible repeat content. If this "region" refers to the intergenic regions described in 237-239, correct/revise the manuscript accordingly to clarify this unequivocally.

Include description: were the two mitogenomes from each location most similar to each other or do comparisons reveal another more complex pattern? Can this be indicated in the (legend of the) tree?

Response 33: We apologize for the confusion caused by the inaccurate text. This non-coding sequence rich in A+T refers to the non-coding region between trnF(gaa) and COX3, which we named the non-coding region rich in A+T by reference to previous study[1] when we finished the manuscript.

As suggested, we first counted the A+T content of this region and found that the A+T content was about 72.06%, similar to the 70.83% in the previous study. However, the A+T content of this region is less than that of most of the 37 genes in the mitochondrial genome, and we believe that it is inaccurate to refer to this region as the "region rich in A+T". Considering that this region is a non-coding region, we have deleted the relevant text to make our manuscript more clear and reliable. (please see Manuscript with track change)

Comment 34: 3.2 Phylogenetic tree construction: Provide the other 3 trees as supplementary results; Suggest to indicate these groupings on the map in figure 4.

Response 34: We truly appreciate your time in reviewing our article. According to your suggestions, we have revised the Figure section and Results section to make it clearer.

In our study, the topological structures of phylogenetic trees constructed by the four methods of Maximum Parsimony (MP), Maximum Likelihood (ML), Minimum Evolution (ME), and Neighbor-Joining (NJ) were consistent, and the bootstrap values for the same branch differ when constructed using different methods. Therefore, in Figure 3, we have selected ML tree and incorporated the bootstrap values of all the tree (i.e., MP tree, ME tree, and NJ tree) for all clades that appear in each to effectively present our result of phylogenetic trees, and we did not add the other 3 trees besides the ML method as supplementary results. (please see in Figure 3).

Additionally, we have added the phylogenetic tree topology of *Oncomelania hupensis robertsoni* Yunnan strain on the map in figure 4 according to your suggestion. (please see Figure 4).

Comment 35: 268: homology or identity?

Response 35: Thank you for your kind suggestion. As suggested, we have changed "homology" to "identity" in our manuscript. (please see Manuscript with track change)

Comment 36: 271-3 reword sentence to clarify "formed a rectangle in the lower right corner".

Response 36: Thank you for your kind suggestion. As suggested, we have revised this Section. (please see page 17, lines 299-305 in Manuscript with track change)

Discussion

Comment 37: 280 edit to "collected 14 representative, and recovered sequences from snails from 13 of these locations". Provide interpretation for why these sequences were unsuccessful?

Response 37: Thank you for your kind suggestion. The *O. hupensis* snails from Dianzhong did not obtain any sequence also because of sequence amplification

failures, bimodal mutation, and failure of sequences to be spliced into loops. As suggested, we have deleted the Dianzhong sampling site in our study to avoid confusion. (please see Manuscript with track change)

Comment 38: 286-291. Include the contrasting concept that mitogenomes also have drawbacks and may be used complementary with nuclear genes for optimal results.

Response 38: Thank you for your kind suggestion. As suggested, we have further described the mitogenomes. (please see page 20, lines 364-370 in Manuscript with track change)

Mitochondrial DNA is an organelle gene, and its DNA content is relatively small compared with that of nuclear genes[16, 17], which provides limited genetic information. However, our result of phylogenetic tree of *O. h. r.* Yunnan strain is consistent with the other study using microsatellite markers[8], suggesting that the mitochondrial genome and nuclear genes can be used reliably in the genetic evolutionary analysis of the *O. hupensis*.

Comment 39: 292-297 Information seems duplicated from introduction. Reduce in one of these sections.

Response 39: Thank you for your kind suggestion. As suggested, we have reduced this Section. (please see Discussion Section in Manuscript with track change)

Comment 40: 323 what is “foregoing”?

Response 40: We apologize for the confusion caused by the unclear text. As suggested, we have changed “foregoing results” to “our results”. (please see page 20, line 355 in Manuscript with track change)

Comment 41: 326 edit “could be subdivided” to “is subdivided”

Response 41: Thank you for your kind suggestion. As suggested, we have revised this Section. (please see page 20, line 366 in Manuscript with track change)

Comment 42: 341 avoid contractions

Response 42: Thank you for your kind suggestion. As suggested, we have revised this Section. (please see page 21, line 384 in Manuscript with track change)

Comment 43: 346-357, provide hypothesis/es for lack of diverhgence despite obvious geographical barriers; can there be transport of snails by wildlife or human activity? What is the age of the barriers…? Other?

Response 43: Thank you for your kind suggestion. As suggested, we have revised this Section and added the hypothesis. The geographic barrier between "North Yunnan Branch" and "South Yunnan Branch" of *O. h. r.* Yunnan strain is the high mountains and canyons formed by the Hengduan and Yunling Mountain ranges. The divergence between the northern and southern lineages of Yunnan snails may result from either unique natural factor in the Jinsha River Watershed such as different soil salinity level, PH, and wetness, or geographic isolation due to the significant distance separating the two lineages. (please see page 21, lines 394-395 in Manuscript with track change)

Comment 44: 366-369 OK to list the components of the effort but it seems unusual to state that these are major strengths of this study, Suggest rewording. Suggest mentioning that the methods (PCR, sequencing of overlapping sequence regions for assembly) avoid the computational challenges and possible errors of computational assembly of short reads, see Sharbrough 2023).

Response 44: Thanks for this valuable suggestion. Indeed, we trimmed the sequencing of overlapping sequence regions and manually checked the entire sequence to ensure a high level of accuracy in our results. As suggested, we have revised this Section. (please see page 22, lines 410-416 in Manuscript with track change)

Comment 45: 370- clarify how elimination of some snail is a limitation to this study. What hypothesis do you have of how that mitogenomic information would have shaped understanding of phylogeny?

Response 45: Thank you for your kind suggestion. The *O. hupensis* snails in Gejiu city, located in the southern part of Yunnan Province, have been eliminated; Gejiu city was relatively isolated within the previous endemic regions of schistosomiasis. We hypothesis that it may be a separate population for which clustering in the phylogenetic tree, genetic distances and homologies with other snail populations of the *Oncomelania hupensis robertsoni* were unavailable, which poses a limitation on the completeness of our study of the genetic differentiation of *Oncomelania hupensis robertsoni*. As suggested, we have revised this Section to make it clearer. (please see page 23, lines 421-423 in Manuscript with track change)

Comment 46: 383 edit “These finding may provide the..”: to “These findings provide ..”

Response 46: Thank you for your kind suggestion. As suggested, we have revised this Section. (please see page 23, line 431 in Manuscript with track change)

Comment 47: 388 edit “used to the important” to “as”

Response 47: Thank you for your kind suggestion. As suggested, we have revised this Section. (please see page 23, line 436 in Manuscript with track change)

References:

1. Shizhu L, Li Z, LiN M, Wei H, Shan L, Qin L, Yingjun Q, Qiang W, Xiaonong Z: **Phylogenetic performance of mitochondrial protein‑coding genes of *Oncomelania hupensis* in resolving relationships between landscape populations**. *J SYST EVOL* 2013, **3**(51):353-364.
2. Jarne P, Théron A: **Genetic structure in natural populations of flukes and snails: a practical approach and review**. *PARASITOLOGY* 2001, **123 Suppl**:S27-S40.
3. Li ZJ, Ge J, Dai JR, Wen LY, Lin DD, Madsen H, Zhou XN, Lv S: **Biology and Control of Snail Intermediate Host of *Schistosoma japonicum* in The People's Republic of China**. *Adv Parasitol* 2016, **92**:197-236.
4. Campbell G, Noble LR, Rollinson D, Southgate VR, Webster JP, Jones CS: **Low genetic diversity in a snail intermediate host (Biomphalaria pfeifferi Krass, 1848) and schistosomiasis transmission in the Senegal River Basin**. *MOL ECOL* 2010, **19**(2):241-256.
5. Vercruysse J, Southgate VR, Rollinson D, De Clercq D, Sacko M, De Bont J, Mungomba LM: **Studies on transmission and schistosome interactions in Senegal, Mali and Zambia**. *Trop Geogr Med* 1994, **46**(4 Spec No):220-226.
6. Colley DG, Jacobson JA, Binder S: **Schistosomiasis Consortium for Operational Research and Evaluation (SCORE): Its Foundations, Development, and Evolution**. *AM J TROP MED HYG* 2020, **103**(1_Suppl):5-13.
7. Xiaonong Z, Yi Z, Qingbiao H, Jide X, Tianping W: **Science on *Oncomelania* Snail**. Beijing: China Science Publishing and Media Ltd; 2005.
8. Song J, Wang H, Li S, Du C, Qian P, Wang W, Shen M, Zhang Z, Zhou J, Zhang Y *et al*: **The genetic diversity of *Oncomelania hupensis robertsoni*, intermediate hosts of *Schistosoma japonicum* in hilly regions of China, using microsatellite markers**. *Parasit Vectors* 2024, **17**(1):147.
9. Zhou Y, Chen Y, Jiang Q: **History of Human Schistosomiasis (bilharziasis) in China: From Discovery to Elimination**. *ACTA PARASITOL* 2021, **66**(3):760-769.

10. Wang CZ, Lu DB, Guo CX, Li Y, Gao YM, Bian CR, Su J: **Compatibility of *Schistosoma japonicum* from the hilly region and *Oncomelania hupensis hupensis* from the marshland region within Anhui, China**. *PARASITOL RES* 2014, **113**(12):4477-4484.

11. Zhao QP, Zhang SH, Deng ZR, Jiang MS, Nie P: **Conservation and variation in mitochondrial genomes of gastropods *Oncomelania hupensis* and *Tricula* hortensis, intermediate host snails of Schistosoma in China**. *MOL PHYLOGENET EVOL* 2010, **57**(1):215-226.

12. Dhara K, Saha S, Pal P, Chukwuka AV, Panigrahi AK, Saha NC, Faggio C: **Biochemical, physiological (haematological, oxygen-consumption rate) and behavioural effects of mercury exposures on the freshwater snail, Bellamya bengalensis**. *Comp Biochem Physiol C Toxicol Pharmacol* 2022, **251**:109195.

13. Pan Y, Qiu S, Chen R, Zhang T, Liang L, Wang M, Baloch AR, Wang L, Zhang Q, Yu S: **Molecular detection and phylogenetic analysis of porcine circovirus type 3 in Tibetan pigs on the Qinghai-Tibet Plateau of China**. *VIROL J* 2022, **19**(1):64.

14. Zhu HM, Luo SH, Gao M, Tao F, Gao JP, Chen HM, Li XY, Peng H, Ma YJ: **Phylogeny of certain members of Hyrcanus group (Diptera: Culicidae) in China based on mitochondrial genome fragments**. *INFECT DIS POVERTY* 2019, **8**(1):91.

15. Katsura Y, Stanley CJ, Kumar S, Nei M: **The Reliability and Stability of an Inferred Phylogenetic Tree from Empirical Data**. *MOL BIOL EVOL* 2017, **34**(3):718-723.

16. Patten MM, Carioscia SA, Linnen CR: **Biased introgression of mitochondrial and nuclear genes: a comparison of diploid and haplodiploid systems**. *MOL ECOL* 2015, **24**(20):5200-5210.

17. Cameron KM: **On the value of nuclear and mitochondrial gene sequences for reconstructing the phylogeny of vanilloid orchids (Vanilloideae, Orchidaceae)**. *Ann Bot* 2009, **104**(3):377-385.
