# Supplementary material for: Genetic differentiation of Oncomelania hupensis robertsoni in hilly regions of China: Using the complete mitochondrial genome
Source: PLoS Negl Trop Dis. 2024 Nov 26;18(11):e0012094. doi: 10.1371/journal.pntd.0012094 (PMC11630586; doi:10.1371/journal.pntd.0012094)
Supplement: Attachment — Submitted filename: response.pdf [file pntd.0012094.s004.pdf]

Manuscript Number: PNTD-D-24-00426R1) - [EMID: ffe34917ed79f78a]

Genetic differentiation of *Oncomelania hupensis robertsoni* in hilly regions of China: using the complete mitochondrial genome

Dear Prof. Luc E. Coffeng and Audrey Lenhart,

Thank you for giving us the opportunity to revise our manuscript. We have revised the manuscript and would like to re-submit it for your consideration. We have addressed the comments raised by the reviewer, and the amendments are highlighted in red in the revised manuscript.

We apologize for mistake the affiliation of the co-author Song Jing, and the funding of this study due to carelessness. We have changed the above in the revised manuscript.

We are highly appreciative of the careful critique of the reviewer and hope that the improvements in this manuscript have sufficiently addressed all of your concerns. Our point-by-point responses are listed below.

We look forward to hearing from you soon.

With kindest regards,

Yours sincerely,

Yi Dong

Correspondence:

Yi Dong

Department of Schistosomiasis Control and Prevention, Yunnan Institute of Endemic Disease Control and Prevention, 5 Wenhua Rd, Dali, 671000;

Email: dydali@sina.com

Response to Reviewers

Methods

Reviewer #1

Comment 1: Lines 157-163: Change to "Dead O. hupensis snails were selected and removed using the crawling method. Briefly, the snails were placed in the center of a petri dish with a diameter of 15cm, after which a few drops of water were poured over the snails. A mesh cover was placed over the petri dish to prevent the live snails from crawling out. The set up was placed at room temperature (25℃) over 24 hours, after which the ones that crawled from the center were regarded as live. Thirty live snails were used for DNA extraction.

Response 1: We truly appreciate your time in reviewing our article, and thanks for your useful suggestion. As suggested, we have revised these sentences. (please see page 9, lines 161-162 and page 10, lines 163-173 in Manuscript with track change)

Comment 2: Line 164: Please indicate the approximate size of the head-foot muscle used for DNA extraction (i.e., 2-mm strip, etc.)

Response 2: Thank you for your kind suggestion. The approximate size of the head-foot muscle used for DNA extraction is 7mm strip. As suggested, we have added the relevant information. (please see page 10, line 172 in Manuscript with track change)

Comment 3: Lines 212-221: based on the reply of the authors, they must include the model of DNA substitution used for ML and NJ. For example, they used the GTR+G model for ML. They must also specify the default models they used for NJ.

Response 3: Thank you for your kind suggestion. In this study, we used the General Time Reversible model for ML, and d: Transition + Transversions model for NJ. As suggested, we have added the model of DNA substitution used for ML and NJ. (please see page 13, lines 224-225 in Manuscript with track change)

Reviewer #2: (No Response)

Results

Reviewer #1

Comment 4: For figure 3, there were some singular values (100) on certain nodes instead of four (for ML/MP/ME/NJ). What do these singular values mean? Are they bootstrap supports for just one tree method? This needs to be clarified.

Response 4: Thank you for your kind suggestion. We apologize for this confusion. Indeed, values on nodes represent bootstrap support percentage for ML/MP/ME/NJ methods, with a single value (e.g., 100) representing the same boot support percentage for ML/MP/ME/NJ methods. As suggested, we have added the bootstrap support percentage for each method in Fig 3. to make it clearer. (please see Fig 3.)

Comment 5: Line 343: Remove the extra ‘,’ after ‘Additionally’

Response 5: Thank you for your kind suggestion. As suggested, we have we have revised this sentence. (please see page 20, line 375 in Manuscript with track change)

Reviewer #2: (No Response)

Conclusions

Reviewer #1

Comment 6: For the conclusion section: The response of the authors regarding how the mitochondrial heterogeneity of *O. hupensis robertsoni* from the samples they collected be correlated to their control was sufficient; however, it was not detailed much in the main manuscript. I hope the authors can better incorporate their response into the main manuscript.

Response 6: Thank you for providing this comment which enlarge the value of this manuscript. As suggested, we have further discussed how the genetic data of *O. hupensis robertsoni* can be used for schistosomiasis control in our manuscript. (please see page 18, lines 324-32 in Manuscript with track change)

Reviewer #2: (No Response)

Editorial and Data Presentation Modifications?

Reviewer #1: None.

Reviewer #2: (No Response)

Summary and General Comments

Reviewer #1

Comment 7: I have no further comments. The other technical comments have already been raised by the other reviewer, and I will leave it to the other reviewer to determine if the authors have sufficiently addressed the comments.

However, I would like to the authors to carefully consider their conclusion regarding the applicability of their data for control of the snail. They need to elaborate on this further. Please see my comments on their conclusion section.

I also hope the authors are able to addressed my issue on Figure 3, especially on the singular values on certain nodes as these can be quite confusing.

Response 7: Thank you for your kind and valuable Comments. As suggested, we have revised the content of Discussion section and Fig 3 to make it more reliable and clearer. (please see Manuscript with track change and Fig 3)

We really appreciate the time and the effort you put into our article. Your suggestions really mean a lot to us. We hope you will find this revised version satisfactory. Thanks again.

Reviewer #2

Comment 8: the author response to previous review comments are mostly effective, However, the response to comment 27 (included below) remains an issue:

The authors recognize, yet ignore an in-frame start codon upstream of the indicated reading frame for CYTB.

The argument that a previous Genbank uses the same shorter interval is not adequate, unless that annotation is based on protein-level confirmation of CYTB. The previous entry may be incorrect, resulting from not recognizing the 5' alternative startcodon. It is not benefiting science to continue annotation that ignores the "rules"

of the genetic code. I repeat the comment that the annotation should interpret the actual genetic code provided by the experimental data, not perpetuate potential errors in previous GenBank entries." Comment 27: 182 Mitos automated sequencing is a good start but annotation requires visual checking: for recommendations see: Fourdrilis et al., 2018; doi:10.1038/s41598-018-36428-7 and Ghiselli et al 2020; DOI: 10.1098/rstb.2020.0159.

For instance, your Genbank entry OR661787: the region between ND6 and CYTB includes one more potential start codon that would extend the CYTB ORF by three nucleotides and one AA,. Ignoring this start codon goes against the genetic code, and reports a trunctated CYTB 5' sequence, and provides an incorrect start codon (ATG versus ATT). This also impacts the discussion of the number of nucleotides in intergenic, non-coding regions. Please check ALL gene ORFs and revise accordingly.

ND6 CYTB

I * (M) M R

ATT TAG C ATT ATG CGC

Response 27: Thank you for your kind suggestion. As suggested, we have visual checked all mitochondrial genome sequences. The base of OR661787 sequence is GCATT between gene ND6 and CYTB gene. According to the literature provided by the reviewer, the literature does mention "ATY" as a possible codon. However, when we aligned the mitochondrial sequences in NCBI, we found that the start codon of the mitochondrial CYTB gene was ATG, so we used ATG as the start codon as well."

Response 8: We truly appreciate your time in reviewing our article, and thanks for your useful suggestion.

As suggested, we have performed sequencing experiment to confirm whether the in-frame start codon upstream of the indicated reading frame is ATT or ATG. We have extracted the RNA of *Oncomelania hupensis robertsoni* snail and reverse transcribed it into cDNA, designed the first and last amplification primers of CYTB gene according to sequence, amplified the cDNA and ligated the T vector for sequencing, and designed the forward primer from the vector for one generation sequencing.

According to result of sequencing experiment, the in-frame start codon upstream of the indicated reading frame for CYTB gene is ATT.

In addition, we have checked all the genes and only the CYTB gene has this problem with an incorrect start codon. We have revised the relevant information of CYTB gene and have contacted NCBI to revise the previously uploaded sequence information. (please see Fig.2 and eTable1-eTable9 in Additional file 2)

We would like to take this opportunity to thank you again for all your time involved and this great opportunity for us to improve the manuscript.
